# A spatially informed matrix normal model for gene co-expression analysis in spatial transcriptomics studies

**DOI:** 10.1093/nar/gkaf1264

**Published:** 2025-12-10

**Authors:** Chichun Tan, Ying Ma

**Affiliations:** Department of Biostatistics, Brown University, Providence, RI 02903,United States; Department of Biostatistics, Brown University, Providence, RI 02903,United States; Center for Computational Molecular Biology, Brown University, Providence, RI 02903,United States

## Abstract

The rapid advancement of spatially resolved transcriptomics (SRT) technology enables gene expression profiling across tissue locations while preserving spatial context. Gene co-expression analysis in SRT data provides critical insights into how genes function together within the tissue microenvironment. However, existing methods fail to effectively capture the joint influence of gene–gene interactions and spatial dependencies, limiting their biological interpretability. Here, we introduce spMOCA (SPatially informed Matrix-nOrmal model for gene Co-expression Analysis), a statistical framework for inferring gene co-expression networks while explicitly modeling spatial dependencies. By leveraging a matrix-normal model, spMOCA jointly accounts for gene–gene and spatial covariance, disentangling intrinsic co-expression relationships from spatially induced effects. Through extensive simulations, we show that spMOCA provides more accurate and unbiased estimates of gene–gene correlations than existing approaches across a range of spatial dependency levels. In applications to nine SRT datasets spanning diverse technologies, tissues, and species, spMOCA consistently identifies more experimentally validated transcription factor target genes than alternative methods. In tumors, it uncovers gene modules linked to tumorigenesis and immune pathways, revealing prognostic markers. In aging mouse brains, it captures dynamic co-expression changes associated with neurodegeneration. In cross-species analyses, it detects conserved gene modules and cell type–specific pathways in the mouse and human cortex.

## Graphical Abstract

**Graphical Abstract:**
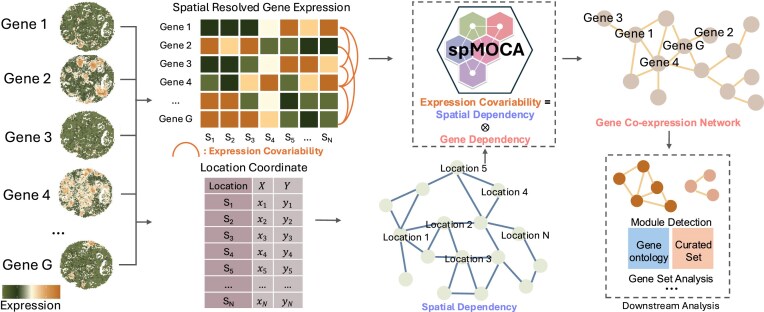

## Introduction

Gene co-expression analysis plays a fundamental role in understanding how genes interact and coordinate their functions across biological systems. By identifying groups of genes that exhibit correlated expression patterns, co-expression networks provide critical insights into molecular pathways, regulatory mechanisms, and disease processes. These analyses have been widely applied in bulk RNA sequencing (RNA-seq) and single-cell RNA-seq (scRNA-seq) studies, leading to important discoveries, such as functional gene modules involved in immune responses [1–3] and neurodegenerative diseases [4–6]. However, co-expression networks were inferred from these data without spatial context, as bulk RNA-seq homogenizes tissue samples, and scRNA-seq dissociates cells from their native environment before sequencing. This assumption fails to account for the role of spatial organization in shaping gene expression, which is influenced not only by underlying gene–gene regulatory interactions but also by spatially structured factors within tissues [7–9]. These factors—such as tissue architecture and microenvironmental influences—play a critical role in both normal physiology and disease progression. For example, in cancer, coordinated gene expression in immune cells varies with spatial proximity to cancer cells, affecting tumor progression and therapeutic response [10–12]. In brain, gene co-expression patterns are influenced by spatial dependencies across cortical and subcortical structures, shaping neuronal function and circuit organization [13]. These and numerous other studies [14–16] demonstrate that gene co-expression patterns are driven not only by regulatory mechanisms but also by spatial organization, underscoring the need to incorporate spatial dependencies when inferring co-expression networks.

Spatially resolved transcriptomics (SRT) overcomes these limitations of bulk and scRNA-seq by enabling gene expression profiling across multiple tissue locations while preserving spatial context. The integration of molecular and spatial information allows researchers to investigate a wide range of biological processes, including spatial organization of cell types [17, 18], and tissue architecture [19, 20], as well as to explore gene co-expression [21, 22] within the tissue environment. Among these applications, an important yet underexplored area is gene co-expression analysis, which reveals how genes coordinate their functions within a spatially structured tissue context. As SRT technologies continue to advance, there is growing interest in leveraging SRT data to study gene co-expression [14, 23, 24], reflecting the increasing recognition that spatial organization influences gene–gene relationships. Despite this potential, current approaches for inferring gene co-expression networks (GCNs) using SRT data fail to adequately incorporate spatial dependencies across tissue locations. Widely used methods, such as Pearson’s correlation or co-expression inference approaches developed for scRNA-seq data (e.g. CSCORE) [25], do not account for spatial organization when estimating gene–gene correlations. A few methods specifically designed for SRT data, such as SpaceX [21] and Giotto [22], attempt to incorporate spatial information by either adjusting for spatial effects through an additive framework or smoothing expression values across neighboring locations. However, both approaches have limitations. Smoothing-based methods risk over-smoothing bias, where excessive spatial averaging can obscure localized gene expression variations and introduce artificial correlations. Meanwhile, the additive framework assumes that spatial effects and gene–gene dependencies act independently, failing to capture their interactions. This oversimplifies the relationship between spatial organization and gene expression, potentially leading to misattribution of spatial expression variation to noise or technical artifacts, thereby biasing co-expression network inferences.

This gap underscores the need for methods that explicitly model the interaction between spatial and gene dependencies in SRT data. In gene co-expression analysis of microarray data, failing to account for contextual dependencies—such as experimental conditions [26] or tissue types [27]—has been shown to produce biologically misleading conclusions. Similarly, in developmental biology, gene regulatory networks dynamically change across spatial gradients due to local signaling pathways, a phenomenon that cannot be accurately captured by models that treat spatial and gene effects as independent. Other fields have long recognized the risk of ignoring one dimension of dependency when modeling two-dependent structures. For example, in neuroimaging [28, 29], time dependencies are modeled to distinguish functional networks from temporal proximity effects, ensuring that observed correlations reflect true neural connectivity. Likewise, in geospatial analyses [30, 31], spatial and temporal dependencies are explicitly modeled to prevent confounding in economic and environmental studies. SRT data present a similar challenge: inferred co-expression patterns may be influenced by spatial dependency in a way that obscures true biological interactions between genes. If spatial dependencies are not properly accounted for, gene–gene correlation estimates may be biased, either inflated due to shared tissue structure, where genes appear co-expressed simply due to their spatial colocalization, or distorted when spatial dependencies mask true gene–gene relationships.

To address this challenge, we introduce spMOCA (SPatially informed Matrix-nOrmal model for gene Co-expression Analysis), a statistical framework for inferring GCNs from SRT data. Unlike existing approaches that either adjust for spatial effects additively or smooth expression values across neighboring locations, spMOCA explicitly models the interaction between spatial structure and gene expression. By leveraging a matrix-normal distribution, spMOCA accounts for both gene–gene covariance and spatial covariance across tissue locations, allowing it to disentangle intrinsic co-expression relationships from spatially induced patterns. This joint modeling approach ensures that inferred gene networks reflect true biological interactions rather than artifacts of spatial proximity. Through comprehensive simulations, we demonstrate that spMOCA provides more unbiased and robust estimates of gene–gene correlations, particularly when gene and spatial dependencies interactively shape gene expression patterns. To further validate spMOCA’s performance, we applied it to nine SRT datasets spanning different tissues, species, and biological contexts. Our downstream analyses—including gene module detection, hub gene identification, and gene set analysis—revealed that GCNs inferred by spMOCA exhibited greater biological coherence than those produced by competing methods. In particular, spMOCA recovered a higher proportion of experimentally validated transcription factor (TF) target genes from databases such as TRRUSTV2 [32] and KnockTF [33], further supporting its biological accuracy. Notably, in cancer data, spMOCA identified gene modules associated with tumorigenesis-related and immune-related pathways, providing deeper insights into cancer progression and innate immune activity, where we identified novel prognostic genes from these modules’ hub genes across difference cancer types. In mouse brain aging datasets, spMOCA uncovered changes of gene co-expression relationships across time points associated with neurodegenerative processes, while in both mouse and human brain datasets, it revealed groups of co-expressed genes that are enriched in similar cell type–specific pathways across species. These findings highlight spMOCA’s ability to accurately capturing gene co-expression patterns within tissue microenvironments.

## Results

### spMOCA method overview

**Figure 1.**
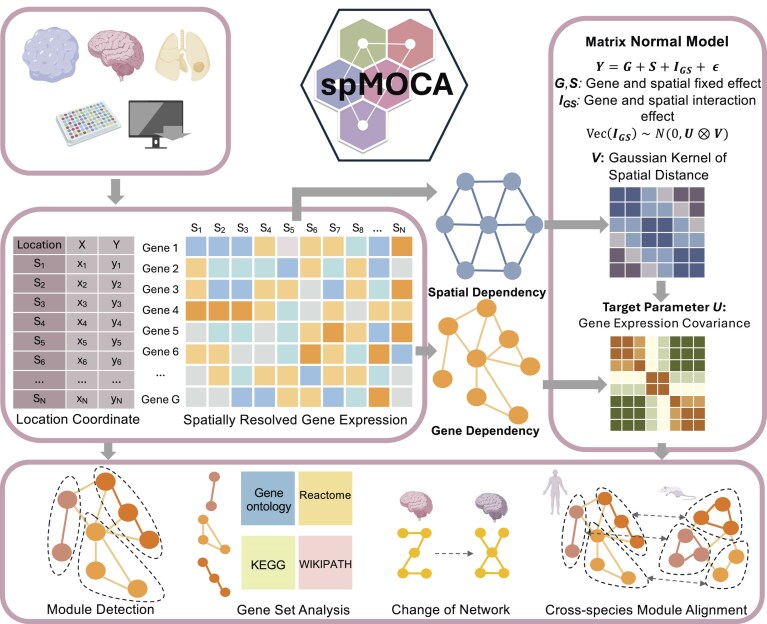
Schematic overview of spMOCA. spMOCA infers GCNs from SRT data and can be applied to datasets from various tissue types (top left box). It requires spatially resolved gene expression data and spatial coordinates as inputs (middle left box). Using these inputs, spMOCA simultaneously accounts for both gene–gene covariance and spatial covariance through a matrix-normal modeling framework. The output of spMOCA is a gene–gene covariance matrix (right box), which incorporates spatial dependencies. This matrix can be transformed into a gene–GCN for downstream analyses, including module detection, gene set analysis, network comparison, and module alignment (bottom).

The spMOCA method is described in Materials and methods with its technical details provided in Supplementary Note S1 and a schematic workflow in Fig. 1. Briefly, spMOCA is a spatially informed matrix normal model developed to infer GCNs across spatial locations while accounting for spatial dependencies. It models spatial transcriptomics data by simultaneously capturing spatial covariance across locations and gene covariance across genes, effectively integrating variation in gene expression across both dimensions. By leveraging these dual sources of covariance, spMOCA effectively reconstructs GCNs in a spatial context. Notably, the model captures the influence of gene–gene correlations and spatial dependencies within neighboring regions of tissue space, referred to as the “gene-spatial interaction effect,” which shapes spatial gene expression patterns. Built on a maximum likelihood framework with efficient matrix calculations, spMOCA is scalable to datasets with tens of thousands of spatial locations and genes. The method is implemented as an open-source R package, freely available at https://github.com/YMa-lab/spMOCA.

### Simulation study

We conducted comprehensive and realistic simulations to evaluate the performance of spMOCA and compared it with four other gene co-expression estimation methods. The compared methods in simulations include SpaceX [21], Giotto [22], CSCORE [25], and Pearson’s correlation. The details of simulation setup are described in Materials and methods and the detailed description of compared methods are provide in Supplementary Note S2. Briefly, we designed two simulation scenarios: we denote the first framework as the simulation with spatial-gene interactive dependencies based on the assumption of matrix normal distribution where the effects of gene dependencies and spatial dependencies interactively influence the SRT data; we denoted the second framework as the simulation with spatial-gene additive dependencies where the effects of gene dependencies and spatial dependencies independently add up to influence the SRT data. In both frameworks, we varied the strength of spatial dependency from weak, moderate to strong by varying the bandwidth in the spatial kernel (Materials and methods) to assess how different levels of spatial structure affect gene co-expression estimation. Specifically, each simulation scenario consists of five replicates to examine the variance of each method’s performance. To assess the robustness of spMOCA, we evaluated its performance in scenarios without true gene co-expression. Specifically, we simulated datasets with no gene correlations to test whether spMOCA and the competing methods incorrectly estimate nonzero correlations. In general, the simulated data are realistic, preserving data features observed in the real spatial transcriptomics data [34] (Supplementary Figs S1 and S2). We assessed the performance of the co-expression methods using five metrics to compare the estimated gene–gene correlation with the ground truth. These metrics include root mean squared error (RMSE), median absolute deviation (MAD), Pearson’s correlation coefficient (PCC), and RV coefficient (RV) for evaluating the accuracy of the estimated gene–gene correlations. Additionally, the Adjusted Rand Index (ARI) was used to evaluate how accurately the inferred co-expression networks recover the true gene modules. Further details on these metrics are provided in Materials and methods.

**Figure 2.**
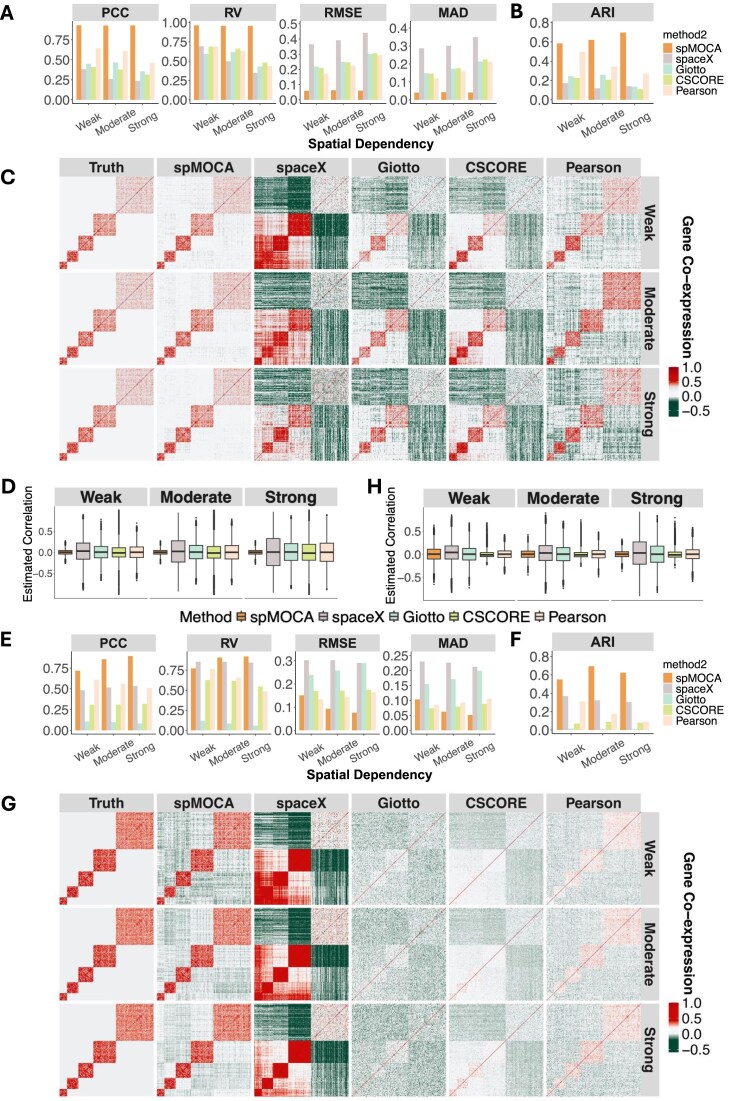
Evaluation of co-expression estimations in the simulation scenarios with spatial-gene interactive dependencies and additive dependencies. Evaluation results of co-expression methods in two spatial-gene dependencies settings. In the *interactive dependencies* setting, (**A**) accuracy evaluation of gene–gene correlation estimations using four metrics: PCC, RV coefficient, RMSE, and MAD. (**B**) ARI comparing inferred gene modules to true gene modules. (**C**) Heatmaps of gene–gene correlation estimations. (**D**) Estimated gene correlations when there is no true gene co-expression in simulated data. In the *additive dependencies* setting, (**E**) accuracy evaluation of gene–gene correlation estimations using four metrics: PCC, RV coefficient, RMSE, and MAD. (**F**) ARI comparing inferred gene modules to true gene modules. (**G**) Heatmaps of gene–gene correlation estimations. (**H**) Estimated gene correlations when there is no true gene co-expression in simulated data.

We first examined the simulation scenario when there are spatial-gene interactive dependencies. In this scenario, spMOCA outperformed the other methods in all five-evaluation metrics, regardless of weak, moderate, or strong spatial dependency (Fig. 2A). Notably, spMOCA’s estimated gene–gene correlation has the highest correlation with the true values (Supplementary Table S1) with mean PCC equals to 0.932 for weak, 0.927 for moderate, and 0.930 for strong dependency, compared to (0.643, 0.611, 0.462) for Pearson’s correlation, (0.381, 0.260, 0.234) for SpaceX, (0.449, 0.467, 0.356) for Giotto, and (0.411, 0.378, 0.314) for CSCORE (Fig. 2A). Other evaluation metrics, such as RV coefficient, RMSE, and mean MAD also consistently indicate that spMOCA’s estimated gene–gene correlation is more accurate [Fig. 2A, mean RV = (0.962, 0.956, 0.957), mean RMSE = (0.059, 0.063, 0.059), and mean MAD (0.040, 0.042, 0.040)] than other methods. To evaluate spMOCA’s effectiveness in identifying key gene modules (Materials and methods), we applied weighted GCN analysis (WGCNA) to the estimated gene–gene correlation matrix and assessed its accuracy in recovering true gene module labels. The results show that spMOCA consistently outperforms other methods in module detection. Specifically, spMOCA achieved mean ARI scores of 0.584 for weak, 0.62 for moderate, and 0.696 for strong dependency with WGCNA, compared to the second-best method, Pearson’s correlation, which scored 0.497, 0.345, and 0.27 (Fig. 2B). We also visualized the estimated gene–gene correlation matrices from each method by heatmaps (Fig. 2C). These heatmaps demonstrate that spMOCA’s high estimation accuracy is consistent across all gene–gene pairs, including both diagonal and off-diagonal blocks, and the performance is maintained across different levels of spatial dependencies. In contrast, the heatmaps depicting estimated gene–gene correlations from other methods show notable discrepancies when compared to the true heatmaps. These discrepancies become more obvious with increasing spatial dependency. Additionally, spMOCA provided more accurate correlation estimates in scenarios without gene–gene correlations across varying spatial dependency structures (Fig. 2D). In contrast, the other methods showed substantial variations, with more non-zero correlation estimations deviating from the true values, particularly as spatial dependency increased.

We further assessed whether spMOCA is robust to the selection of spatial kernel bandwidths in an estimation. Instead of using the true bandwidths that were used to simulate the data, we estimated kernel bandwidths for spMOCA following the procedures of spatialPCA [20] (Materials and methods). We found that spMOCA still maintains the good performance and outperforms the other methods across spatial dependencies [Supplementary Fig. S3A, mean PCC for spMOCA = (0.932, 0.801, 0.744 for weak, moderate, and strong dependency, respectively), mean RV = (0.963, 0.857, 0.785), mean RMSE = (0.059, 0.116, 0.135), and mean MAD = (0.040, 0.078, 0.091), Supplementary Table S1]. Consistently, spMOCA is more accurate in detecting the gene modules, achieving the highest ARI (mean ARI = 0.591 for weak, 0.679 for moderate, and 0.621 for strong dependency) (Supplementary Fig. S3B and Supplementary Table S1). The heatmap of spMOCA’s estimated gene–gene correlation patterns is consistent with that of the true co-expression pattern, across both diagonal and off-diagonal blocks (Supplementary Fig. S3C). Furthermore, spMOCA consistently demonstrated more accurate and less variable correlation estimations in the scenarios without gene–gene correlations across all levels of spatial dependency, outperforming all other methods (Supplementary Fig. S3D).

Next, we examined the simulation scenario with spatial-gene additive dependencies. Following the evaluation approach used for spatial-gene interactive dependencies scenario, we assessed spMOCA’s performance using both the true bandwidth and the estimated bandwidth. Despite releasing the assumption of gene-spatial interaction, spMOCA consistently performed as the top methods in all evaluation metrics for both bandwidth inputs. In contrast, methods such as SpaceX, CSCORE, and Pearson’s correlation, performed well in only one or two evaluation metrics (Fig. 2E, Supplementary Fig. S4A, and Supplementary Table S2). For example, mean PCC of spMOCA’s results was 0.718, 0.857, and 0.895 using the true bandwidth (Fig. 2E) and 0.718, 0.710, and 0.706 using the estimated bandwidth (Supplementary Fig. S4A), for weak, moderate, and strong spatial dependency, respectively, much higher than the other methods. Similar, for RV coefficients, spMOCA achieved high RV values comparable to SpaceX’s (0.853, 0.853, 0.844) for both bandwidth inputs [the true bandwidth = (0.771, 0.907, 0.919) and the estimated bandwidth = (0.771, 0.774, 0.773)]. While SpaceX achieved a slightly higher RV than spMOCA when the latter used the estimated bandwidth—likely due to its modeling assumption closely aligning with additive dependencies [21]—its performance remained inconsistent across evaluation metrics, with the worst RMSE and MAD among all methods. Similarly, Pearson’s correlation, and CSCORE have a lower RMSE and MAD, but did not perform well in PCC and RV coefficients, while Giotto failed to perform well across all evaluation metrics and spatial dependency levels (Fig. 2E, Supplementary Fig. S4A, and Supplementary Table S2). Additionally, spMOCA demonstrated the highest gene module detection accuracy, achieving the highest ARI across both bandwidth selections (Fig. 2F, mean ARI = 0.55 for weak, 0.694 for moderate, 0.623 for strong dependency and Supplementary Fig. S4B, 0.543 for weak, 0.642 for moderate, and 0.586 for strong dependency). Furthermore, the heatmap of spMOCA’s estimated gene–gene correlation patterns closely matched the true co-expression pattern, more accurately capturing gene–gene correlation patterns in both diagonal and off-diagonal blocks than the other methods (Fig. 2G and Supplementary Fig. S4C). Moreover, spMOCA, CSCORE, and Pearson’s correlation exhibited greater stability in correlation estimations and produced fewer false nonzero correlations in scenarios without true gene–gene correlations, regardless of spatial dependency levels (Fig. 2H and Supplementary Fig. S4D). Overall, only spMOCA consistently performed well across all evaluation metrics and scenarios. These findings highlight spMOCA’s superior performance in accurately estimating gene–gene correlations and identifying modules, demonstrating its robustness to the bandwidth selection.

### 10X visium cancer data

We applied spMOCA and the other methods to analyze nine published datasets from different tissues and SRT technologies, including four 10X Visium SRT data from four different cancer types, three 10X Visium data from mouse whole brain tissue of three different ages [35], and two MERFISH data from a human cortex tissue and a mouse cortex tissue [36] (Supplementary Table S3). SpaceX was excluded from all data analysis because it did not converge within the maximum allowed computation time: for example, when SpaceX was applied to the smallest dataset 10X Visium Breast Cancer with 2518 spots, it could not finish the analysis with 2 days and 32G memory. Details of the data preprocessing were described in Materials and methods.

**Figure 3.**
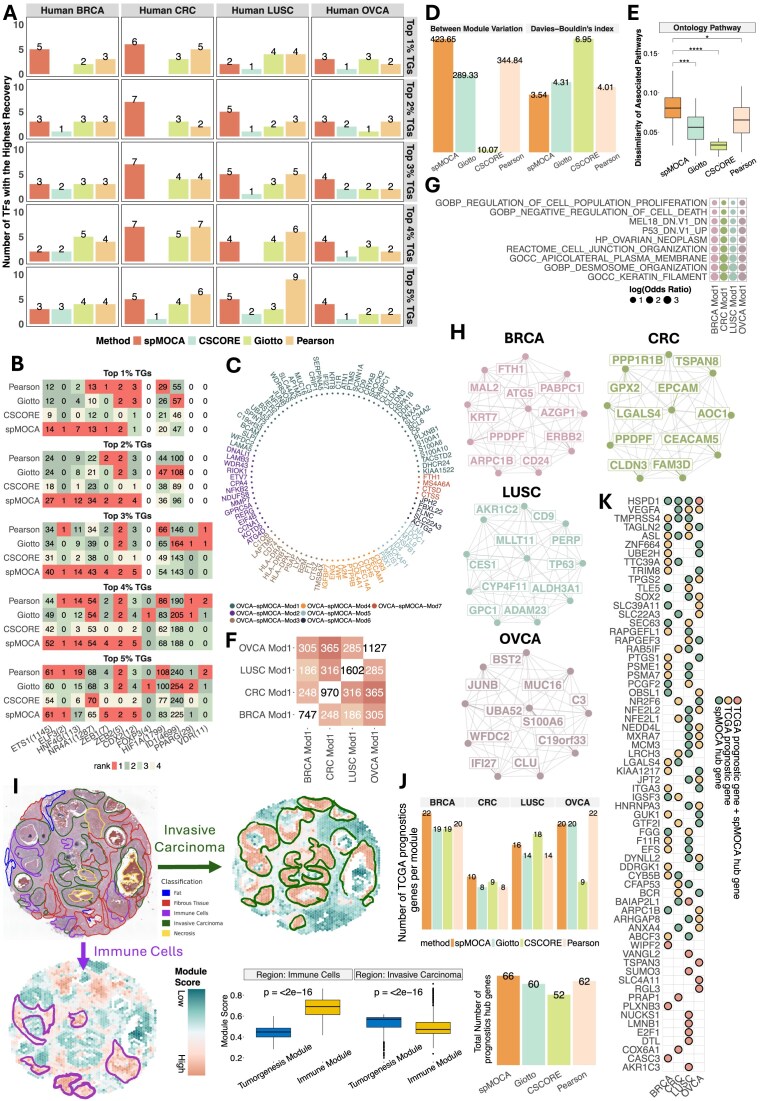
GCN cross-tumor analysis in 10X Visium cancer data. (**A**) The number of TFs for which each method achieved the highest recovery of known TF–target gene (TG) interactions within their top TG gene set ranked by co-expression strength. (**B**) The number of recovered known TF–TG pairs for each TF in the human CRC dataset. The number besides each TF name indicates the total number of known TGs within the data. (**C**) Top 1% hub genes of spMOCA’s gene modules in OVCA. (**D**) Bar plots showing Separation (higher is better) and the Davies–Bouldin index (lower is better) for OVCA gene modules based on gene expression levels. Separation is defined by between-module variation. (**E**) Boxplot illustrating the dissimilarity of OVCA module-associated Gene Ontology pathways, quantified by Hamming distance (see Materials and methods). *P*-values are calculated using a one-sided Wilcoxon rank sum test. *: *P*-value < 0.05; **: *P*-value < 0.01; ***: *P*-value < 0.001; ****: *P*-value < 10^−4^. (**F**) Pairwise counts of shared pathways between tumorigenesis-related spMOCA modules. (**G**) Selected significant pathways shared across all spMOCA tumorigenesis-related modules. (**H**) Top 10 hub genes in spMOCA’s tumorigenesis-related modules. (**I**) Comparison between the expert-annotated H&E image of BRCA tissue from the 10X Genomics platform and spatial maps of spMOCA tumorigenesis-related and immune-related module scores. Green and purple areas indicate the regions with high tumorigenesis-related and immune-related module scores respectively. Boxplots of tumorigenesis-related module score and immune-related module score in Invasive Carcinoma region and Immune Cells region. *P*-value was obtained by two-tails Wilcoxon Rank test. (**J**) Bar plots showing the counts of overlaps between hub genes and TCGA prognostics genes from each co-expression method for each tumorigenesis-related gene module in each data (above) and across all tumorigenesis-related gene modules. (**K**) Overlap between TCGA-derived tumor prognostic genes and spMOCA tumorigenesis-related hub genes. A circle indicates that a gene is a hub gene in the corresponding module. Green coloring indicates that the gene is identified as a hub gene by spMOCA only; Yellow coloring indicates that the gene is identified as a prognostics gene from TCGA data only; Red coloring indicates that the gene is identified as a hub gene by spMOCA and as a TCGA-derived tumor prognostic gene (see Materials and methods).

First, we analyzed the 10X Visium cancer dataset, which includes four tumor tissue samples: breast ductal carcinoma (BRCA), colorectal cancer (CRC), lung squamous cell carcinoma (LUSC), and ovarian carcinoma (OVCA). For each dataset, we first estimated gene co-expression values using different methods (Materials and methods). To validate biological relevance of our estimated gene co-expression values, we used the known TFs from two widely cited databases—TRRUSTV2 [32] and KnockTF [33]—and assessed whether their experimentally supported target genes appeared among the top genes ranked by co-expression strength in GCNs. In this context, for each TF, we referred a set of its top-ranked genes as TGs, and we used the count of the known target genes among the TGs to define the recovery of known TF–TG regulatory relationships. Details of the validation process is provided in Supplementary Note S4. Our rationale is that a biologically meaningful co-expression network should place the known TGs among the most strongly associated genes of their corresponding TFs, thereby enriching for true regulatory targets and reducing the likelihood of false-positive associations. Compared to CSCORE, Giotto and Pearson, we found that spMOCA achieved the top TF–TG recovery in most of the tumor-specific TFs across all tumor data, especially when we examined very top-ranked TGs (Fig. 3A). This suggests that gene pairs with strong co-expression in spMOCA networks are more likely to correspond to true transcriptional regulatory relationships than those identified by alternative approaches. We then closely examined per-TF recovery across datasets. For example, *ETS1*, a key oncogenic TF functioning in tumorigenesis [37–39], showed the highest number of recovered known TGs in all tumor datasets across all ranked TGs when analyzed with spMOCA, except for LUSC, where *ETS1* was not identified as a TF (Fig. 3B and Supplementary Fig. S5). These results demonstrated that spMOCA reliably prioritizes biologically supported regulatory interactions across a wide range of TFs and cancer types.

We then performed module detection analysis to identify groups of co-expressed genes (Materials and methods). Since biologically related genes often exhibit coordinated expression patterns, an accurate co-expression network should reveal modules that reflect underlying gene–gene interactions and functional groupings. To assess how well each method captured these structures, we first evaluated the separation and distinctiveness of the detected modules by measuring the variation between modules and computing the Davies–Bouldin index, which quantifies how clearly the modules are delineated within the network (Materials and methods). We found that the spMOCA’s GCNs yielded more distinct and better-separated gene modules compared to those derived from other methods (Fig. 3C and Supplementary Figs S6–S9). For example, in the OVCA dataset, spMOCA displayed the highest between-module variation and the lowest Davies–Bouldin index, indicating that its GCNs detected gene modules that were more clearly differentiated and distinct from each other, outperforming other methods (Fig. 3D). Similar patterns of high between-module variation and low Davies–Bouldin index were also observed in other cancer datasets (Supplementary Fig. S10). These metrics suggest that spMOCA’s GCNs are more effective at delineating groups of genes with similar or dissimilar expression patterns. Next, we performed gene set analysis for each identified gene module to evaluate its association with tumor-context-relevant pathways, providing insights into its correspondence with distinct biological processes within the tumor microenvironment (TME). To assess if these modules capture unique functional relationships, we calculated the Hamming distance based their significantly associated pathways (Materials and methods). This step was essential to ensure that inferred gene modules not only exhibit clear expression pattern’s separation but also align with known biological pathways, reinforcing the accuracy and biological relevance of the co-expression networks. We found that in OVCA data, spMOCA’s modules exhibited significantly greater dissimilarity in Gene Ontology pathways compared to all competing methods (Fig. 3E). Similar trends were observed across the other three cancer datasets, where spMOCA’s modules generally showed higher dissimilarity, indicating more functional differences between modules (Supplementary Fig. S11). The observed significant separations in biological pathways suggest that spMOCA effectively distinguishes gene modules based on their specific functional roles.

Previous Pan-cancer analyses [40–42] have revealed that groups of genes often engage in similar biological pathways across diverse cancer types. Building on these findings, we examine whether gene modules extracted from spMOCA’s GCNs shared similar pathways across different cancers. To investigate this, we focused on hub genes, defined as those with the highest connectivity within each module, as key drivers of the module’s biological functions (Materials and methods). Specifically, we assessed shared functional properties by computing pairwise overlaps of biological pathways that were significantly associated with hub genes for each module pair across all tumors (Supplementary Fig. S12). This analysis identified two distinct clusters of gene modules, each characterized by a high degree of pathway overlap (Fig. 3F and Supplementary Fig. S13): the first cluster contains the gene modules BRCA Mod1, CRC Mod1, LUSC Mod1, and OVCA Mod1, while the second included BRCA Mod4, CRC Mod2, LUSC Mod2, and OVCA Mod3. We further explored the biological contexts of their sharing pathways. We found that the first cluster is strongly associated with tumorigenesis-related pathways (Fig. 3G), particularly those involved in cell structure formation, which plays a critical role in progression. Specifically, these gene modules are associated with pathways such as keratin-based filaments [GOCC_KERATIN_FILAMENT, *P*-values = (2.689 × 10^−4^, 9.883 × 10^−4^, 1.551 × 10^−6^, 5.371 × 10^−4^) for BRCA Mod1, CRC Mod1, LUSC Mod1, and OVCA Mod1, respectively] and plasma membrane composition [GOCC_APICOLATERAL_PLASMA_MEMBRANE, *P*-values = (4.195 × 10^−3^, 4.412 × 10^−6^, 8.311 × 10^−4^, 4.718 × 10^−3^)] (Fig. 3G). Additionally, these gene modules are associated with oncogenic signatures, such as MEL18 [MEL18_DN.V1_DN, *P*-values = (1.425 × 10^−2^, 2.291 × 10^−4^, 3.925 × 10^−2^, 6.829 × 10^−6^)] [43] and P53 [P53_DN.V1_UP, *P*-values = (3.956 × 10^−7^, 6.854 × 10^−12^, 1.814 × 10^−4^, 2.289 × 10^−11^)] [44] (Fig. 3G). Key hub genes identified such as *ERBB2* [45] in BRCA Mod1, *EPCAM* [46] in CRC Mod1, *TP63* [47] in LUSC Mod1, and *MUC16* [48, 49] in OVCA Mod1 that play pivotal roles in malignant cell proliferation, further supported that the first cluster is corresponding to tumorigenesis-related modules (Fig. 3H). The biological relevance of these modules was further validated by comparing their module scores across tissue locations with the proportions of various cell types (Materials and methods). We visualized the module scores across tumor tissues and observed that regions with high tumorigenesis-related module scores were distinctly localized within specific areas (Supplementary Fig. S14). Notably, these regions exhibited a strong correspondence with areas of high tumor cell density (Supplementary Figs S15–S19). For instance, in BRCA, the module score of BRCA Mod1 is higher in the invasive carcinoma region (Supplementary Fig. S15) and highly correlates with the proportion of HER2 + subtype tumor cell (Supplementary Fig. S16, Cancer_Her2_SC, PCC = 0.779). Similarly, in CRC, the module score of CRC Mod1 showed a strong correlation with consensus molecular subtypes (CMS3) tumor cell abundance (Supplementary Fig. S17, PCC = 0.901). The strong associations also exhibited between LUSC Mod1’s and OVCA Mod1’s module scores and their tumor cell type proportions (Supplementary Fig. S18, Malignant cell for LUSC, PCC = 0.778; Supplementary Fig. S19, Cancer cell for OVCA, PCC = 0.707). The second cluster was enriched for immune-related pathways (Supplementary Fig. S20), including shared pathways involved in immune signaling and regulation, such as antigen processing (KEGG_ANTIGEN_PROCESSING_AND_PRESENTATION, *P*-values = (3.515 × 10^−9^, 9.605 × 10^−7^, 3.667 × 10^−4^, 2.421 × 10^−18^) for BRCA Mod4, CRC Mod2, LUSC Mod2, and OVCA Mod3) and immune system activation [Module 170, *P*-values = (2.411 × 10^−4^, 1.296 × 10^−10^, 1.341 × 10^−12^, 4.791 × 10^−15^)] (Supplementary Fig. S20A). Key hub genes (Supplementary Fig. S20B) including *CD68* [50] in BRCA, *CD74* [51] in CRC and OVCA, and *TIMP1* [52] in LUSC, played critical roles in immune-cancer cell interactions and tumorigenesis inhibition. Moreover, immune-related module scores are highly (Supplementary Fig. S20C) correlated with the spatial distribution of specific immune cell types. For example, in BRCA, the module score of BRCA Mod4 is correlated with the immune cell-type distribution including T cells, B cells, and macrophages (Supplementary Fig. S16, PCC = 0.797); while in OVCA, the module score of OVCA Mod3 is highly correlated with the proportion of myeloid cells (Supplementary Fig. S19, PCC = 0.798), which involved in immune activities reported by the original paper [49]. Notably, we observed that the tumorigenesis‐related and immune‐related module scores (Supplementary Figs S14 and S20C) exhibit distinct spatial distributions in each tumor section (Supplementary Figs S15–S19), indicating distinct functional zones. For example, in BRCA, where we have an expert-annotated tissue region map (Fig. 3I), we found that the immune-related module scores were significantly higher in immune cells regions (Fig. 3I, Wilcoxon rank-sum test, *P* < 2 × 10^−16^), while tumorigenesis-related module scores were significantly higher in invasive carcinoma regions (Fig. 3I, *P* < 2 × 10^−16^).

To further investigate the clinical relevance of the gene modules identified by spMOCA, we examined their contribution to cancer prognosis. Following [53], we extracted prognostic genes that significantly predict survival outcomes for each tumor type from the TCGA database [54] (Materials and methods). A previous study [55] demonstrated prognostic roles of some genes involved in tumorigenesis. Motivated by this finding, we first examined how hub genes from tumorigenesis-related modules overlap with prognostic genes. We compared the hub genes of spMOCA’s tumorigenesis-related module with those from other co-expression methods’ tumorigenesis-related modules (Supplementary Fig. S21), where we define the tumorigenesis-related modules for the other methods by the same abovementioned procedure as applied to spMOCA. Across all tumor types, spMOCA’s hub genes consistently exhibited the highest overlap with TCGA-derived prognostic genes—recovering 66 such genes, compared to 62 for Pearson’s correlation, 60 for Giotto, and 52 for CSCORE (Fig. 3J). For example, in the BRCA, 22 of spMOCA’s hub genes overlapped with TCGA prognostic genes, compared to 19, 19, and 20 for Giotto, CSCORE, and Pearson’s correlation, respectively (Fig. 3J). Similar trends were observed in CRC, where spMOCA recovered the highest number of prognostic genes. In LUSC, spMOCA performed in top two methods comparably to CSCORE. In OVCA, spMOCA performed comparably to Giotto and Pearson (Fig. 3J). The results demonstrated that spMOCA’s tumorigenesis-related hub genes could exhibit higher prognostic values than the other methods. We further investigated contexts of these spMOCA’s hub genes, where we identified specific prognostic genes serves as hub genes in the same tumor type, for example, *WIPF2* and *CASC3* for BRCA, *COX6A1* and *PRAP1* for CRC, *E2F1* and *AKR1C3* for LUSC, and *TSPAN3* and *SLC4A11* for OVCA (Fig. 3K). Beyond confirming known prognostic genes within individual cancer types, we also explored whether spMOCA could identify prognostic markers that are shared across multiple cancers, potentially indicating broader clinical relevance. We identified *HSPD1*—previously recognized as prognostic in ovarian cancer based on TCGA data—was also identified as spMOCA’s hub genes across all four cancer types (Fig. 3K). Previous studies have confirmed the prognostic relevance of *HSPD1* [56, 57] in ovarian cancer, aligning the findings from TCGA data. However, spMOCA further suggested that it may also play similar prognostic roles in additional cancer types that were not detected by TCGA. This broader relevance is supported by prior studies linking *HSPD1* to breast [58], nonsmall cell lung [59], and colorectal cancers [60]. Similarly, spMOCA could extend the prognostic relevance of *VEGFA*—already known in LUSC [61] and OVCA [62]—to CRC [63], where it is a prognostic gene in both LUSC and served as a hub gene in CRC. We also extended our analysis to spMOCA’s immune-related modules’ hub genes, where we found 9 in BRCA, 1 in LUSC, and 17 in OVCA identified as TCGA prognostics genes (Supplementary Fig. S22). We identified specific prognostic genes serves as immune-related hub genes, such as *ITGB2* for BRCA and *LAPTM5* for OVCA (Supplementary Fig. S20D). Moreover, we found *PSAP*, like *HSAD1*, which was identified as a prognostics gene in OVCA and served as hub genes for all four cancer types (Supplementary Fig. S20D). The prognostics relevance of *PSAP* has been demonstrated for ovarian cancer [64], while spMOCA enabled us to generalize PSAP’s prognostics value to breast [65] and non-small cell lung cancers [66], where these broader relevance are supported by previous studies. Together, these findings illustrate that spMOCA not only recovers known cancer type–specific prognostic genes but also identifies hub genes with potential prognostic relevance across multiple cancers, underscoring its value for uncovering generalizable biomarkers to support personalized cancer therapy strategies.

### 10X visium aging mouse brain data

**Figure 4.**
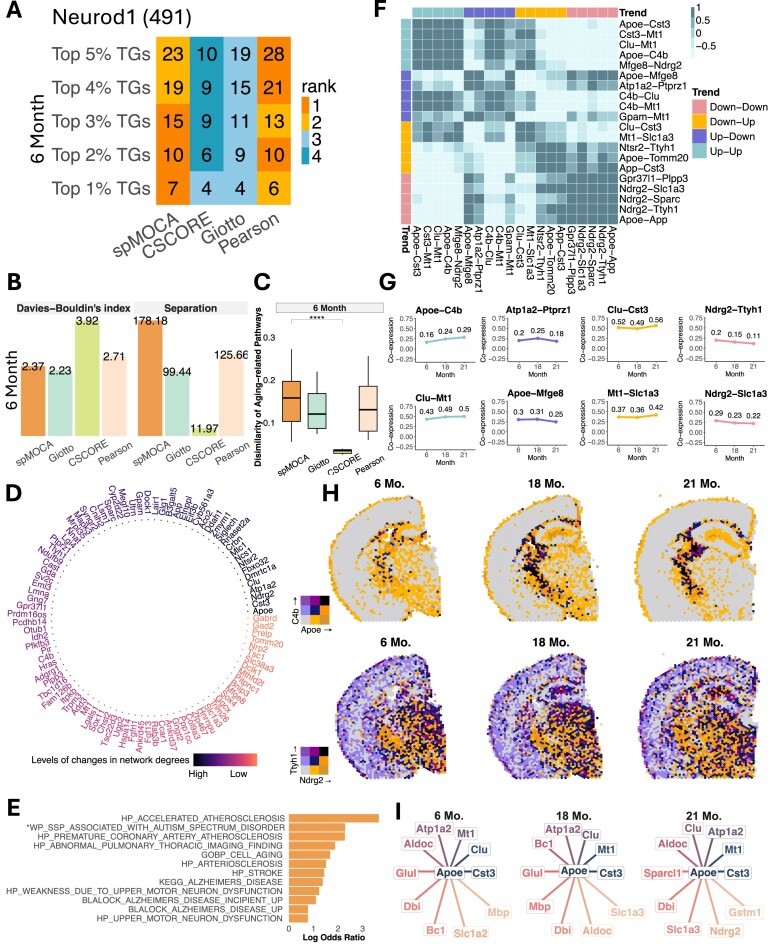
GCN analysis in 10X Visium aging mouse brain. (**A**) The number of recovered known TF–TG pairs for Neurod1 in the 6-month-old mouse data. The number besides each TF name indicates the total number of known TGs within the data. (**B**) Bar plots showing Separation (higher is better) and the Davies–Bouldin index (lower is better) for gene modules in the 6-month-old mouse. Separation is defined by between-module variation. (**C**) Boxplot illustrating the dissimilarity of the 6-month-old mouse module-associated aging-related pathways, quantified by Hamming distance (see Materials and methods). *P*-values are calculated using a one-sided Wilcoxon rank sum test; ****: *P*-value < 10^−4^. (**D**) Top degree-variable genes identified in spMOCA’s GCNs, ranked and colored by variations in their global degree across time points. (**E**) Significant pathways associated with the top degree-variable genes identified in spMOCA. *The full gene set name is WP_SYNAPTIC_SIGNALING_PATHWAYS_ASSOCIATED_WITH_AUTISM_SPECTRUM_DISORDER. (**F**) Heatmap showing the correlation of gene–gene co-expression changes across time points, with gene pairs clustered based on their co-expression pattern changes. (**G**) Line plots displaying co-expression pattern changes across ages for representative gene pairs. (**H**) Bivariate plots illustrating the spatial distribution of gene expression for a pair of genes. The first row shows Apoe-C4b, and the second row shows Ndrg2-Ttyh1, where darker regions indicate stronger co-expression between the two genes. (**I**) Neighboring genes of Apoe in spMOCA’s GCNs at different ages. The lengths of edges are determined by the gene co-expression value between Apoe and its neighboring genes, with shorter edges indicating stronger co-expression relationships.

Next, we applied spMOCA to a 10X Visium mouse whole brain SRT dataset, which included samples from a 6-month-old, an 18-month-old, and a 21-month-old mouse. Our goal was to uncover aging-related biological processes in the mouse brain using GCNs. Therefore, for each dataset, we inferred GCN for 1917 age-related genes provided by the original study [35], all of which were also spatially variable genes (SVGs) in all time points (Materials and methods). Following the same evaluation approach as in the cancer data, we first assessed the recovery of known TF–TGs (Supplementary Note S4) for the 6-month data, which represents the closest available match to the developmental stages in the TF–TG database. For *Neurod1* which was the only TF achieving recovery of known TF–TGs in this data, we found spMOCA showed the highest recovery outperforming all other methods in the top 1%–3% TGs and ranking among the top two methods in the 4%–5% TGs (Fig. 4A). This finding underscores spMOCA’s better capacity of capturing true transcriptional regulatory relationship, consistent with its performance in 10X Visium cancer datasets. We then evaluated spMOCA’s ability to identify well-separated gene modules by performing module detection for each inferred GCN at each age stage. In the 6-month-old mouse, spMOCA’s gene module exhibited the highest between-module variation while achieving a lower Davies–Bouldin index comparable to Giotto (Fig. 4B). In the 18-month-old and 21-month-old mice, spMOCA’s gene modules consistently showed the highest between-module variation among all methods, while their Davies–Bouldin index remained lower, comparable to that of Giotto or Pearson’s correlation (Supplementary Fig. S23). These findings emphasize spMOCA’s advantages in distinguishing groups of genes with similar and dissimilar expression patterns. Furthermore, we examined the functional dissimilarity of spMOCA’s gene modules by analyzing their significantly associated aging-related pathways. Our results revealed that spMOCA consistently maintained high pathway separation between most gene modules at the 6-month-old (Fig. 4C) and all other ages (Supplementary Fig. S24), indicated that the inferred GCNs capture distinct functional gene modules associated with aging.

To further understand the biological mechanisms underlying aging, we analyzed changes in the network degrees across ages. Specifically, we identified top degree-variable genes, which are defined as the genes that display the largest variation in connectivity within spMOCA’s GCNs across different age stages (Fig. 4D, Materials and methods). We found that the top-degree variable genes were associated with aging-related pathways including cellular aging (GOBP_CELL_AGING, *P*-value = 2.88 × 10^−2^), dysfunction pathways (HP_WEAKNESS_DUE_TO_UPPER_MOTOR_NEURON_ DYSFUNCTION, *P*-value = 1.23 × 10^−2^), and neurological diseases-related pathways, such as Alzheimer’s disease (KEGG_ALZHEIMERS_DISEASE, *P*-value = 2.8 × 10^−2^), atherosclerosis (HP_ARTERIOSCLEROSIS, *P*-value = 4.13 × 10^−2^), stroke (HP_STROKE, *P*-value = 4.83 × 10^−2^), and autism spectrum disorder (WP_SYNAPTIC_SIGNALING_PATHWAYS_ ASSOCIATED_WITH_AUTISM_SPECTRUM_ DISORDER, *P*-value = 7.96 × 10^−3^) (Fig. 4E). This highlights spMOCA’s effectiveness in identifying key gene markers that may contribute to the complexity of aging and its associated disorders.

We further examined age-related changes in gene–gene correlations within the mouse brain co-expression network. Specifically, we analyzed how gene–gene correlations evolved over time and identified shared patterns in these changes. By tracking pairwise co-expression trends among the top degree-variable genes, we categorized these changes into four distinct patterns: up–up, up–down, down–up, and down–down, where “up” indicates an increase, and “down” a decrease in co-expression values between consecutive time points. To investigate the biological significance of these trends, we extracted the top 5 gene pairs with the largest correlation changes from each category (Fig. 4F). Notably, “up–up” gene pairs, such as *Apoe*-*C4b, Clu*-*Mt1*, and *Cst3-Mt1*, exhibited highly similar co-expression trends. Previous studies have identified *Apoe* as a key marker for neurodegenerative diseases [67, 68] while *Clu* [69], *Cst3* [70], and *Mt1* [71] were involved in neuroprotection during aging. The observed “up–up” trend suggests these genes become increasingly co-expressed with age, possibly reflecting a coordinated response to aging-related neurodegenerative process in the mouse brain. We further examined one representative gene pair, *Apoe*-*C4b*, which displayed a steady increase in gene–gene correlation from 6–18 month to 18–21 month (Fig. 4G). This trend was consistent with previous finding [35] showing a smooth increase in both *Apoe* and *C4b* expression levels in the corpus callosum region. To further examine this, we generated a bivariate spatial expression plot (Fig. 4H), which confirmed that the increasing correlation corresponded to a growing overlap of their expression patterns in the corpus callosum regions (dark spots) across aging. While previous research has shown that *Apoe* and *C4b* are co-expressed in complement pathways within Alzheimer’s disease–specific co-expression networks [72], our findings extend this knowledge by providing spatial context to their co-expression dynamics over time. We also examined changes in the nearest neighbor genes of *Apoe* within the GCNs and found that these neighboring genes remained consistent across different ages (Fig. 4I). Genes such as *Cst3* (correlation = (0.57, 0.66, 0.66) for 6-month, 18-month, and 21-month) and *Clu* (correlation = (0.53, 0.57, 0.57)) displayed persistent co-expression with *Apoe* over time. Given their known roles in neurodegeneration and neuroprotection, this stability may reflect a conserved regulatory network involved in aging-related brain processes.

In contrast to the increasing correlation patterns observed in the “up–up” category, “down–down” gene pairs, such as *Ndrg2-Sparc* and *Ndrg2-Ttyh1*, exhibited highly similar co-expression trends over time (Fig. 4F). These genes play crucial roles in neural development and function, including cell differentiation [73], stem cell regulation [74], and synaptic maintenance [75]. Although direct interactions between these genes are not well-documented, the observed decline in their co-expression suggested a progressive deterioration of neural regulatory functions in the aging brain [76, 77]. To further investigate this pattern, we examined the *Ndrg2-Ttyh1* gene pair, which exhibited a steady decrease in gene–gene correlation from 6–18 months to 18–21 months (Fig. 4G). A bivariate spatial expression plot revealed that their expression patterns became increasingly distinct over time, with a decreasing proportion of dark spots across the brain (Fig. 4H). This suggests that the genes become less co-expressed with age, possibly due to shifts in cell types, changes in gene regulation, or aging-related neuronal loss. The reduced overlap may indicate a weakening of their functional connection in aging process.

### Mouse and human MERFISH brain cortex data

We conducted a cross-species gene co-expression analysis on human and mouse brain cortex data [36] generated by MERFISH, an imaging-based spatial transcriptomics technology [78]. Building on previous studies [79, 80] that identified similarities in gene modules between humans and mice, we constructed GCNs for overlapping genes across both species. We then applied gene module detection to evaluate the homogeneity of cross-species gene modules in terms of their shared genes and associated pathways derived from SRT data at single-cell resolution.

**Figure 5.**
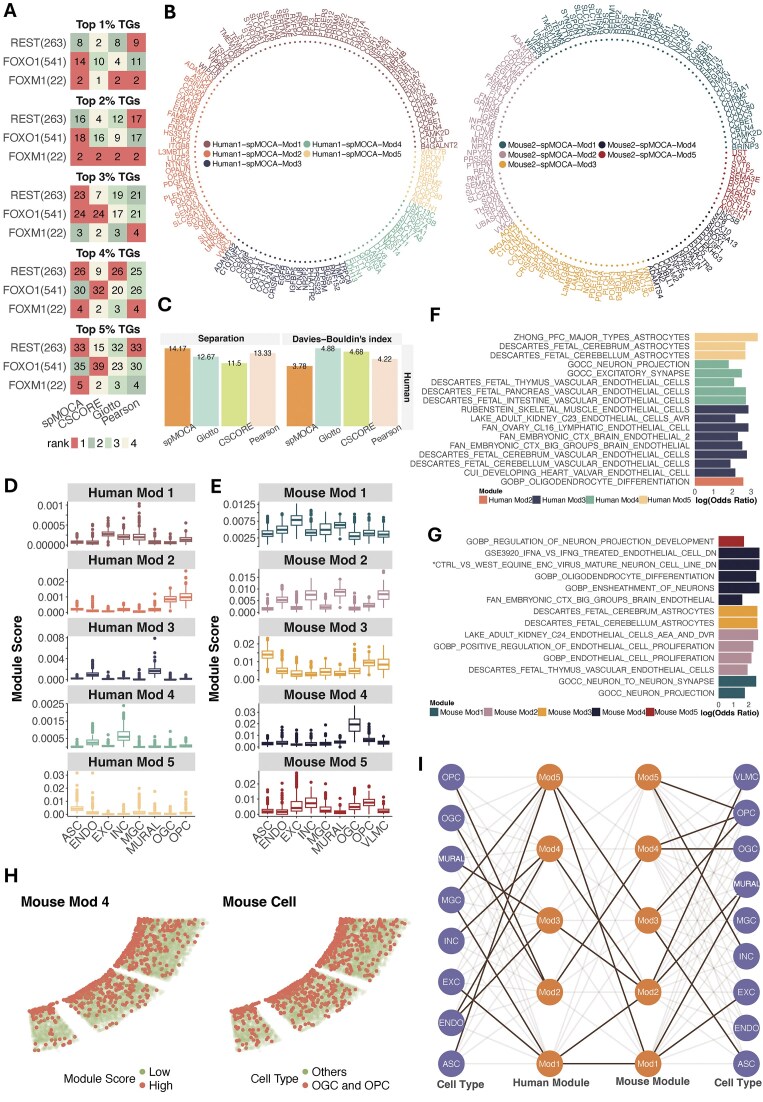
GCN cross-species analysis in MERFISH mouse and human brain cortex data. (**A**) The number of recovered known TF–TG pairs for each TF in the human dataset. The number besides each TF name indicates the total number of known TGs within the data. (**B**) spMOCA’s GCNs constructed for overlapping genes between human and mouse data. The overlapping genes are colored by different color indicating their module membership. (**C**) Bar plots showing Separation (higher is better) and the Davies–Bouldin index (lower is better) for human gene modules based on gene expression levels. Separation is defined by between module variation (**D**) Boxplots displaying spMOCA’s cell type–specific module scores in human data. (**E**) Boxplots displaying spMOCA’s cell type–specific module scores in mouse data. EXC: excitatory neurons; INC: inhibitory neurons; ASC: astrocytes; MGC: microglial cells; OGC: oligodendrocytes; OPC: oligodendrocyte progenitor cells; ENDO: endothelial cells; MURAL: mural cells; VLMC: mural cells and vascular smooth muscle. (**F**) Bar plot showing cell type–specific pathways significantly associated with spMOCA’s modules in human data. (**G**) Bar plot showing cell type–specific pathways significantly associated with spMOCA’s modules in mouse data. *The full gene set name is GSE16451_CTRL_VS_WEST_EQUINE_ENC_VIRUS_MATURE_NEURON_CELL_LINE_DN. (**H**) Spatial distribution of Mod4 module scores and OGC/OPC cells in mouse tissue. Cells with the top 10% Mod4 scores are defined as high module score cells. (**I**) Edge graph illustrating module-module and module-cell-type associations across the two species. Nodes in the inner columns represent gene modules, while nodes in the outer columns represent cell types. An edge connecting a human module to a mouse module indicates that the mouse module shares one of the top two highest numbers of genes with the human module. An edge connecting a module to a cell type indicates that the cell type has one of the top two highest scores for that module.

To benchmark the accuracy of GCN estimation, we used the same evaluation approach as in the cancer data. We first evaluated the recovery of known TF–TGs (Supplementary Note S4) for the human data, as the corresponding mouse data included a limited gene panel, resulting in no overlapping TF–TG pairs with the reference databases. We found that spMOCA most often achieved the highest recovery for TFs among all methods across all ranked TGs (Fig. 5A). Specifically, spMOCA achieved the highest TF–TG recovery for the most brain-specific TFs compared to the other methods, including physiological process regulator *FOXO1* [81] and neuron regulator *REST* [82] (Fig. 5A). These results demonstrate spMOCA consistently outperform in recovering true transcriptional regulation relationships than other methods. We then assess how well each method clustered genes into distinct modules corresponding to cell type–related pathways. In both human and mouse datasets, spMOCA’s gene modules (Fig. 5B) exhibited superior separation compared to those identified by the other methods (Supplementary Figs S25–S27). Specifically, in the human dataset, spMOCA’s gene modules demonstrated the highest between-module variation and the lowest Davies–Bouldin index among all methods (Fig. 5C). In the mouse dataset, spMOCA’s modules exhibited the highest between-module variation and a lower Davies–Bouldin index comparable to the one from Pearson’s correlation (Supplementary Fig. S28). These results indicate that spMOCA outperforms competing methods in identifying clusters of genes with shared or distinct expression patterns, consistent with its superior performance in the 10X Visium data. We further examined how the distinct separation of spMOCA’s gene modules relates to the heterogeneity across various cell type–specific pathways. In the mouse dataset, gene modules identified by spMOCA exhibited significantly greater separation in cell type–related pathways compared to those derived from Giotto and Pearson’s correlation, while showing a similar level of separation to CSCORE. In the human dataset, spMOCA’s modules also demonstrated greater separation between most gene modules than other methods (Supplementary Fig. S29). The higher dissimilarity suggests that spMOCA better distinguishes genes associated with different cell type–specific pathways compared to other methods.

Building on the gene modules identified by spMOCA, we investigated their ability to reveal shared gene pathways across species and to align cell types between human and mouse. To do this, we calculated cell-type level module scores (Materials and methods, Fig. 5D and E) and compared cell type–specific pathways between corresponding modules (Fig. 5F and G). The analysis identified gene modules associated with similar pathways across species. For example, Human Mod3, which showed a high module score in endothelial and mural cells (Fig. 5D), and Mouse Mod2, which exhibited a high module score in mural cells and vascular smooth muscle cells (Fig. 5E), were both significantly associated with the endothelial-cell type–specific pathways. Specifically, pathways associated with Human Mod 3 include FAN_EMBRYONIC_CTX_BRAIN_ ENDOTHELIAL_2 (*P*-value = 5.38 × 10^−4^) and FAN_EMBRYONIC_CTX_BIG_GROUPS_BRAIN_ ENDOTHELIAL with (*P*-value = 2.51 × 10^−4^) (Fig. 5F). Similarly, pathways associated with Mouse Mod 2 include GOBP_ENDOTHELIAL_CELL_PROLIFERATION (*P*-value = 9.35 × 10^−3^) (Fig. 5G). Spatial mapping of module scores further showed the distribution patterns of Human Mod3 and Mouse Mod2 scores aligned with the distribution of endothelial, mural and vascular smooth muscle cells in both tissues, and aligned with inhibitory neurons in mouse tissue (Supplementary Fig. S30). These modules shared eight genes, including *KCNJ8* and *TRPC6*, which are involved in endothelial cell activity by regulating ATP-sensitive potassium channels [83] and calcium channel [84], respectively (Fig. 5B and I). A similar cross-species alignment was observed in oligodendrocyte-associated modules. Human Mod2 and Mouse Mod4 both exhibited high module scores in oligodendrocytes (Fig. 5D and E), and were significantly associated with the pathway GOBP_OLIGODENDROCYTE_DIFFERENTIATION (Human Mod2, *P*-value = 1.39 × 10^−2^; Mouse Mod4, *P*-value = 1.41 × 10^−2^) (Fig. 5F and G). There was an evident consistency between the spatial patterns of these modules’ scores and the distribution of oligodendrocytes (Fig. 5H and Supplementary Fig. S30). These two modules shared 12 genes, including *SOX10* [85], *OPALIN* [86], and *ENPP6* [87], which play crucial roles in oligodendrocyte differentiation (Fig. 5B and I).

## Discussion

We have presented spMOCA, a new statistical method for accurate gene co-expression inference in SRT studies. Built on a Matrix Normal Model, spMOCA simultaneously accounts for both spatial and gene dependencies in SRT data. Through comprehensive simulations, we evaluated spMOCA’s performance against existing methods under two different scenarios: one where gene expression was influenced by interactive spatial-gene dependency and another where it was influenced by additive spatial-gene dependency. Our results demonstrated that spMOCA provides more accurate estimates of gene–gene correlations, particularly under interactive spatial-gene dependency. Furthermore, spMOCA maintained high accuracy even when spatial and gene effects influenced expression independently, highlighting its robustness across diverse gene-spatial relationships. By effectively modeling spatial dependency, spMOCA outperformed competing methods, producing reliable and unbiased gene–gene correlation estimates, making it a robust tool for analyzing coordinated gene activity within tissue microenvironments.

We have demonstrated that spMOCA-derived GCNs provide biologically relevant insights across both sequencing-based (e.g. 10X Visium) and imaging-based (e.g. MERFISH) SRT platforms. Through extensive downstream analysis, we showed that spMOCA’s GCNs help identify distinct gene modules that are significantly associated with biological pathways. In the 10X Visium cancer dataset, spMOCA identified modules enriched for tumorigenesis pathways and immune-related processes, highlighting functionally distinct gene groups involved in the TME and uncovering shared genes with prognostic value across different cancer types. In the 10X Visium aging mouse brain dataset, spMOCA’s GCNs captured key aging-related changes in gene co-expression relationships, further demonstrating its ability to reveal biologically meaningful patterns across diverse contexts. In the MERFISH cross-species dataset, spMOCA uncovered shared gene modules that characterize corresponding cell types in the human and mouse cortex, reinforcing the method’s ability to align biological structures across species.

We found that gene modules exhibited distinct spatial expression patterns. In the 10X Visium breast cancer dataset, tumorigenesis-related and immune-related module scores closely aligned with their respective functional regions, while also delineating separate spatial domains. These patterns were consistent with results from domain detection methods applied to the same data [88], suggesting that GCNs could be leveraged to improve spatial domain detection. Indeed, a recent study [89] applied GCNs constructed from overlaps of genes’ spatially differential expression to identify contiguous domains. However, this work primarily focused on domain detection rather than GCN modeling, and it did not address how different measures of gene co-expression might influence domain detection performance. Beyond this, only a limited number of studies [90, 91] have highlighted the potential contributions of GCNs to spatial domain detection, indicating that their roles have not been fully discussed. Therefore, further exploration and systematic benchmarking represent important future directions for advancing the integration of GCNs in spatial transcriptomics.

There are several important future extensions for spMOCA. First, we employed a Gaussian kernel [92] with Silverman’s bandwidth selection rule [93] to model spatial dependency. While our results indicate that spMOCA’s performance is largely robust to variations in bandwidth values across settings, exploring alternative kernels, such as periodic kernels and cosine kernels [92], could capture diverse spatial correlation patterns and thereby enable spMOCA to uncover different aspects of gene co-expression relationships in SRT data. Second, spMOCA currently assumes a unified gene co-expression relationship across the entire tissue space. However, GCNs can vary across spatial regions [94]. While region-specific co-expression networks can be inferred by applying spMOCA to subsampled tissue areas defined by pre-specified domain labels [21, 94, 95], this approach depends on the accuracy of domain annotations. A promising future direction would be to extend spMOCA into a mixture model, allowing unsupervised inference of region-specific GCNs without requiring predefined spatial domains.

## Materials and methods

### spMOCA method overview

We provide an overview of spMOCA, with its technical details available in Supplementary Note S1. spMOCA is designed to model gene expression data that can be generated from various SRT technologies. It is built on a spatially informed matrix normal model for inferring GCN while accounting for spatial covariance, gene covariance, and gene-spatial location interactions. The input to spMOCA consists of SRT data containing gene expression measurements for $G$ genes across $N$ spatial locations. Specifically, we denote $Y \in {{\mathbb{R}}^{G \times N}}$ as the gene expression matrix. Following [96–98], we assume that the expression measurements have already been normalized by library size factor of each location and processed by a log-transformation. Additionally, we have the 2-dimensional spatial coordinates $L \in {{\mathbb{R}}^{N \times 2}}$ for the measured spatial locations in the tissue. Specifically, we model the gene expression matrix $Y$ by:


(1) \begin{eqnarray*}
Y = X + S + {{I}_{GS}} + \epsilon
\end{eqnarray*}

where $X$ and $S$ are $G$-by-$N$ matrices representing the fixed effects from genes and spatial locations respectively, and $\epsilon $ represents a matrix of small random noises arising from all nuisance sources with each element independently and identically following a normal distribution ${{\epsilon }_{gi}} \sim N( {0,\sigma _e^2} )$. ${{I}_{GS}}$ is the gene-locations interaction effect, whose vectorized format $vec( {{{I}_{GS}}} )\ $follows a multivariate normal distribution:


(2) \begin{eqnarray*}
vec\left( {{{{\boldsymbol{I}}}_{{\boldsymbol{GS}}}}} \right) \sim {\boldsymbol{MVN}}\left( {0,{\boldsymbol{U}} \otimes {\boldsymbol{V}}} \right)
\end{eqnarray*}

where ${\boldsymbol{U}} \in {{\mathbb{R}}^{{\boldsymbol{G}} \times {\boldsymbol{G}}}}$ and ${\boldsymbol{V}} \in {{\mathbb{R}}^{{\boldsymbol{N}} \times {\boldsymbol{N}}}}$ represents the covariance matrix between genes and spatial locations respectively, $ \otimes $ is the Kronecker product, and ${\boldsymbol{vec}}()$ represents the vectorization function. This model captures three key assumptions regarding SRT data: (i) gene-specific expression profiles across locations are not independent, and their covariance structure is captured by the spatial covariance ${\boldsymbol{V}}$ matrix, which encodes spatial correlations between locations; (ii) location-specific expression profiles across genes are not independent, and their covariance structure is captured by the gene–gene covariance ${\boldsymbol{U}}$ matrix, which encodes gene co-expression relationships; (iii) gene expression variation arises from a combination of gene-specific effects, spatial influences, and their joint dependencies. This is modeled using a matrix normal distribution, where the Kronecker-structured covariance ${\boldsymbol{U}} \otimes {\boldsymbol{V}}$ governs both gene–gene and spatial dependencies. The random noise term $\epsilon $ is assumed to be small or negligible, following previous studies on gene expression modeling [26, 27]. Under this formulation, the expression matrix ${\boldsymbol{Y}}$ follows a matrix normal distribution, where ${\boldsymbol{Y}} \sim {\boldsymbol{MN}}( {{\boldsymbol{M}},{\mathrm{\ }}{\boldsymbol{U}},{\mathrm{\ }}{\boldsymbol{V}}} )$, with the mean matrix value ${\boldsymbol{M}}$ defined as ${\boldsymbol{vec}}( {\boldsymbol{M}} ) = {\boldsymbol{vec}}( {{\boldsymbol{X}} + {\boldsymbol{S}}} ).$

Following the assumption of negligible nuisances ε, as applied in previous works [26, 27], we have


(3) \begin{eqnarray*}
{\boldsymbol{vec}}\left( {\boldsymbol{Y}} \right) \sim {\boldsymbol{N}}\left( {{\boldsymbol{vec}}\left( {\boldsymbol{M}} \right),{\boldsymbol{U}} \otimes {\boldsymbol{V}}} \right)
\end{eqnarray*}

where ${\boldsymbol{vec}}( {\boldsymbol{Y}} )$ follows a multivariate normal distribution with mean ${\boldsymbol{vec}}( {\boldsymbol{M}} ) = {\boldsymbol{vec}}( {{\boldsymbol{G}} + {\boldsymbol{S}}} )$ and covariance matrix ${\boldsymbol{U}} \otimes {\boldsymbol{V}}$. An important assumption in spatial transcriptomics is that gene expression at nearby locations is correlated. This spatial dependency has been observed in previous studies on spatial transcriptomics data [17, 19–21]. To capture co-variability in gene expression introduced by spatial proximity, we construct the spatial covariance matrix ${\boldsymbol{V}}$ using spatial location information. Our primary goal is to infer the gene–gene covariance by estimating the ${\boldsymbol{U}}$ matrix while accounting for spatial dependency through ${\boldsymbol{V}}$. Specifically, we construct ${\boldsymbol{V}}$ through a gaussian kernel function following [20], with each element ${{{\boldsymbol{V}}}_{{\boldsymbol{ij}}}}$ defined as:


(4) \begin{eqnarray*}
{{{\boldsymbol{V}}}_{{\boldsymbol{ij}}}} = {\boldsymbol{exp}}\left( { - \frac{{{{{\left| {\left| {{{{\boldsymbol{L}}}_{\boldsymbol{i}}} - {{{\boldsymbol{L}}}_{\boldsymbol{j}}}} \right|} \right|}}^2}}}{{\begin{array}{@{}*{1}{c}@{}} \tau \\ \ \end{array}}}} \right)
\end{eqnarray*}

where ${{{\boldsymbol{L}}}_{\boldsymbol{i}}},{{{\boldsymbol{L}}}_{\boldsymbol{j}}}$ are the coordinates of spot ${\boldsymbol{i}}$ and ${\boldsymbol{j}}$, and ${{\bf \tau }}$ is the bandwidth parameter controlling the strength of the spatial dependency. The gaussian kernel introduces stronger spatial dependency of gene expression patterns in neighboring locations. The distribution in (3) also implies that both the gene-specific expression profiles (rows of ${\boldsymbol{Y}}$) and the location-specific expression profiles (columns of ${\boldsymbol{Y}}$) follow multivariate normal distributions (Supplementary Note S1).

We derived Maximum Likelihood Estimators (MLEs) for estimating the mean gene expression ${\boldsymbol{M}}$, the gene covariance ${\boldsymbol{U}}$, and the gene–gene correlation matrix (also denoted as GCN) ${{{\boldsymbol{R}}}_{\boldsymbol{U}}}$. Following [26], we assume that the spatial location effects ${\boldsymbol{S}}$ have zero mean. This assumption enables us to attribute the mean level of gene expression solely to the gene context, thereby avoiding redundant calculations that would arise from combining mean levels attributed to both spatial and gene contexts. Therefore, we have ${\boldsymbol{M}} = {\boldsymbol{\mu }}{{1}^{\boldsymbol{T}}}$, where ${{\bf \mu }}$ is ${\boldsymbol{G}}$-by-$1$ vector of gene-specific means, and $1$ is ${\boldsymbol{N}}$-by-$1$ vector of ones. The log-likelihood function for Equation (3) is:


(5) \begin{eqnarray*}
{\boldsymbol{l}}\left( {{\boldsymbol{Y}};{\boldsymbol{M}},{\boldsymbol{U}},{\boldsymbol{V}}} \right) &=& - \frac{{\boldsymbol{G}}}{2}\log \left| {\boldsymbol{V}} \right| - \frac{{\boldsymbol{N}}}{2}\log \left| {\boldsymbol{U}} \right| \\&-& \frac{1}{2}{\boldsymbol{tr}}\left( {\left( {{\boldsymbol{Y}} - {{\bf \mu }}{{1}^{\boldsymbol{T}}}} \right){{{\boldsymbol{V}}}^{ - 1}}{{{\left( {{\boldsymbol{Y}} - {{\bf \mu }}{{1}^{\boldsymbol{T}}}} \right)}}^{\boldsymbol{T}}}{{{\boldsymbol{U}}}^{ - 1}}} \right)
\end{eqnarray*}

To maximize this log-likelihood, we compute the first partial derivatives with respect to ${\boldsymbol{U}}\ $and ${\boldsymbol{\mu }}$.


(6) \begin{eqnarray*}
\frac{{\partial {\boldsymbol{l}}}}{{\partial {\boldsymbol{U}}}} = \ - \frac{{\boldsymbol{N}}}{2}{{{\boldsymbol{U}}}^{ - 1}} + \frac{1}{2}\left( {{{{\boldsymbol{U}}}^{ - 1}}\left( {{\boldsymbol{Y}} - {\boldsymbol{\mu }}{{1}^{\boldsymbol{T}}}} \right){{{\boldsymbol{V}}}^{ - 1}}{{{\left( {{\boldsymbol{Y}} - {\boldsymbol{\mu }}{{1}^{\boldsymbol{T}}}} \right)}}^{\boldsymbol{T}}}{{{\boldsymbol{U}}}^{ - 1}}} \right)\\
\end{eqnarray*}

(7) \begin{eqnarray*}
\frac{{\partial {\boldsymbol{l}}}}{{\partial {\boldsymbol{\mu }}}} = \ {{{\boldsymbol{U}}}^{ - 1}}\left( {{\boldsymbol{Y}} - {{\bf \mu }}{{1}^{\boldsymbol{T}}}} \right){{{\boldsymbol{V}}}^{ - 1}}1
\end{eqnarray*}

Setting these derivatives to zero yields the following MLEs:


(8) \begin{eqnarray*}
{\boldsymbol{\hat{\mu }}} = \frac{{{\boldsymbol{Y}}{{{\boldsymbol{V}}}^{ - 1}}1}}{{{{1}^{\boldsymbol{T}}}{{{\boldsymbol{V}}}^{ - 1}}1}}
\end{eqnarray*}

(9) \begin{eqnarray*}
{\boldsymbol{\hat{U}}} = \frac{1}{{\boldsymbol{N}}}\left( {{\boldsymbol{Y}} - {\boldsymbol{\hat{\mu }}}\ {{1}^{\boldsymbol{T}}}} \right){{{\boldsymbol{V}}}^{ - 1}}{{\left( {{\boldsymbol{Y}} - {\boldsymbol{\hat{\mu }}}\ {{1}^{\boldsymbol{T}}}} \right)}^{\boldsymbol{T}}}
\end{eqnarray*}

We denote ${\boldsymbol{W}}$ as the diagonal matrix with the same diagonal terms of ${\boldsymbol{\hat{U}}}$. According to the invariance property of MLE estimator, the MLE of the gene–gene correlation matrix ${{{\boldsymbol{R}}}_{\boldsymbol{U}}}$ is


(10) \begin{eqnarray*}
\widehat {{{{\boldsymbol{R}}}_{\boldsymbol{U}}}} = {{{\boldsymbol{W}}}^{ - 1/2}}{\boldsymbol{\hat{U}}}{{{\boldsymbol{W}}}^{ - 1/2}}
\end{eqnarray*}

### Software implementation

We provide an implementation of spMOCA within R available on Github (https://github.com/YMa-lab/spMOCA). Built under R 4.2.2, the package leverages Rcpp to accelerate large‐matrix multiplications. Detailed dependency information can be found on the GitHub repository, and a step‐by‐step tutorial for applying spMOCA to SRT datasets is available at https://yma-lab.github.io/spMOCA/. spMOCA has been tested and runs successfully on macOS Sequoia 15.0.1 and Linux Red Hat 7.

### Simulation design

We performed comprehensive simulations to evaluate the performance of spMOCA and other GCN estimation methods including SpaceX, Giotto, CS-CORE, and Pearson’s correlation. To do so, we designed two frameworks for simulating spatial transcriptomics data based on different relationships between gene dependency and spatial dependency: (i) The first scenario, which we denote as the simulation with spatial-gene interactive dependency, assumes that gene dependency and spatial dependency jointly influence gene expression. Specifically, we model gene expression using a matrix normal distribution, where the covariance structure is determined by both gene–gene and spatial dependencies. This framework aligns with the core assumption of spMOCA, which incorporates both gene and spatial dependencies when modeling gene expression variation. (ii) The second scenario we denoted as the simulation with spatial-gene additive dependency, where we assume that gene dependency and spatial dependency contribute to gene expression independently, with the former influencing location-specific expression profiles across genes and the latter influencing gene-specific expression profiles across spatial locations. This scenario releases the key assumption in spMOCA that gene and spatial dependency interactively contribute to variations in gene expression. To ensure that our simulations are realistic, we obtained a publicly available breast cancer (BC) spatial transcriptomics dataset [34] and extracted the gene-level variance from its SVGs obtained by SPARK [92]. The simulated data retains the essential characteristics observed in the original dataset (Supplementary Figs S1 and S2). Details of the simulation procedure are introduced in the following sections.

#### Simulation with spatial-gene interactive dependency

In the simulation with spatial-gene interactive dependency, we first obtained the spatial locations from the BC data, which contains 250 spatial locations. Then we obtained the top 250 SVGs from SPARK [92]. Based on the real spatial locations, and the set of SVG genes, we simulated the spatial transcriptomics data ${\boldsymbol{Y}}$ following the matrix normal distribution in Equation (3). Specifically, the simulation process involved generating a gene-level mean matrix ${\boldsymbol{M}}$, a gene covariance matrix ${\boldsymbol{U}}\ $and a spatial covariance matrix ${\boldsymbol{V}}$ as follows:

1. For the mean-value matrix ${\boldsymbol{M}}$, we first normalized the gene expression counts by library size factor, and then we took a log-normalization and focused on the subset of the normalized expression data of these SVGs. Then, we calculated gene-level means ${\boldsymbol{\mu }}$ from the normalized data. The mean-value matrix ${\boldsymbol{M}}$ is constructed by ${\boldsymbol{M}} = {\boldsymbol{\mu }}{{1}^{{\bf T}}}$, where $1$ is a 250-by-1 all-one vector.
2. For the gene covariance matrix ${\boldsymbol{U}}$, we followed CS-CORE [25] to generate a block-structured covariance matrix based on a stochastic block model [99]. Specifically, we first defined five gene modules with sizes varying from 20, 30, 40, 60 to 100. Within each gene module, a binary adjacent network was generated with an edge probability of 0.8, while no edge exists between genes from different modules. The adjacency matrix was generated by the *sample_sbm()* function from Package *igraph* [100] taking the edge probability and the gene modules size as inputs. After we obtained the adjacency matrix, a gene correlation matrix ${\boldsymbol{{\mathrm{P}}}}$ was constructed such that gene pairs with edges have correlations of 0.8 following the degree of correlation in CS-CORE’s simulation setting. To generate a more realistic gene covariance matrix ${\boldsymbol{U}}$, we calculated the sample covariance ${\boldsymbol{U}}{\mathrm{^{\prime}}}$ from the normalized data of the 250 SVGs$,\ $then constructed ${\boldsymbol{U}}$ by setting its diagonal terms ${{{\boldsymbol{U}}}_{{\boldsymbol{ii}}}}$ equal to the diagonal terms ${\boldsymbol{U}}_{{\boldsymbol{ii}}}^{\mathrm{^{\prime}}}$ of ${\boldsymbol{U}}{\mathrm{^{\prime}}}$, where ${\boldsymbol{U}}_{{\boldsymbol{ii}}}^{\mathrm{^{\prime}}}$ is the sample variance of gene ${\boldsymbol{i}}$, and setting its off-diagonal terms ${{{\boldsymbol{U}}}_{{\boldsymbol{ii^{\prime}}}}} = \ {{{\boldsymbol{\rho }}}_{{\boldsymbol{ii}}^{\prime}}}*\sqrt {{\boldsymbol{U}}_{{\boldsymbol{ii}}}^{\mathrm{^{\prime}}}{\boldsymbol{U}}_{{\boldsymbol{i^{\prime}i^{\prime}}}}^{\prime}} $, where ${{{\boldsymbol{\rho }}}_{{\boldsymbol{ii}}^{\prime}}}$ is the simulated correlation between gene ${\boldsymbol{i}}$ and gene ${\boldsymbol{i}}^{\prime}$ from ${\boldsymbol{{\mathrm{P}}}}$ and ${\boldsymbol{U}}_{{\boldsymbol{i^{\prime}i^{\prime}}}}^{\prime}$ is the sample variance of gene ${\boldsymbol{i}}^{\prime}$, based on the definition of PCC.
3. For the spatial covariance matrix ${\boldsymbol{V}}$, we followed SpatialPCA [20] to construct the ${\boldsymbol{V}}$ using a gaussian kernel defined in Equation (4). Specifically, we calculated the Euclidean distances between the spatial locations based on the 2D spatial coordinates obtained from the BC data. To evaluate each method’s performance under different spatial dependency patterns, spatial kernels were constructed with three levels of strength: weak, moderate, and strong by varying the bandwidth parameter ${{\bf \tau }}$ from 0.3, 0.5 to 1. The range of ${{\bf \tau }}$ is selected according to the spatial gaussian kernel used in SpatialPCA [20].

Given a gene expression covariance matrix ${\boldsymbol{U}}$, a spatial covariance ${\boldsymbol{V}}$ and a mean-value matrix ${\boldsymbol{M}}$, we applied *rmatnorm()* function from the R package *matrixNormal* [101] to generate the normalized gene expression data ${\boldsymbol{Y}}$. To compare co-expression estimation methods that take count data as input, such as CS-CORE, Giotto, and SpaceX, we converted the simulated normalized gene expression into a count matrix. Specifically, we took the exponential of each entry in the simulated normalized gene expression matrix and sampled the count values from a Poisson distribution using the exponential value as the rate. This procedure is widely used in existing gene expression data simulator such as Splatter [102] and SCRIP [103] to derive count data from normalized count data.

#### Simulation with a spatial-gene additive dependency

To demonstrate the robustness of spMOCA, we designed the simulation scenario incorporating a spatial-gene additive dependency, which violated the assumption of matrix normal distribution. Following the additive model of SpaceX [21], we assumed that gene dependency and spatial dependency additively contributed to gene expression. Specifically, SpaceX models the observed gene expression count ${{{\boldsymbol{Y}}}_{{{\bf g}},{{\bf n}}}}$ for gene ${\boldsymbol{g}}$ in spatial location ${\boldsymbol{n}}$ as a Poisson random variable with a normalizing factor ${{{\boldsymbol{M}}}_{\boldsymbol{n}}}$ and rate parameter ${{{\boldsymbol{\Lambda }}}_{{{\bf g}},{{\bf n}}}}$,


\begin{eqnarray*}
{{{\boldsymbol{Y}}}_{{\boldsymbol{g}},{\boldsymbol{n}}}} \sim {\boldsymbol{Poi}}\left( {{{{\boldsymbol{M}}}_{\boldsymbol{n}}}{{{\boldsymbol{\Lambda }}}_{{{\bf g}},{{\bf n}}}}} \right)
\end{eqnarray*}

and assumes the rate parameter ${\boldsymbol{\Lambda }} = [ {{{{\boldsymbol{\Lambda }}}_{{{\bf g}},{{\bf n}}}}} ]$ is then modeled with an additive log-linear equation


\begin{eqnarray*}
\log ( {\boldsymbol{\Lambda }} ) = {\boldsymbol{S}} + {\boldsymbol{\Phi F}}
\end{eqnarray*}

where ${\boldsymbol{S}}$ is a *G*-by-*N* matrix of spatial effect, ${\boldsymbol{\Phi }}$ is a *G*-by-*K* loading matrix, and ${{\bf F}}$ is a *K*-by-*N* factor matrix. Inspired by this additive structure, given the gene-level mean ${\boldsymbol{\mu }}$, the gene covariance matrix ${\boldsymbol{U}}$ and the spatial kernel matrix ${\boldsymbol{V}}$ generated by the aforementioned procedure, we simulated the spatially resolved gene expression as follows:

1. First, we generated a 250-by-1 gene-specific expression profiles vector for each location ${\boldsymbol{j}}$,$\ {{{\boldsymbol{Y}}}_{\boldsymbol{j}}}$, sampled from a multivariate normal distribution with the mean ${\boldsymbol{\mu }}$ and the gene covariance matrix ${\boldsymbol{U}}$. This process was repeated for all locations, resulting in a 250-by-250 gene-specific expression matrix ${{{\boldsymbol{Y}}}^{\boldsymbol{G}}}$.
2. Second, we generated a 250-by-1 location-specific expression profiles vector for each gene ${\boldsymbol{g}}$, ${{{\boldsymbol{Y}}}_{{\boldsymbol{g}},}}$, sampled from a multivariate normal distribution with mean zeros and the spatial covariance ${\boldsymbol{V}}$. This process was repeated for all genes, yielding a 250-by-250 location-specific expression matrix ${{{\boldsymbol{Y}}}^{\boldsymbol{S}}}$. To evaluate each method’s performance under varying spatial dependency patterns, we also constructed spatial kernels with three levels of strength: weak, moderate, and strong by varying the bandwidth parameter ${{\bf \tau }}$ from 0.3, 0.5 to 1, following SpatialPCA [20].
3. Given ${{{\boldsymbol{Y}}}^{\boldsymbol{G}}}$ and ${{{\boldsymbol{Y}}}^{\boldsymbol{S}}}$, we simulated spatial transcriptomics data ${\boldsymbol{Y}}$ by ${\boldsymbol{Y}} = \ {{{\boldsymbol{Y}}}^{\boldsymbol{G}}} + \ {{{\boldsymbol{Y}}}^{\boldsymbol{S}}}$. We followed the same procedure described in model-based simulation to convert the simulated normalized gene expression to a count matrix for benchmarking with methods that take count data as input, such as CS-CORE, Giotto and SpaceX.

#### Simulation without gene–gene correlations

To evaluate spMOCA’s performance in scenarios when there are no gene–gene correlations, we s*et al*l off-diagonal elements in the gene covariance matrix ${\boldsymbol{U}}$ to zero, retaining only the diagonal elements of ${\boldsymbol{U}}$ to model gene variance. This scenario is crucial for assessing spMOCA’s specificity and determine whether it falsely estimates non-zero correlations when the truth are zeros. Using this modified gene covariance matrix ${\boldsymbol{U}}$, we simulated the SRT data through both simulation with spatial-gene interactive and additive dependency scenarios.

#### Co-expression estimations for simulated data

We benchmarked spMOCA against four competing methods—CSCORE, Giotto, SpaceX, and Pearson’s correlation across simulation scenarios. For CSCORE, Giotto, and SpaceX, we followed their tutorials to obtain their estimated gene–gene correlation matrices. spMOCA requires a pre-specified spatial kernel, which involves determining the bandwidth ${\boldsymbol{\tau }}$ in Equation (4). Since the true bandwidth is known in simulations, we can use it to construct a spatial kernel for a gene correlation estimation for both the interactive and the additive dependency scenarios. However, in real data application, the true bandwidths are usually unknown. Therefore, we also evaluate spMOCA’s performance when the bandwidth parameter ${{\bf \tau }}$ is unknown by estimating it directly, as described in [20]. Specifically, following a widely used approach of estimating spatial kernel bandwidth from recent studies [20, 104, 105] in spatial transcriptomics, for each gene, we applied the Silverman’s rule-of-thumb (ROT) [93] to estimate the bandwidth on the scaled gene expression data, as detailed in Supplementary Note S1. The final bandwidth parameter ${{\bf \tau }}$ is determined as the median of the bandwidth values estimated across all genes.

#### Evaluations of method performance in simulations

To evaluate the accuracy of the estimated gene–gene correlation matrix from each method, we calculated five metrics: RMSE, MAD, PCC, RV coefficients (RV), and ARI. These metrics are computed by comparing the upper triangular entries of estimated correlation matrix ${\boldsymbol{\hat{R}}}$ with those of the true correlation matrix ${\boldsymbol{R}}$. Specifically, we calculated RMSE as:


\begin{eqnarray*}
{\boldsymbol{RMSE}} = \sqrt {\frac{1}{{{{{\boldsymbol{N}}}_{{\boldsymbol{upper}}}}}}\mathop \sum \limits_{{\boldsymbol{upperi}} \not= {\boldsymbol{i^{\prime}}}} {{{\left( {{{{\boldsymbol{R}}}_{{\boldsymbol{ii^{\prime}}}}} - \widehat {{{{\boldsymbol{R}}}_{{\boldsymbol{ii^{\prime}}}}}}} \right)}}^2}}
\end{eqnarray*}

where ${{{\boldsymbol{N}}}_{{\boldsymbol{upper}}}}$ represents the number of upper triangular entries. We calculated MAD as:


\begin{eqnarray*}
{\boldsymbol{MAD}} = {\boldsymbol{media}}{{{\boldsymbol{n}}}_{{\boldsymbol{upper}}}}\left| {{{{\boldsymbol{R}}}_{{\boldsymbol{ii^{\prime}}}}} - {{{{\boldsymbol{\hat{R}}}}}_{{\boldsymbol{ii^{\prime}}}}}} \right|
\end{eqnarray*}

PCC is calculated as the correlation between the vectorized upper triangle entries of the estimates and true correlation matrices. The RV coefficient [106], which ranges from 0 to 1, measures the similarity between two matrices. The formula of RV coefficient is:


\begin{eqnarray*}
{\boldsymbol{RV}} = \frac{{{\boldsymbol{trace}}\left( {{\boldsymbol{R\hat{R}}}} \right)}}{{\sqrt {{\boldsymbol{trace}}\left( {{\boldsymbol{RR}}} \right){\boldsymbol{trace}}\left( {{\boldsymbol{\hat{R}\hat{R}}}} \right)} }}
\end{eqnarray*}

To evaluate the accuracy of detected gene modules, we calculated ARI between the detected gene modules and the true modules. The formula of ARI is provided in Supplementary Note S3. Specifically, for gene module detection, we applied the weighted correlation network analysis (WCGNA) [107] on the estimated gene–gene correlation matrix from each method with the resolution parameter *minClustersize* = 20. After obtaining the detected module labels from WCGNA, we calculate the ARI by comparing these detected labels with the original labels which were assigned during the generation of the five gene modules in the covariance matrix ${\boldsymbol{U}}$.

### Spatial transcriptomics datasets and data preprocessing

We performed three sets of gene co-expression analyses on nine published spatial transcriptomics datasets (Supplementary Table S3).

#### 10X Visium cancer data

We downloaded spatial transcriptomics data from four different cancer types—breast ductal carcinoma (BCRA), colorectal intestine cancer (CRC), lung squamous cell carcinoma (LUSC), ovarian carcinoma (OVAC)—from 10X Genomics Platform (https://www.10xgenomics.com/). All tumor tissue samples are formalin-fixed paraffin-embedded (FFPE) samples. The numbers of measured spatial locations are different, which are 2518 spatial locations in BCRA, 2660 spatial locations in COAD, 3858 spatial locations in LUSC, and 3455 spatial locations in OVAC. The numbers of measured genes are 17 943 genes in BCRA, 17 943 genes in CRC, 18 085 genes in LUSC and 17 943 genes in OVCA. To investigate the clinical implications of our cancer SRT data analysis, we identified prognostic genes using data from The Cancer Genome Atlas (TCGA, https://www.cancer.gov/ccg/research/genome-sequencing/tcga) through R Package *TCGABiolinks* [54]. Specifically, we retrieved transcriptome profiling data and associated clinical information, including survival outcomes, from TCGA-BRCA, TCGA-COAD, TCGA-LUSC, and TCGA-OV projects. Detailed descriptions of the methods used to extract prognostic genes are provided in the Real Data Analysis Workflow section.

#### 10X Visium aging mouse brain data

We downloaded 10X Visium aging mouse brain spatial transcriptomics data [35] from GEO website (accession number GSE212903, Samples ID GSM6560897, GSM6560898, and GSM6560899) [35]. These datasets were generated from brain tissues of mice aged 6 months, 18 months, and 21 months by 10X Visium platforms. The three datasets contain 2573, 2735, and 2973 spatial locations, respectively, with 32 285 genes measured in each dataset.

#### MERFISH mouse and human cortex data

We downloaded MERFISH mouse and human data [36] from an author-provided database (https://datadryad.org/dataset/doi:10.5061/dryad.x3ffbg7mw, sample ID H18.06.006.MTG.4000.expand.rep1 and mouse2.AUD_TEA_VIS.242.unexpand). The datasets include 3044 spatial locations for the human sample and 6752 spatial locations for the mouse sample. The human dataset contains 3999 genes, while the mouse dataset includes 234 genes, which were matched to their human homologs in the original expression data. Among these, 136 genes are shared between both datasets. Cell type annotations were also obtained from the database for both datasets, including excitatory neurons, inhibitory neurons, astrocytes, microglial cells, oligodendrocytes, oligodendrocyte progenitor cells, endothelial cells, and mural cells. Additionally, the mouse dataset includes vascular smooth muscle cells.

#### Normalization and feature selection

In all datasets, raw gene expression counts were first normalized by library size factor to mitigate technical biases across different spots, and then log-transformed to convert the counts to a continuous scale. Following the SpaceX methodology, we focused on the GCN within SVGs. For SVGs selection, we applied SPARK [92] to identify SVGs with adjusted *P*-value >0.05. For the 10X Visium cancer datasets, we selected 6919, 9884, 11 074, and 10 169 SVGs for BRCA, CRC, LUSC, and OVCA dataset, respectively. For 10X Visium aging mouse brain datasets across three time points, we selected 1917 significant SVGs among those age-related genes provided by the original paper [35]. For MERFISH cortex datasets, due to the limited number of genes measured, we focused on the overlapping genes between human and mouse datasets without further SVG selection, resulting in 136 genes.

### Real data analysis workflow

#### Gene co-expression estimation

We applied four methods to infer gene co-expression in real data: spMOCA, CSCORE, Giotto, and Pearson’s correlation. SpaceX was omitted due to its high computational cost on 10X Visium and MERFISH datasets (e.g. running SpaceX on the 10X Visium BRCA cancer data required 2 days and 32 GB of memory and failed to complete under default parameters). For CSCORE and Giotto, we followed their tutorials and default settings to obtain their gene–gene correlation estimates. In all real datasets, we constructed the spatial covariance matrix for spMOCA following the procedure described in the section “Co-expression estimations for simulated data.”

#### Gene module detection, hub gene identification, and module score calculation

Gene modules were identified using the WCGNA [107] framework, a widely used algorithm for detecting gene modules from a gene co-expression matrix. Specifically, WCGNA takes the gene–gene correlation matrix from each method as input, converts it into a Topological Overlap Matrix (TOM), and then applies the Dynamics Tree Cut algorithm [108] to identify gene modules. To ensure the consistency in the number of gene modules across all methods we compared, we tuned the resolution parameter, *minClustersize*, for each gene–gene correlation matrix input. Specifically, for each co-expression estimation method, we detected 7 modules for the 10X Visium cancer data, 5 modules for the 10X Visium aging mouse brain data, and 5 modules for the MERFISH human and mouse cortex data respectively.

To identify hub genes, which are defined as genes highly correlated with others within a GCN, we calculated each gene’s network degree. Specifically, we first transformed the co-expression matrix into a similarity matrix ${\boldsymbol{A}}$ by (co-expression + 1)/2. Then for a given gene ${\boldsymbol{i}}$, it’s co-expression network degree was calculated by the sum of the edges connecting gene ${\boldsymbol{i}}$ to all other genes in a set ${\boldsymbol{L}}$, i.e. $\mathop \sum \limits_{{\boldsymbol{j}}\in {{\bf L}},{\boldsymbol{j}} \not= {\boldsymbol{i}}} {{{\boldsymbol{A}}}_{{\boldsymbol{ij}}}}$ where ${{{\boldsymbol{A}}}_{{\boldsymbol{ij}}}}$ is the entry of ${\boldsymbol{A}}$ [109]. Based on the gene’s degree, we define global hub genes as those with top degrees when ${\boldsymbol{L}}$ is the set of all genes. we also identify module-specific hub genes as those with the top degrees when ${\boldsymbol{L}}$ is restricted to the set of genes within the same module.

For the 10X Visium cancer data and the 10X Visium aging mouse brain data, we selected the top 5% of hub genes—defined as genes with the highest within-module connectivity—as representatives of each module. Top 5% is a commonly used threshold for hub gene selection [110, 111]. In addition, for 10X Visium aging mouse brain data, we also defined top 5% degree-variable genes with the largest variation of their global degree across three time points. Detailed descriptions of identifying the top degree-variable genes are provided in the section “Top degree-variable genes in mouse aging brain data.” For the MERFISH data, we did not select hub genes due to the limited number of genes measured.

We then calculated module scores to summarize the expression levels of gene modules across spatial locations. In the 10X Visium cancer data, the module score was computed as the average normalized expression of its hub genes at each spatial location. For the MERFISH data, module scores were calculated as the average normalized expression of all genes within a module for each cell, as hub gene selection was not applied due to the smaller number of genes measured. This approach provides a concise measure of module activity, enabling the assessment of collective expression impact of each module across spatial locations. Additionally, in the MERFISH data, we calculated cell-type-level module scores by averaging the module scores of all cells within the same cell type. This enabled us to identify the gene modules that most strongly characterize specific cell types based on their co-expression patterns. For 10X Visium aging mouse brain data, we focused on analyzing changes in genes’ network degrees and gene co-expression relationships.

#### Gene module evaluation

For all real datasets, we evaluated gene modules using three metrics: between-module variation, Davies–Bouldin index (DB-index), and dissimilarity of module-associated pathways. Between-module variation and the DB-index were used to assess between-module dissimilarity and within-module cohesion based on gene expression patterns. Notably, the DB-index has been previously employed to evaluate the effectiveness of different gene co-expression measures in guiding module detection [112]. In the 10X Visium cancer data and the 10X Visium aging mouse brain, these metrics were calculated using hub genes as representatives of each module. For the MERFISH data, we focused on the shared genes between the mouse and human datasets to assess how this set of genes is clustered into different modules in the two species.

We first calculated the between-module variation following the formula in [113], which calculated overall distances between module centroids and global centroids to measure the separability of modules. To measure global centroids, we calculated the average normalized expression level of all candidate genes in each dataset. Similarly, for each module, we calculated its centroid by averaging the normalized expression levels of the candidate genes within the module. The between-module variation for each dataset was computed as:


\begin{eqnarray*}
&&{\boldsymbol{BetweenModule}} = \ \mathop \sum \limits_{\boldsymbol{j}} {{{\boldsymbol{n}}}_{{\boldsymbol{gene}},{\boldsymbol{j}}}}*{\boldsymbol{mean}}\\&& \left( {{\boldsymbol{global}}\ {\boldsymbol{centroid}} - {\boldsymbol{module}}\ {\boldsymbol{j}}\ {\boldsymbol{centroid}}} \right)
\end{eqnarray*}

Where ${\boldsymbol{j}}\ $denotes the index of a gene module, ${{{\boldsymbol{n}}}_{{\boldsymbol{gene}},{\boldsymbol{j}}}}$ represents the number of candidate genes in module ${\boldsymbol{j}}$, and ${\boldsymbol{mean}}()$ calculates the mean of a vector.

We then calculated the DB-index, which is another known clustering evaluation metric for assessing averaged between-cluster similarity [113]. DB-index was .We used *index.DB()* from R package *clusterSIM* to calculate the DB-index based on standard deviation of the Euclidean distance between candidate genes in module ${\boldsymbol{j}}$ to the module centroid. A lower DB-index indicates less similarity between clusters, meaning the modules are more distinct and well-defined.

Moreover, we examined functional differences between gene modules by assessing the dissimilarity in their associated biological pathways. To quantify this, we constructed a binary vector ${\boldsymbol{Q}}$ for each gene module given a pathway set ${\boldsymbol{h}}$, where ${{{\boldsymbol{Q}}}_{\boldsymbol{h}}} = 1$ indicates that pathway ${\boldsymbol{h}}$ significantly associated with the gene module and ${{{\boldsymbol{Q}}}_{\boldsymbol{h}}} = 0$ otherwise. Details of identifying associated pathways are provide in the section “Gene set analysis.” For any two gene modules with binary vectors ${{{\boldsymbol{Q}}}_1}$ and ${{{\boldsymbol{Q}}}_2}$, we calculated the Hamming distance by:


\begin{eqnarray*}
(\mathop \sum \limits_{{\boldsymbol{h}}\in {\boldsymbol{H}}} {\boldsymbol{I}}\left( {{{{\boldsymbol{Q}}}_{1,{\boldsymbol{h}}}}\not= {\mathrm{\ }}{{{\boldsymbol{Q}}}_{2,{\boldsymbol{h}}}}} \right))/\left| {\boldsymbol{h}} \right|
\end{eqnarray*}

Where ${\boldsymbol{I}}()$ is the indicator function and $| {\boldsymbol{h}} |$ is the size of the pathway set ${\boldsymbol{h}}$. According to the definition, a larger hamming distance indicates a smaller overlap of associated pathways between two gene modules, which suggests greater functional divergence or distinct biological roles of the modules.

#### Gene set analysis

For each gene module, we conducted gene set analysis to identify its significantly associated biological pathways. We obtained a comprehensive list of human and mouse gene sets from MsigDB (https://www.gsea-msigdb.org/gsea/index.jsp) [44, 114, 115], totaling 32 880 and 32 872 gene sets, respectively. To determine which gene sets are significantly associated with each gene module, we first constructed a two-way contingency table by categorizing genes based on their presence in the gene module and the corresponding gene set. We then computed the statistical significance of enrichment using Fisher’s exact test, with a *P*-value threshold of 0.05. Consistent with the section “Gene module evaluation,” we performed the test on module hub genes for 10X Visium cancer data and genes shared across species for MERFISH data. For 10X Visium mouse aging brain data, we performed the test on module hub genes and the top degree variable genes.

To evaluate whether our identified gene modules are biologically relevant to the tissue type, we focused on a subset of the pathways based on their category information provided by MsigDB. For 10X Visium cancer data, due to the lack of references which summarize tumor-context-specific, we followed [25, 116] focused on benchmarking gene modules’ separability in Gene Ontology pathways, and then further explored cross-tumor shared pathways in Oncogenic gene sets, KEGG pathways, BIOCARAT pathways, REACTOME pathways, WIKIPATHWAYS pathways, and Cancer Modules, resulting 18 850 gene sets in total. For 10X Visium mouse aging brain data, we focused the analysis on the pathways related to aging and neurological diseases related pathways (e.g. Alzheimer’s disease, stroke, and cell aging pathway), resulting 672 gene sets in total. For MERFISH data, we focused the analysis on pathways related to astrocytes, microglia, oligodendrocytes, endothelial, excitatory and inhibitory neuron, and vascular smooth muscle cell types, obtained by using annotated cell types as keywords to search in MsigDB database, resulting 405 gene sets.

#### Cell-type deconvolution

In the 10X Visium cancer data analysis, we applied CARD [17] to estimate cell type proportions for each cancer data. CARD takes a scRNA-seq data as a reference and performs spatially informed cell-type deconvolution for SRT data. Specifically, we obtained the scRNA-seq reference data for BRCA [117] from Gene Expression Omnibus (GEO) repository (accession number: GSE176078), CRC [118] from GSE144735, LUSC from GSE131907 [53], and OVCA [49] from https://data.mendeley.com/datasets/rc47y6m9mp/1. Details of the scRNA-seq reference data are in Supplementary Table S3. The reference datasets comprise 29 cell types for BRCA, 40 cell types for CRC, 49 cell types for LUSC, and 7 cell types for OVCA (Supplementary Figs S16–S19). We performed cell type decomposition for all cell types in the datasets and then compared tumor- and immune-related module scores with the distributions of specific cell types. For tumor-related cell types, we examined cancer cell subtypes (Cancer_subtype_SC) in BRCA, CMS1-4 cells in CRC, tumor cells with different transcriptional states (tS1-3), and Malignant cells in LUAD and cancer cells in OVCA. For immune cell types, we examined specific subsets, including memory B cells and T cells (e.g. CD8 + T cells) and macrophages in BRCA; CD19 + CD20 + B cells and CD8 + T cells in CRC; CD4 naive T cells, dendritic cells (e.g. CDC1c DCs), and NK cells in LUSC; and myeloid cells in OVCA.

#### Identifying prognostic genes from TCGA data

To identify prognostic genes for each cancer type, we followed the *TCGABiolinks* tutorial [54] (https://bioconductor.org/packages/release/bioc/vignettes/TCGAbiolinks/inst/doc/casestudy.html) to perform survival analysis using RNA expression data and associated clinical information from TCGA cohorts. For each cancer type, we downloaded transcriptome profiling data using the functions *GDCquery()* and *GDCprepare()*, and retrieved the associated clinical data using *GDCquery_clinic()*. The data were then preprocessed following the tutorial instruction, with a sample outlier removing, a GC context normalization and a transcripts filtering, and then we utilized the normalized data and clinical information to conduct a univariate Kaplan–Meier survival analysis. Specifically, the samples were divided into two groups along the 33rd and 66th percentiles of the mean expression of a gene in the normalized data. A survival analysis was then performed between the groups of samples with high expression and the groups with low expression, to assess the difference between two groups’ survival outcomes. All parameters for the function were set to their default values, as described in the tutorial. Genes with a *P*-value <0.05 from the K-M analysis were then performed a cox-regression analysis. The genes with cox *P*-value FDR < 0.05 were identified as prognostic genes. We then overlapped the prognostic markers with the hub genes of the tumorigenesis-related modules and the immune-related modules to evaluate the clinical relevance of these modules.

#### Top degree-variable genes in mouse aging brain data

Using the 10X Visium mouse aging brain dataset, we analyzed the global network degree of each age-related SVGs within each method’s GCN across three time points. Specifically, we first calculated each gene’s network degree by summing the connections to all other age-related SVGs. The definition of gene’s network degree was provided in the section “Gene module detection, hub gene identification and module score calculation.” To ensure comparability, the network degrees were min-max normalized within each GCN at each time point. Next, for each co-expression method, we computed the sum of squared of normalized network degrees for each gene across the three time points by:


\begin{eqnarray*}
&& {{\left( {{\boldsymbol{Degre}}{{{\boldsymbol{e}}}_{6{\boldsymbol{month}}}} - \overline {{\boldsymbol{Degree}}} } \right)}^2} + {{\left( {{\boldsymbol{Degre}}{{{\boldsymbol{e}}}_{18{\boldsymbol{month}}}} - \overline {{\boldsymbol{Degree}}} } \right)}^2} + \\&& {{\left( {{\boldsymbol{Degre}}{{{\boldsymbol{e}}}_{21{\boldsymbol{month}}}} - \overline {{\boldsymbol{Degree}}} } \right)}^2}
\end{eqnarray*}

where $\overline {{\boldsymbol{Degree}}} $ is the mean normalized degrees across the three time points. Genes with the top 5% highest sum of squared were identified as the most degree-variable genes, which resulted in 95 top degree-variable genes for each co-expression method. Finally, we assessed the associations between these top degree-variable genes and aging-related pathways by performing the statistical tests described in the section “Gene set analysis.”

## Supplementary Material

gkaf1264_Supplemental_File

## Data Availability

We analyzed the following publicly available datasets: (1) 10X Visium cancer data (https://www.10xgenomics.com/datasets/human-breast-cancer-ductal-carcinoma-in-situ-invasive-carcinoma-ffpe-1-standard-1-3-0; https://www.10xgenomics.com/datasets/human-intestine-cancer-1-standard; https://www.10xgenomics.com/datasets/human-lung-cancer-ffpe-2-standard; https://www.10xgenomics.com/datasets/human-ovarian-cancer-1-standard); (2) 10X Visium aging mouse brain data (GEO accession no.: GSE212903); (3) MERFISH mouse and human cortex data (https://datadryad.org/dataset/doi:10.5061/dryad.x3ffbg7mw).
