# Supplementary material for: A spatially informed matrix normal model for gene co-expression analysis in spatial transcriptomics studies
Source: Nucleic Acids Res. 2025 Dec 10;53(22):gkaf1264. doi: 10.1093/nar/gkaf1264 (PMC12693644; doi:10.1093/nar/gkaf1264)
Supplement: gkaf1264_Supplemental_File [file gkaf1264_supplemental_file.pdf]

Supplemental Information

A Spatially Informed Matrix Normal Model for Gene Co-expression Analysis in Spatial Transcriptomics Studies

Chichun Tan[1], Ying Ma[#,1, 2]

1. Department of Biostatistics, Brown University, Providence, RI 02903 USA

2. Center for Computational Molecular Biology, Brown University, Providence, RI 02903 USA

#: correspondence to YM (ying_ma@brown.edu)

Supplementary Figures

Figure S1 The comparison of gene level means and standard deviations between the real Breast Cancer (BC) SRT data and simulated data under the scenario of gene-spatial interactive dependency. **(A)** The mean gene expression levels in the simulated data versus the real BC SRT data. **(B)** The standard deviation of gene expression in the simulated data versus the real BC SRT data. Each point in the scatterplots represents a specific gene. *R*: R-squared statistics; *p*: p-value for the slope estimation.

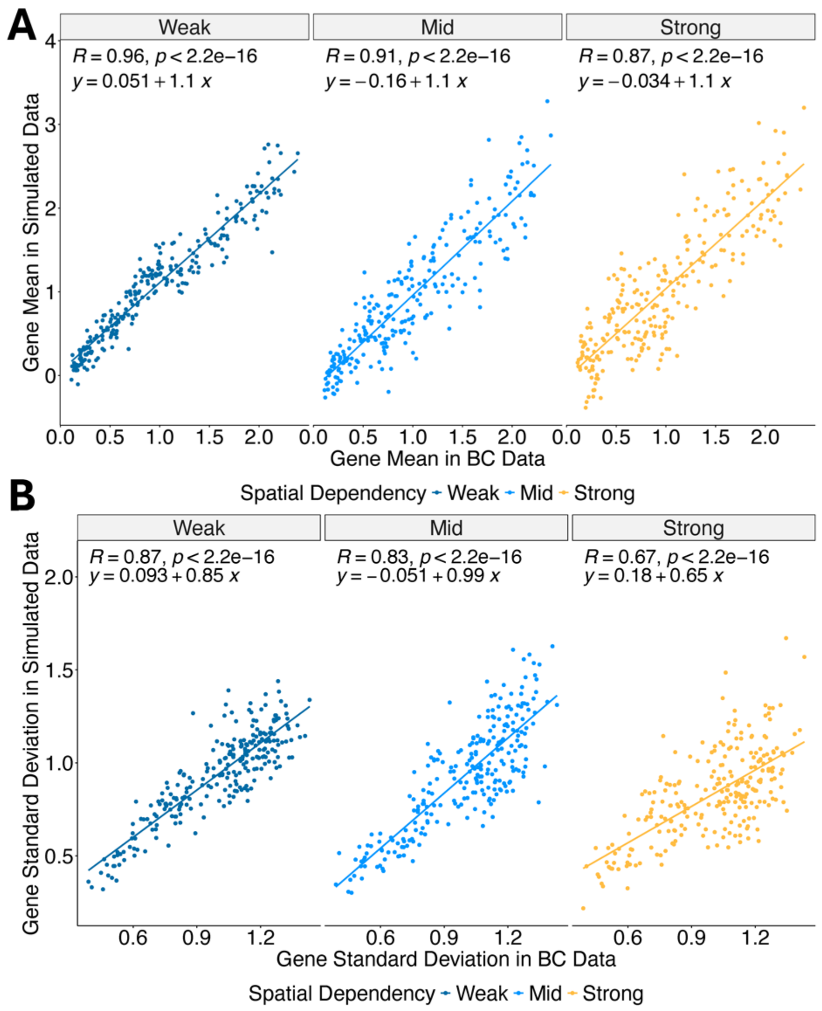

Figure S2 The comparison of gene level means and standard deviations between the real Breast Cancer (BC) SRT data and simulated data under the scenario of gene-spatial additive dependency. **(A)** The mean gene expression levels in the simulated data versus the real BC SRT data. **(B)** The standard deviation of gene expression in the simulated data versus the real BC SRT data. Each point in the scatter plots represents one gene. *R*: R-squared statistics; *p*: p-value for the slope estimation.

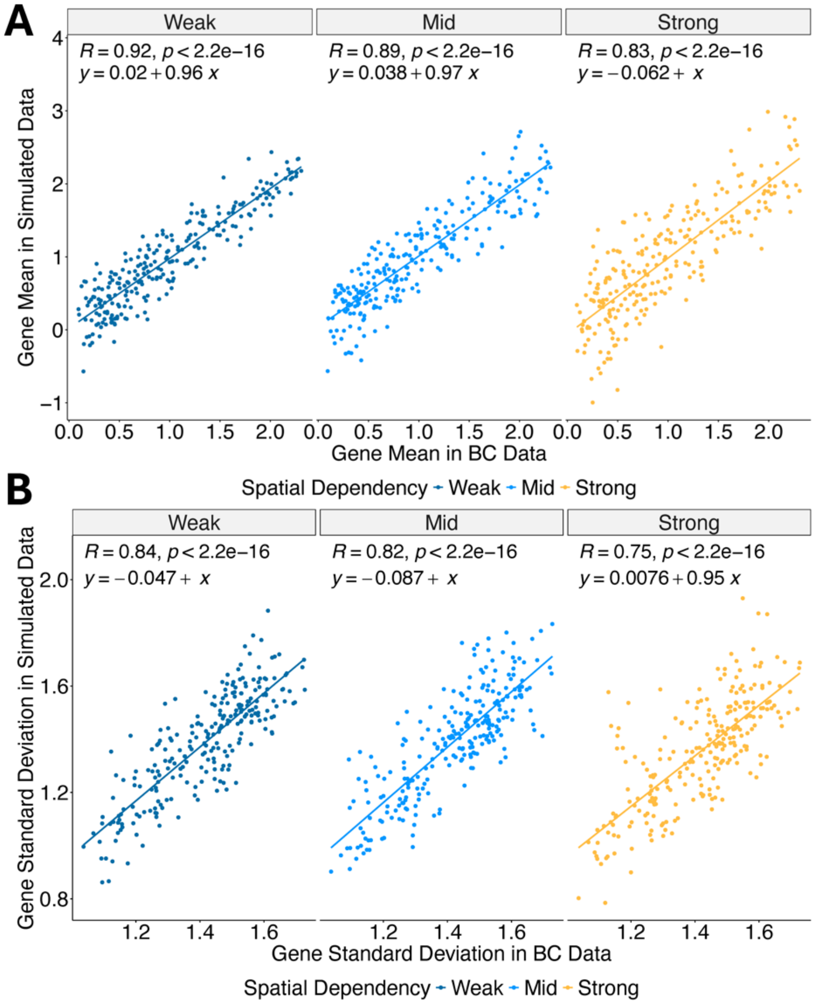

Figure S3 The evaluation of co-expression estimations in the simulation with spatial-gene interactive dependencies with estimated spatial kernel bandwidths. (A) Evaluation of accuracy of the gene co-expression estimations. The evaluation metrices from left to right are Pearson's correlation coefficient (PCC), RV coefficients (RV), Root Mean Squared Error (RMSE) and Median Absolute Deviation (MAD). (**B**) Adjusted Rand index (ARI) of the inferred gene modules compared to the true gene modules. (**C**) Heatmaps of the gene-gene correlation estimations. (**D**) The estimated values of gene co-expression estimations when there is no true gene co-expression.

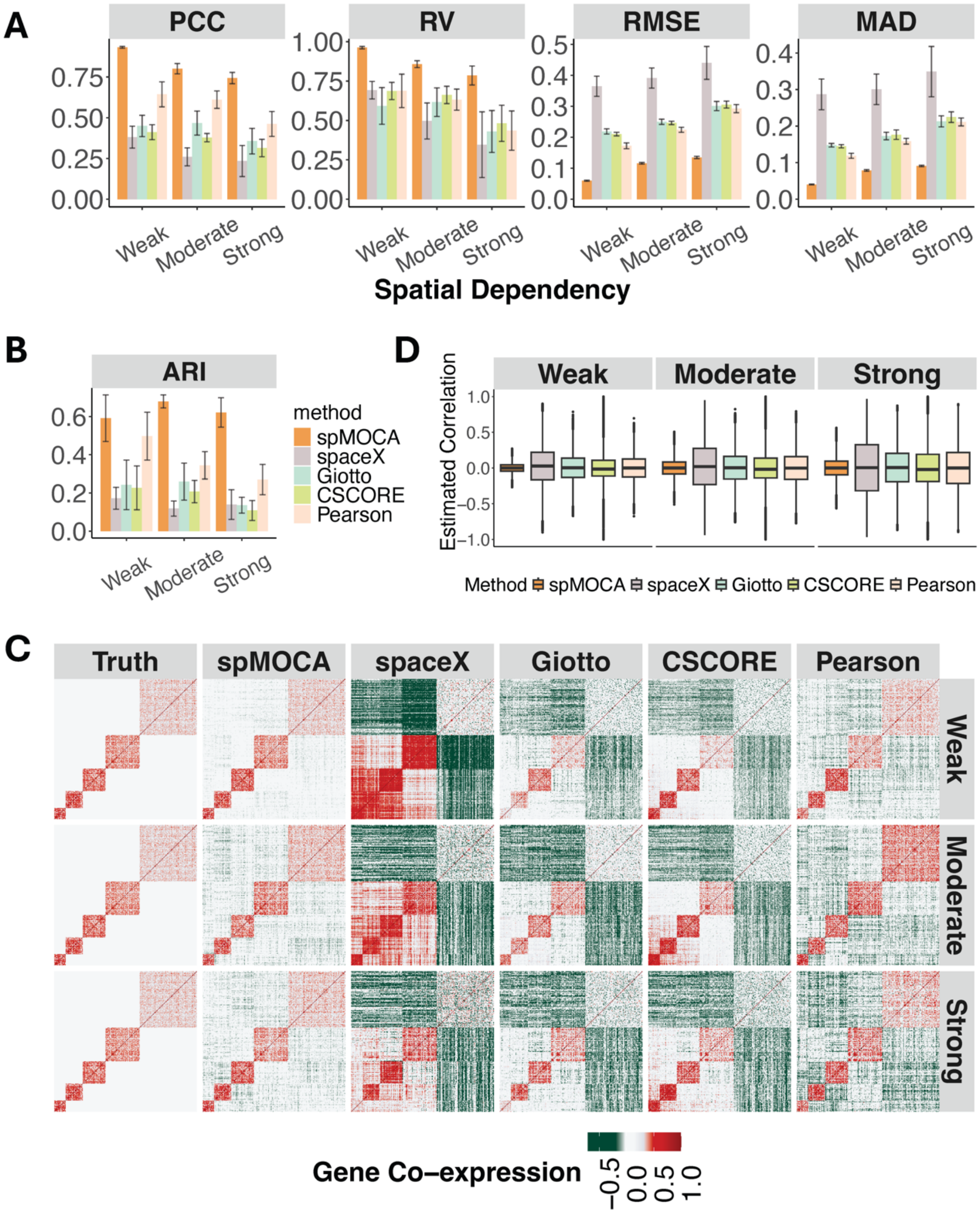

A
PCC
RV
RMSE
MAD
Weak
Moderate
Strong
Spatial Dependency
B
ARI
method
spMOCA
spaceX
Giotto
CSCORE
Pearson
D
Weak
Moderate
Strong
Estimated Correlation
Method spMOCA spaceX Giotto CSCORE Pearson
C
Truth
spMOCA
spaceX
Giotto
CSCORE
Pearson
Weak
Moderate
Strong
Gene Co–expression

Figure S4 The evaluation of co-expression estimations in the simulation with spatial-gene additive dependencies with estimated spatial kernel bandwidths. (A) Evaluation of accuracy of the gene-gene correlation estimations. The evaluation metrices from left to right are Pearson's correlation coefficient (PCC), RV coefficients (RV), Root Mean Squared Error (RMSE) and Median Absolute Deviation (MAD). (**B**) Adjusted Rand index (ARI) of the inferred gene modules compared to the true gene modules. (**C**) The estimated values of gene-gene correlation estimations when there is no true gene co-expression. (**D**) The estimated values of gene co-expression estimations when there is no true gene co-expression.

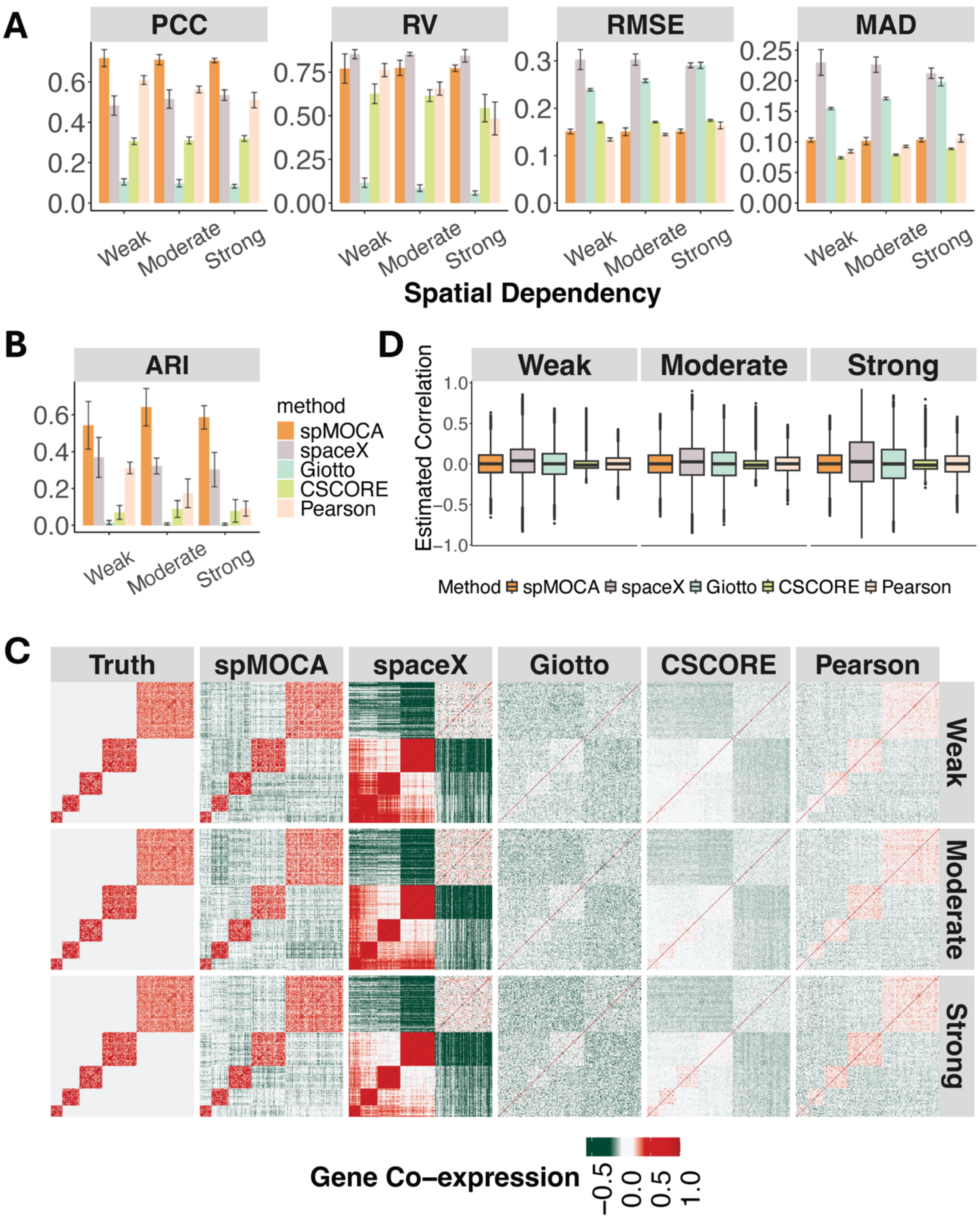
A
PCC
RV
RMSE
MAD
Weak
Moderate
Strong
Spatial Dependency
B
ARI
method
spMOCA
spaceX
Giotto
CSCORE
Pearson
D
Estimated Correlation
Method
C
Truth
Gene Co−expression

Figure S5 Recovery of tumor-specific transcription factor (TF)–target gene (TG) interactions by gene co-expression networks across 10x Visium cancer data. The number of recovered TF-TG pairs for each TF in the **(A)** BRCA, **(B)** CRC, **(C)** LUSC and **(D)** OVCA.

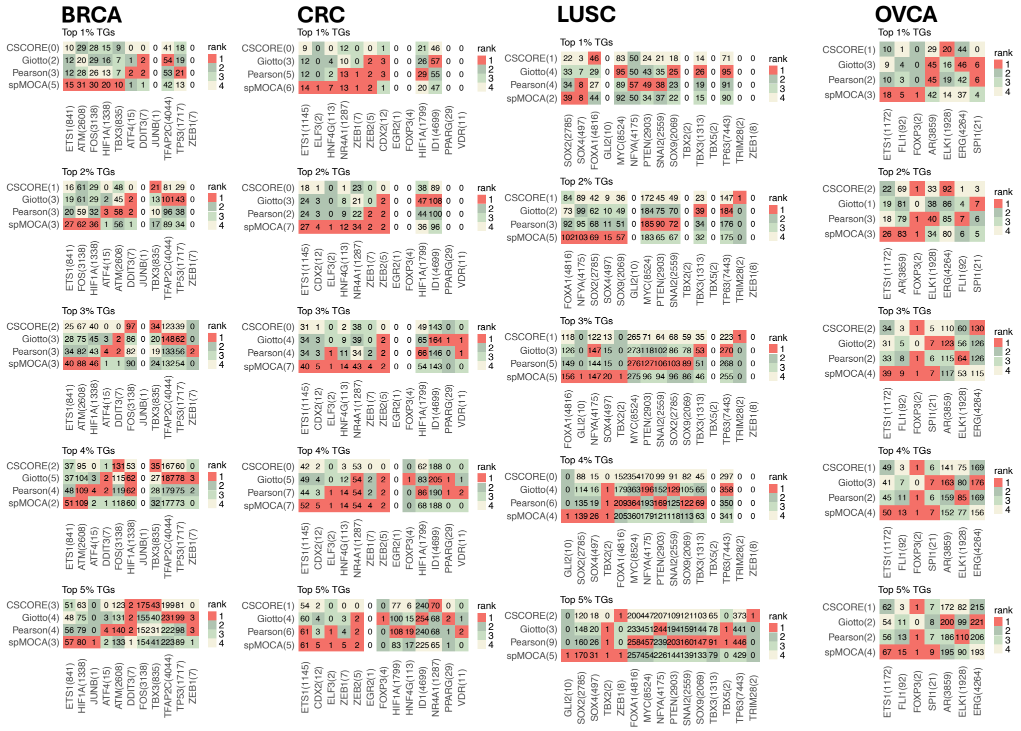

Figure S6 Gene co-expression networks in 10x Visium cancer data generated by spMOCA. Figures showed what genes exist in each gene module detected from spMOCA' GCN for each tumor data**.** Genes displayed in the figures are the Top 1% hub genes in each module. Here, each module is represented by a distinct color, with all genes within a module sharing the same color: light cyan for module 1, dark gray for module 2, light orange for module 3, light violet for module 4, light red-orange for module 5, light green for module 6 and dark blue-violet for module 7.

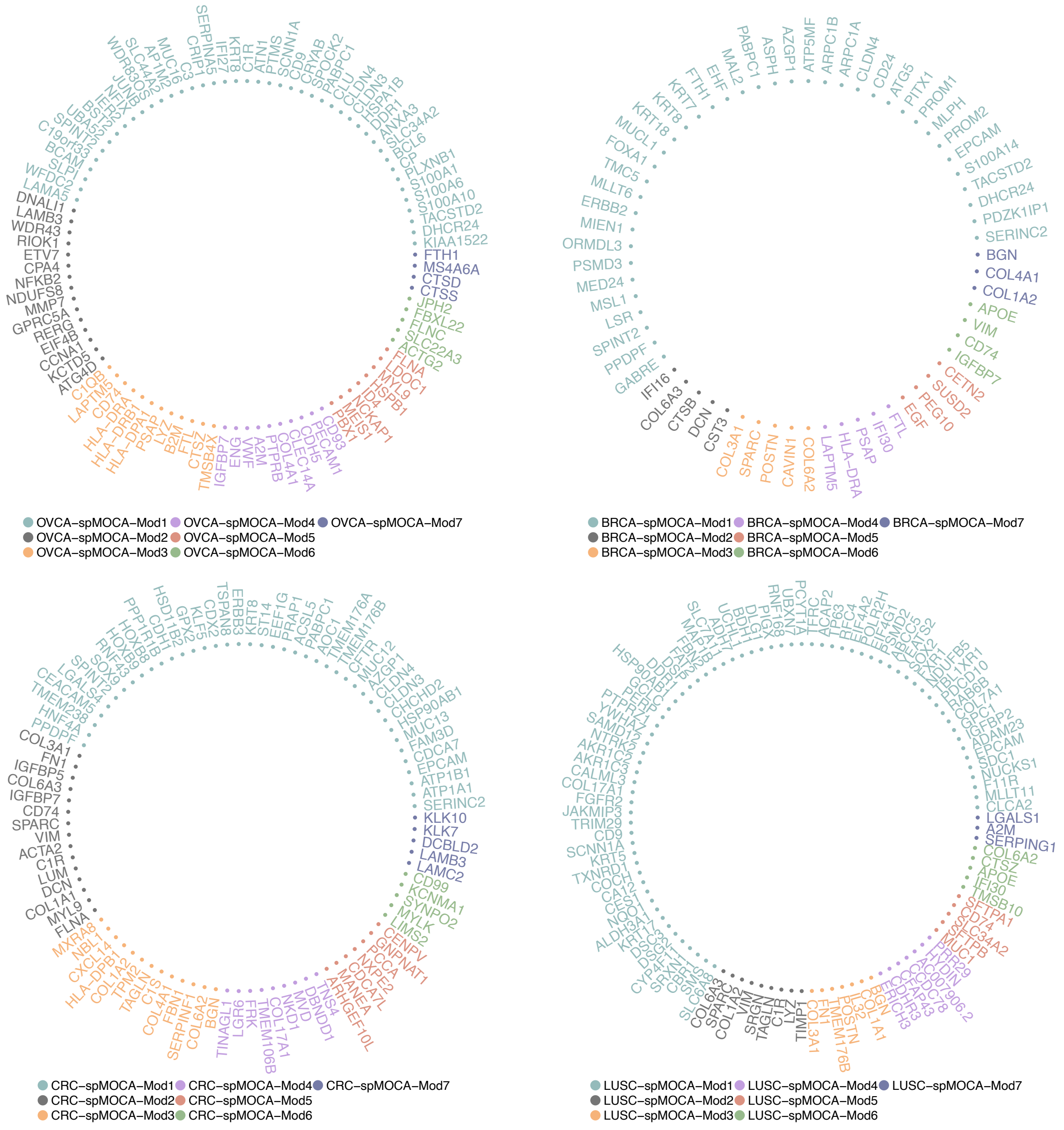

Figure S7 Gene co-expression networks in 10x Visium cancer data generated by CS-CORE. Figures showed what genes exist in each gene module detected from CS-CORE' GCN for each tumor data**.** Genes displayed in the figures are the Top 1% hub genes in each module. Here, each module is represented by a distinct color, with all genes within a module sharing the same color: light cyan for module 1, dark gray for module 2, light orange for module 3, light violet for module 4, light red-orange for module 5, light green for module 6 and dark blue-violet for module 7.

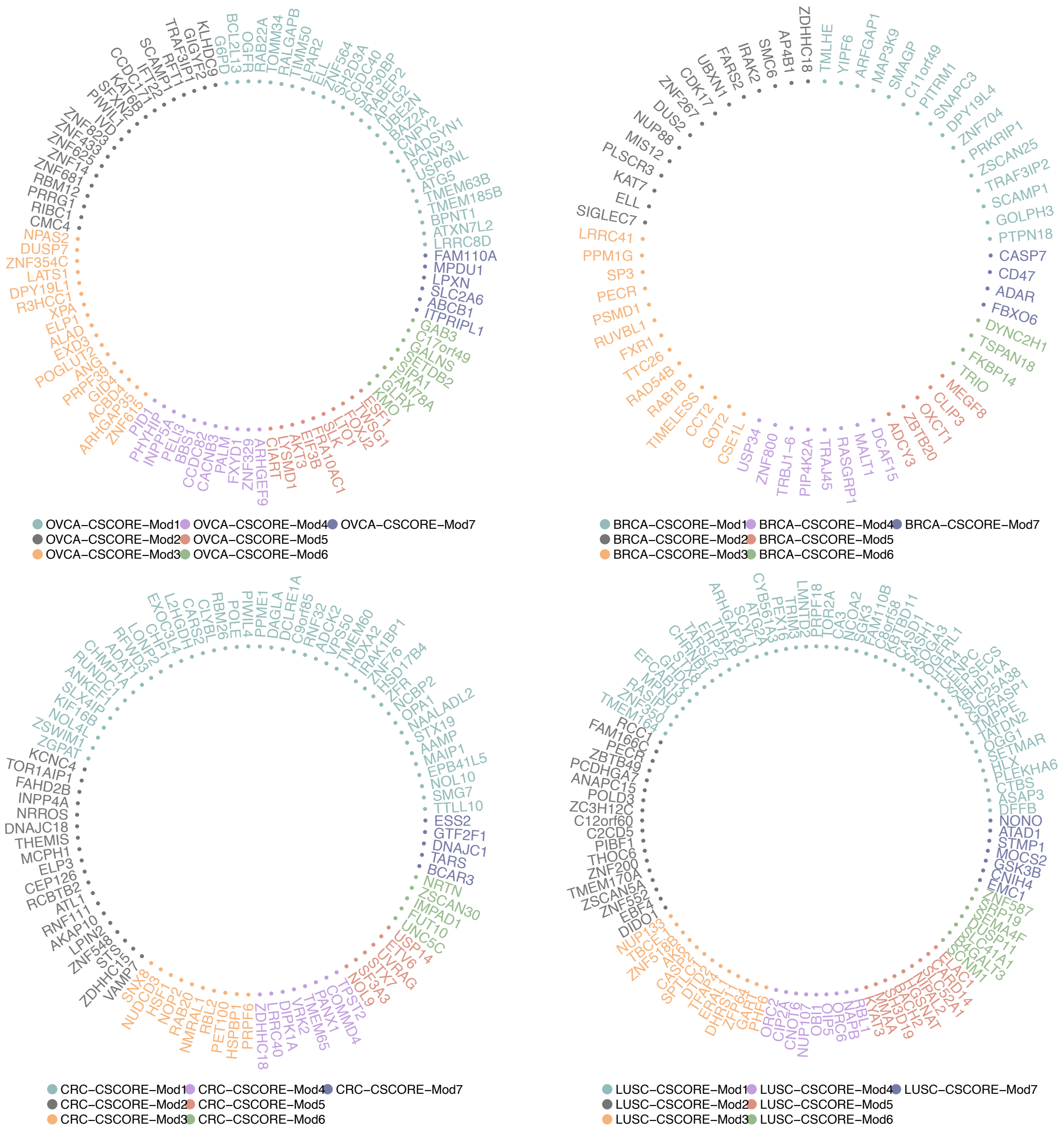

Figure S8 Gene co-expression networks in 10x Visium cancer data generated by Giotto. Figures showed what genes exist in each gene module detected from Giotto' GCN for each tumor data**.** Genes displayed in the figures are the Top 1% hub genes in each module. Here, each module is represented by a distinct color, with all genes within a module sharing the same color: light cyan for module 1, dark gray for module 2, light orange for module 3, light violet for module 4, light red-orange for module 5, light green for module 6 and dark blue-violet for module 7.

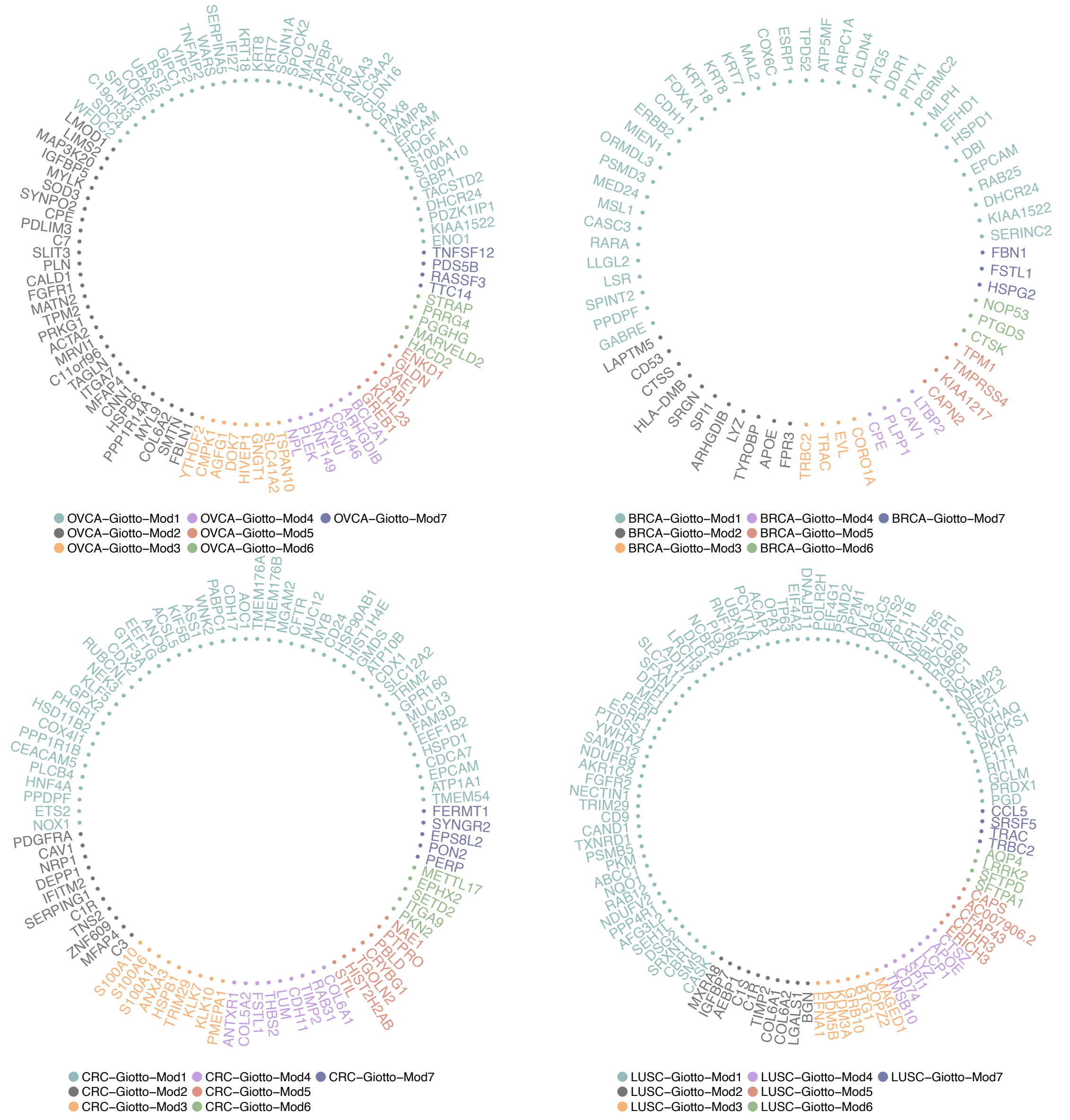

Figure S9 Gene co-expression networks in 10x Visium cancer data generated by Pearson's Correlation. Figures showed what genes exist in each gene module detected from Pearson' GCN for each tumor data**.** Genes displayed in the figures are the Top 1% hub genes in each module. Here, each module is represented by a distinct color, with all genes within a module sharing the same color: light cyan for module 1, dark gray for module 2, light orange for module 3, light violet for module 4, light red-orange for module 5, light green for module 6 and dark blue-violet for module 7.

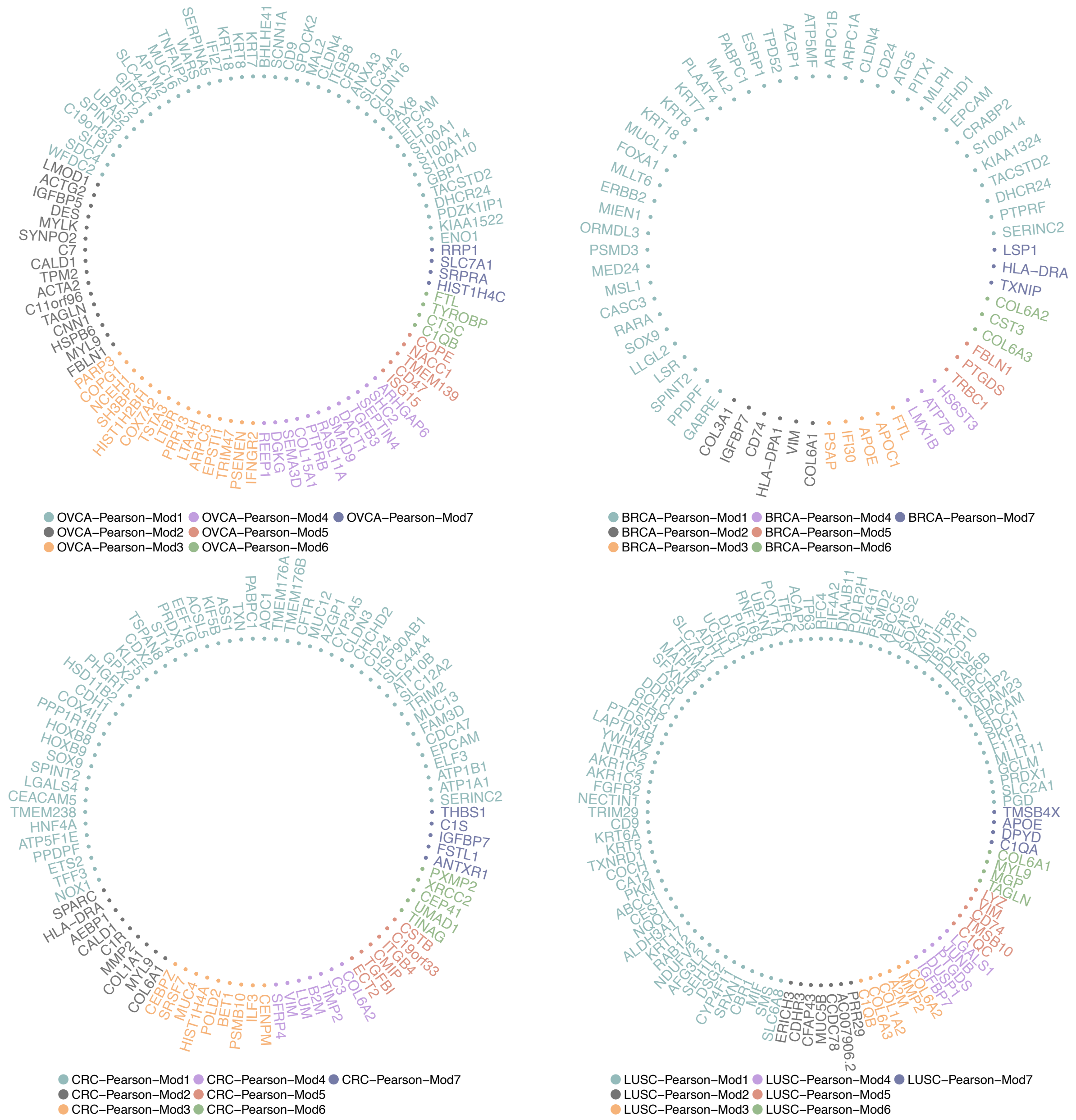

Figure S10 The between-module variance and Davies-Bouldin's index of gene modules detected by each method in 10x Visium tumor data. Here, a high between-module variation represents that gene modules have large separation in their expression patterns, and a low Davies-Bouldin's index represents that gene modules have low similarity.

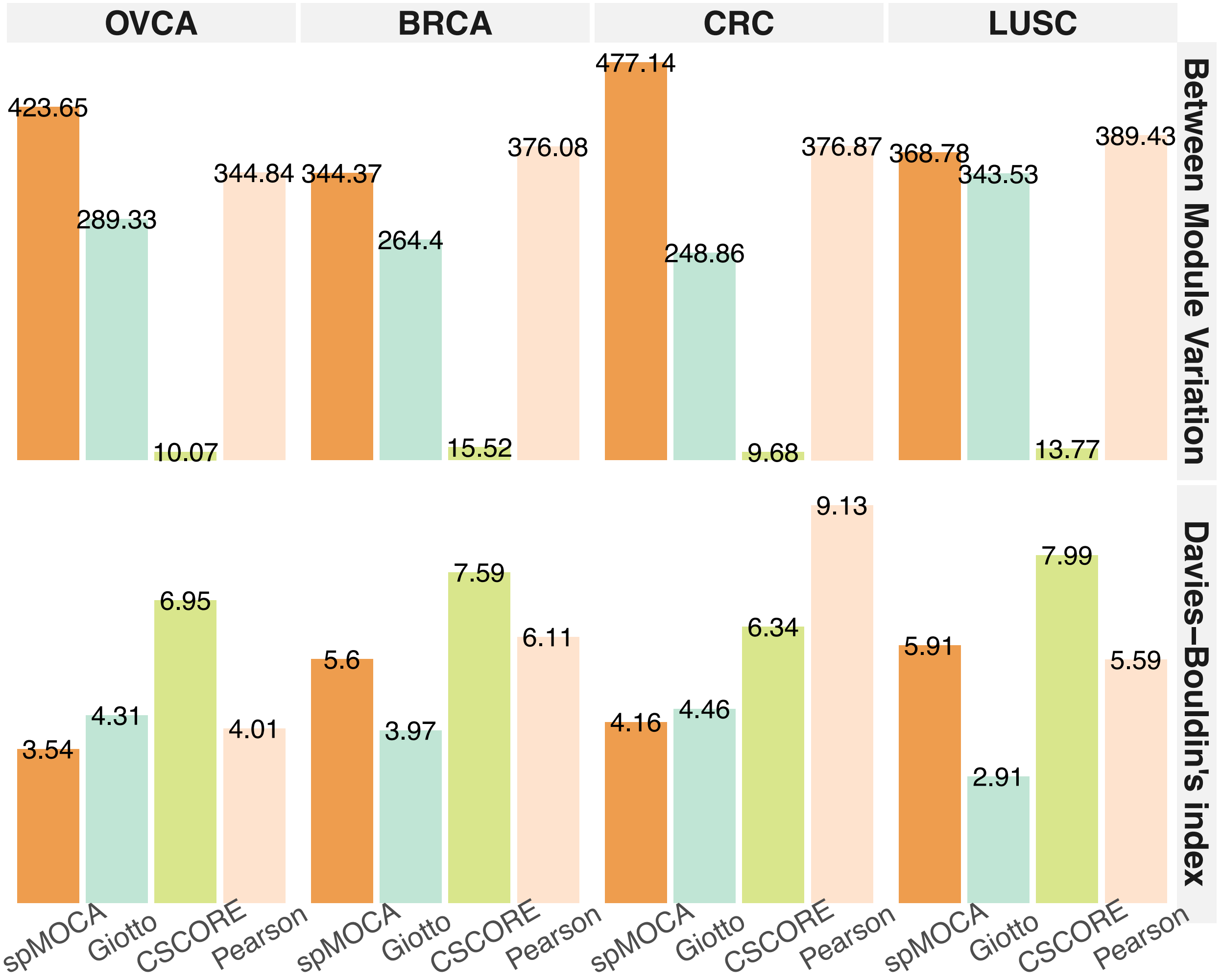

Figure S11 The dissimilarity of gene modules in Gene Ontology pathways in 10x Visium tumor data. The boxplots show modules' hamming distance based on their associated pathways**.** The pathways are the canonical pathways gene sets derived from Gene Ontology category. P-values are calculated through two-sided Wilcoxon rank sum test. *: p-value < 0.05; **: p-value < 0.01; ***: p-value < 0.001; ****: p-value < 10^{-4}

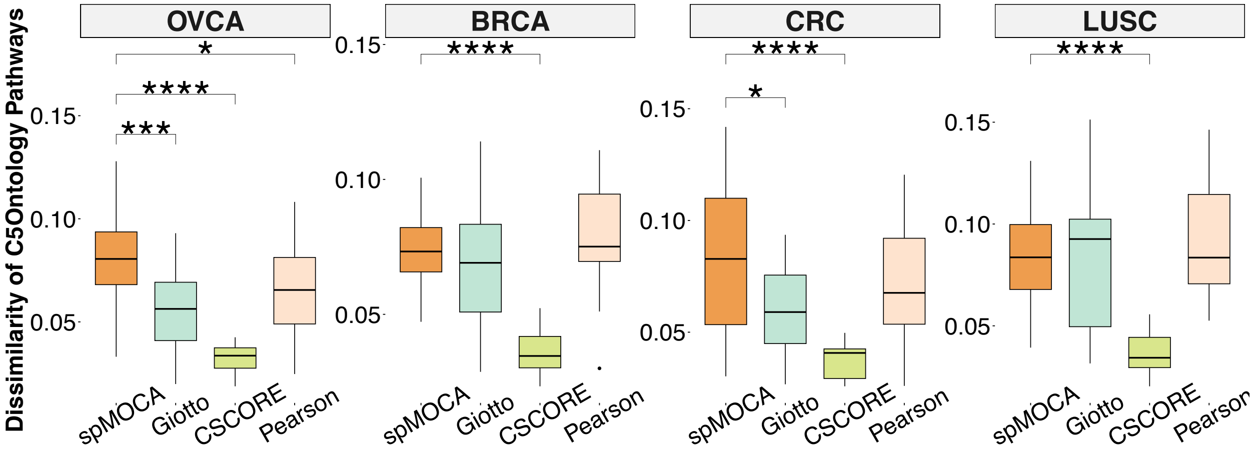

Figure S12 The number of spMOCA modules' significant associated pathways in 10x Visium tumor data. The diagonal entries represent the number of significant associated pathway for each module. The off-diagonal entries represent the number of sharing significant associated pathways between two modules. The pathways gene sets include the sets from BIOCARAT, WIKIPATHWAYS, KEGG and REACTOME database, and C5 Gene Ontology as well as C6 Oncogenic Signature Gene from GSEA database (https://www.gsea-msigdb.org/gsea/msigdb/index.jsp).

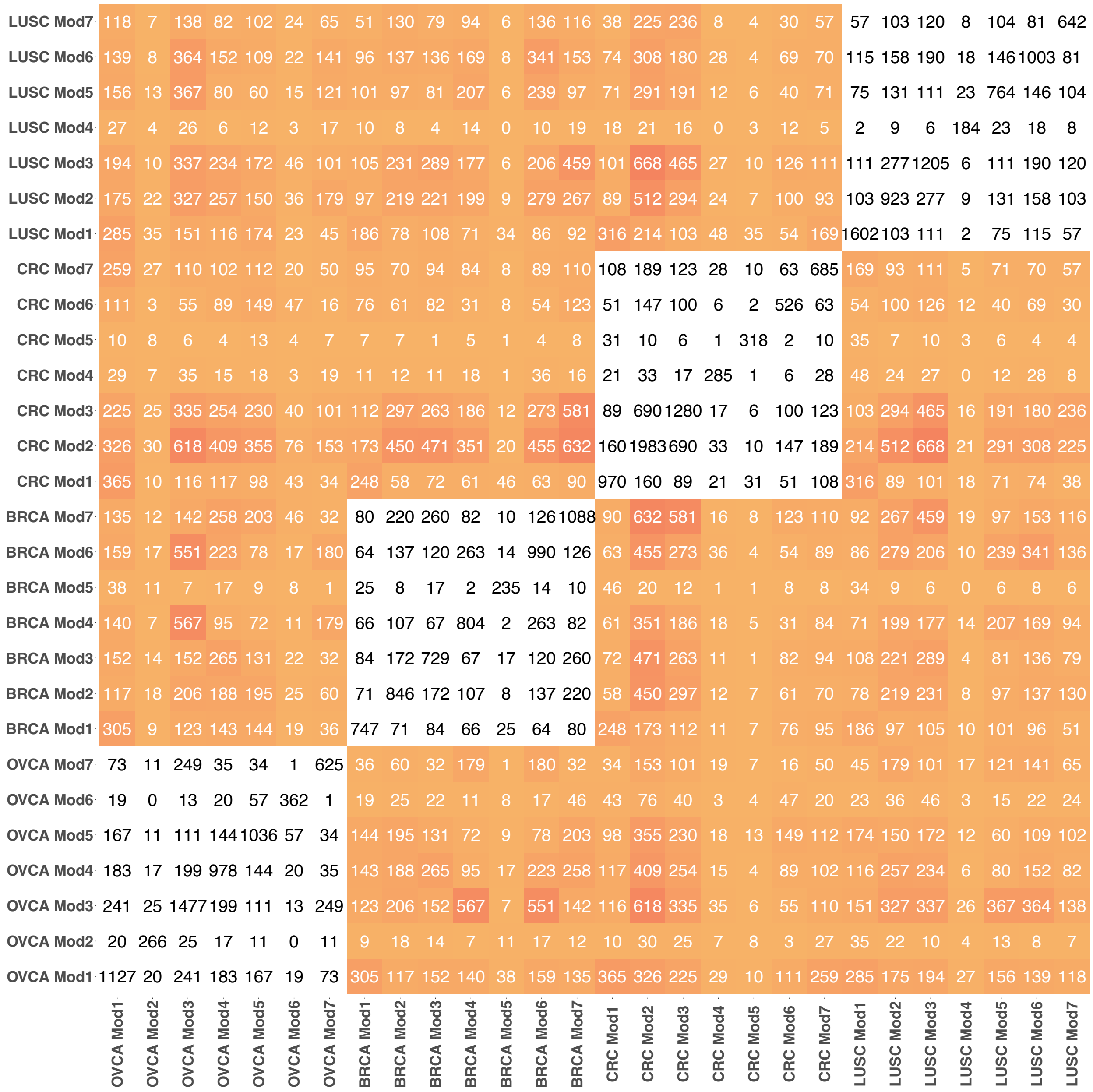

Figure S13 Pairwise counts of shared pathways across all spMOCA's immune-related modules. This figure is highlighting one of the two group of modules with high degree of pathway overlap we identified from Figure S12, which are defined as the immune-related modules. Similar to Figure 3D, the number in each block represents the number of sharing gene sets between two modules.

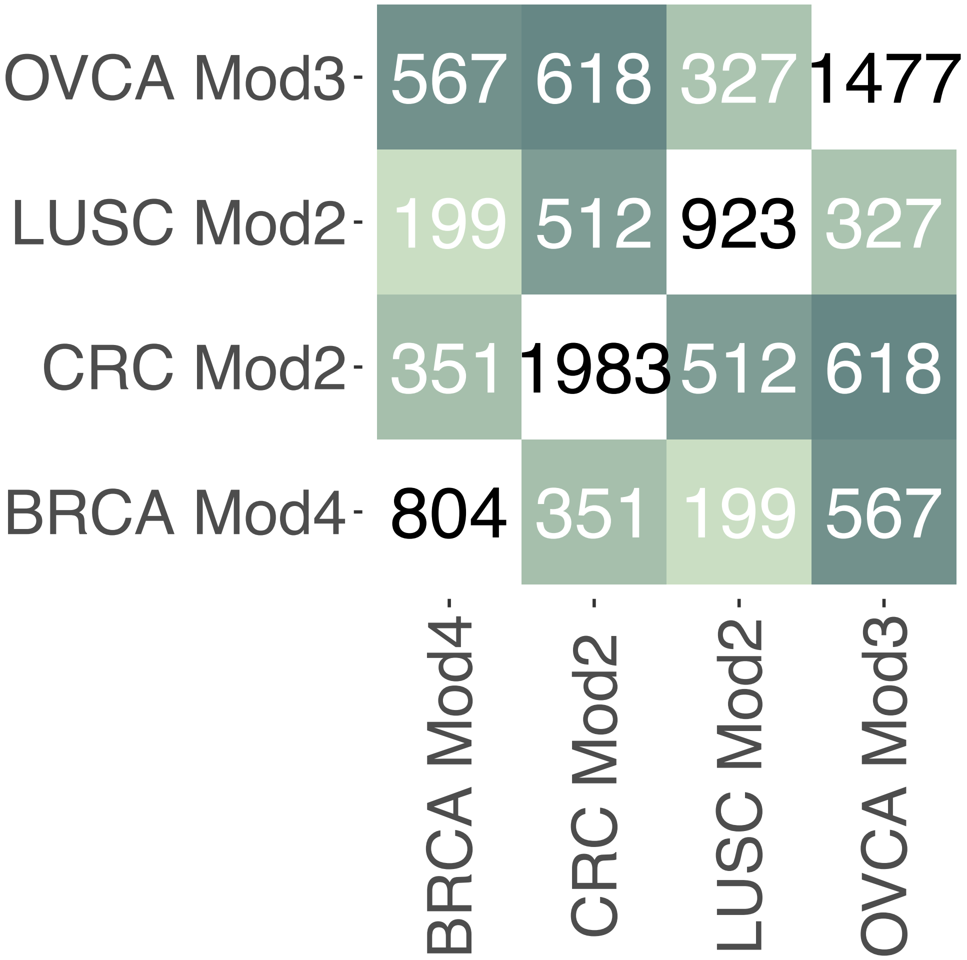

Figure S14 The spatial distribution of spMOCA's tumorigenesis-related module in each cancer data. Each spot is colored by the module score, with orange colors indicating higher scores.

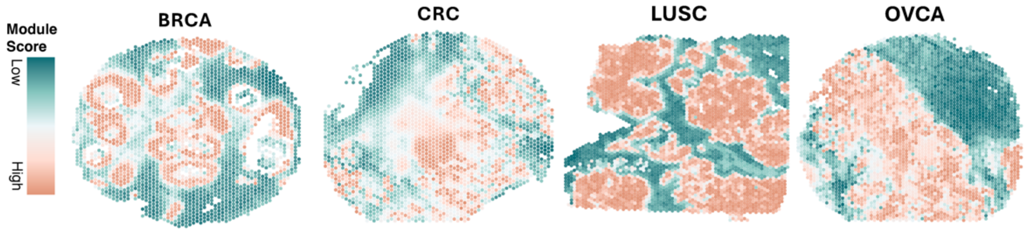

Figure S15 H&E Image of 10x Visium BRCA data with annotated regions. The annotation map was downloaded from https://www.10xgenomics.com/datasets/human-breast-cancer-ductal-carcinoma-in-situ-invasive-carcinoma-ffpe-1-standard-1-3-0.

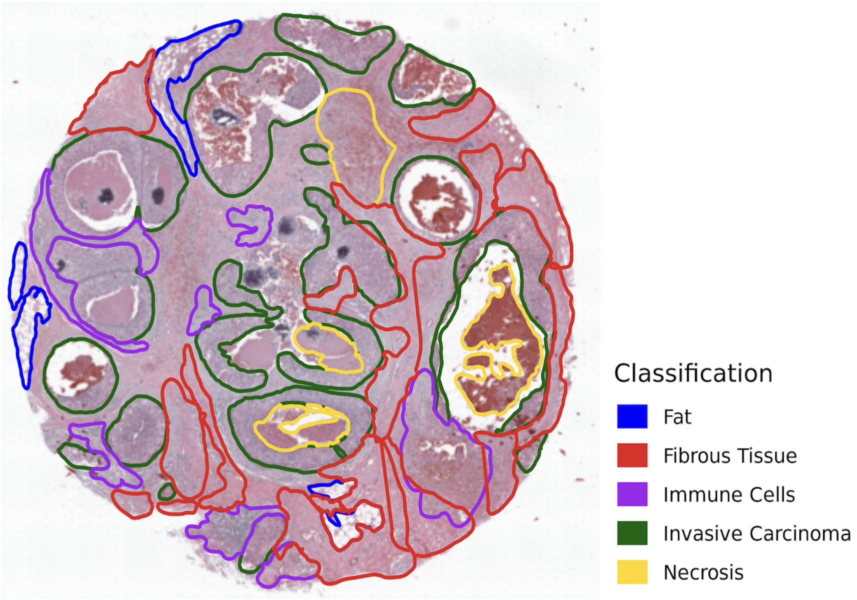

Figure S16 The decomposition of cell-type proportions in 10x Visium BRCA Data. The spots are colored by a level of cell-type proportions. Orange color indicates higher cell-type proportion.

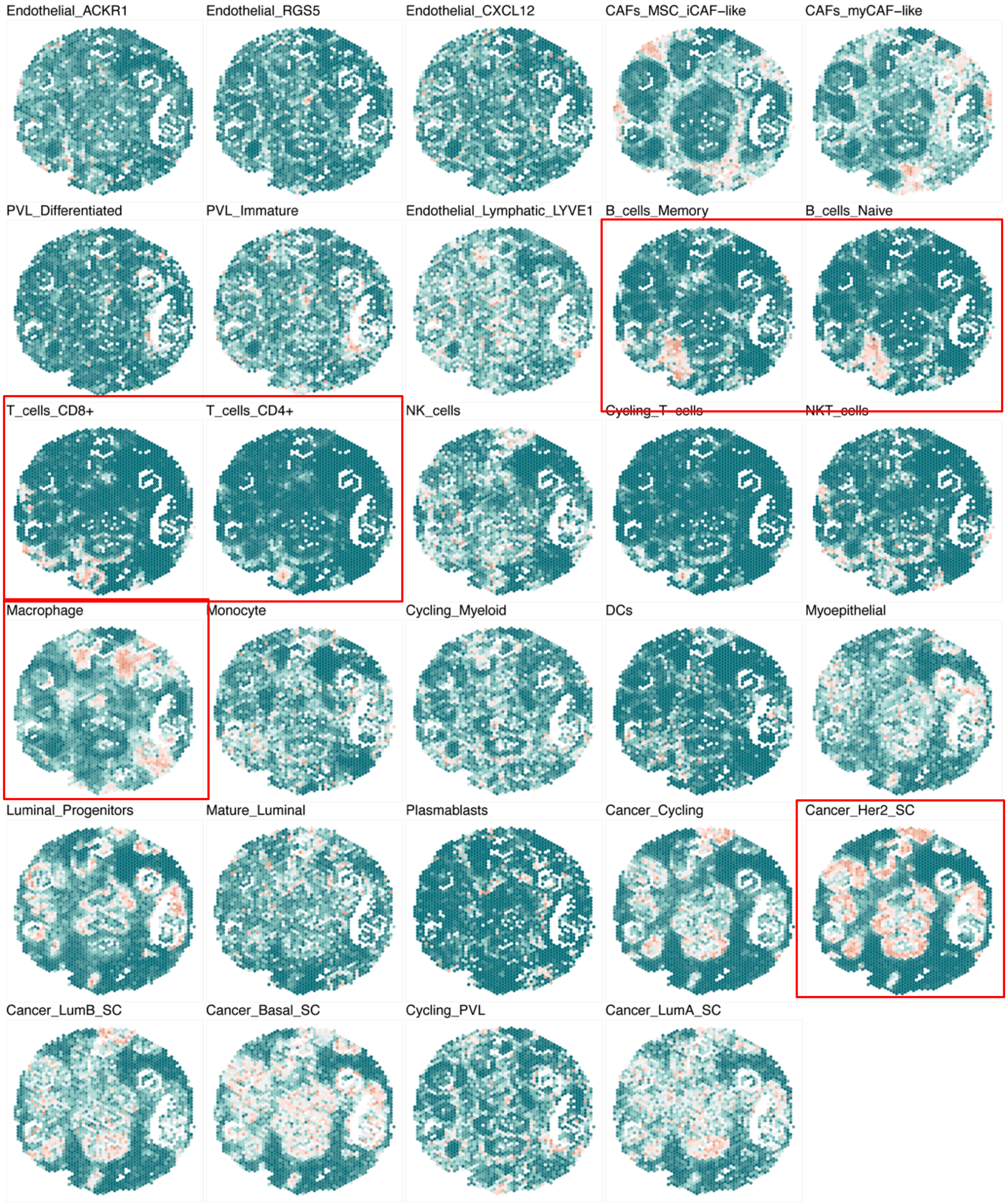

Figure S17 The decomposition of cell-type proportions in 10x Visium CRC Data. The spots are colored by a level of cell-type proportions. Orange color indicates higher cell-type proportion.

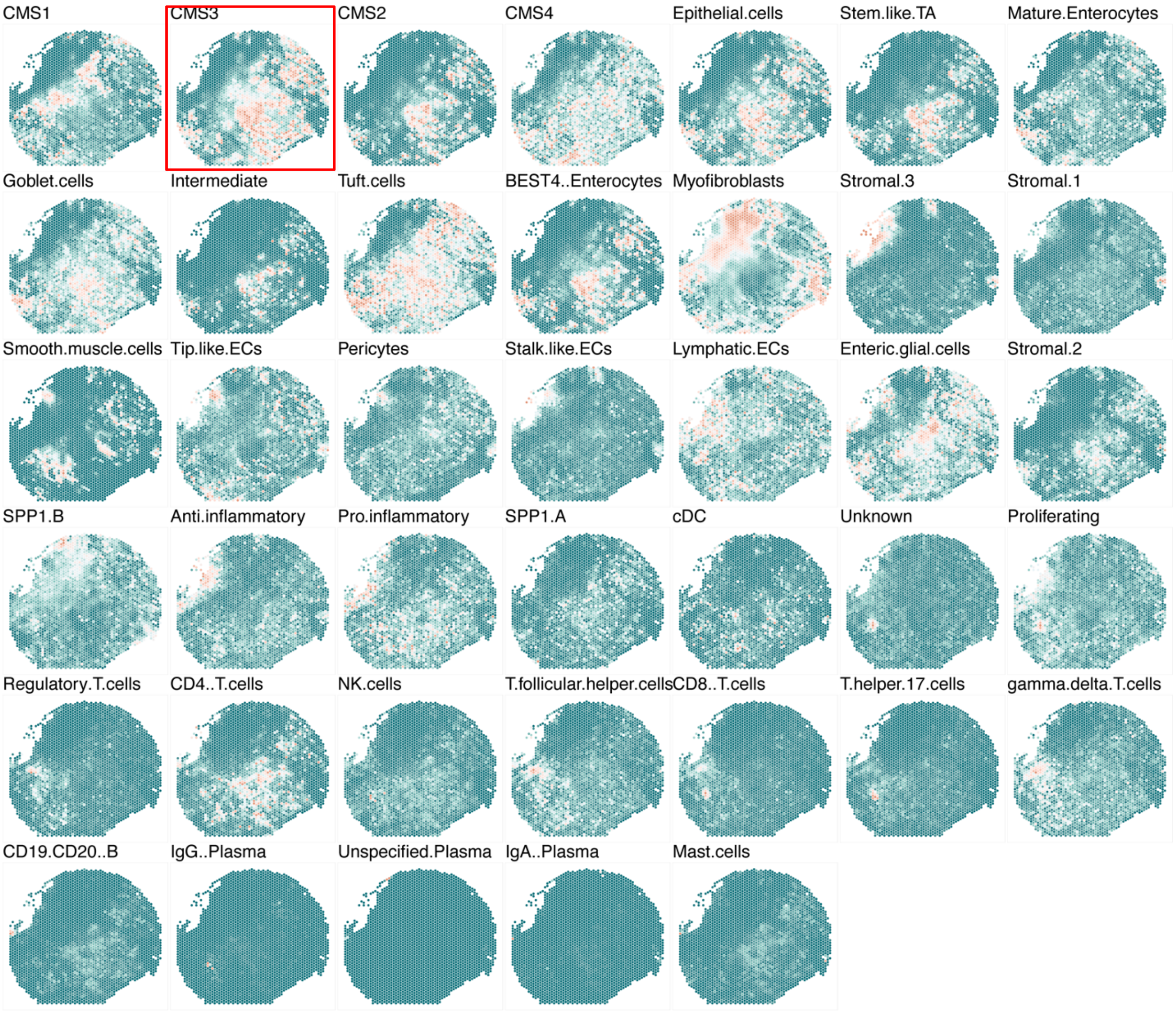

Figure S18 The decomposition of cell-type proportions in 10x Visium LUSC Data. The spots are colored by a level of cell-type proportions. Orange color indicates higher cell-type proportion.

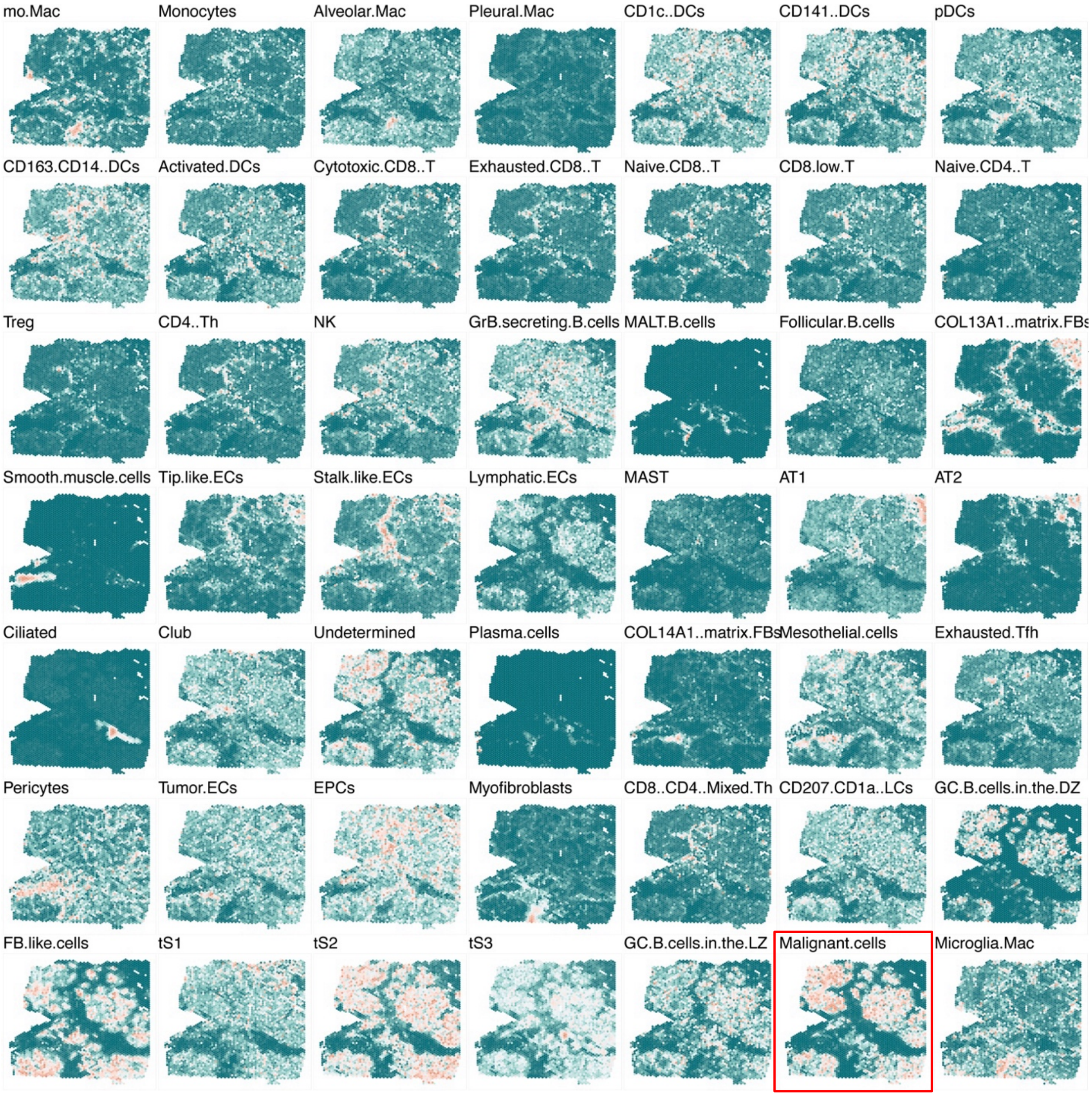

Figure S19 The decomposition of cell-type proportions in 10x Visium OVCA Data. The spots are colored by a level of cell-type proportions. Orange color indicates higher cell-type proportion.

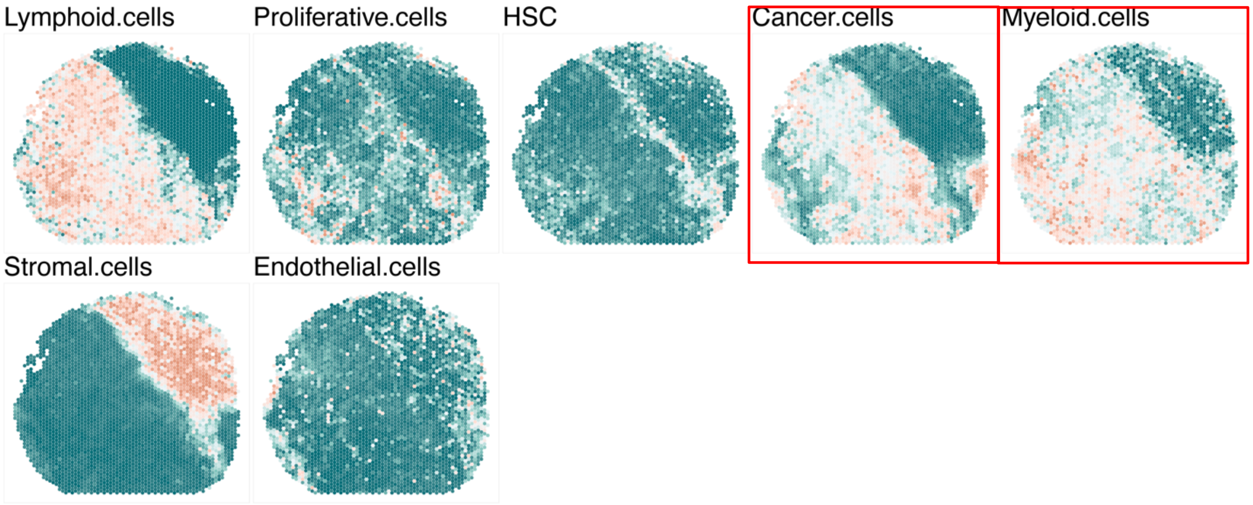

Figure S20 The cross-tumor analysis of the immune-related gene modules in spMOCA gene co-expression networks. (A) Selected significant pathways shared across all spMOCA's immune-related modules. **(B)** Top 10 hub genes in spMOCA's immune -related modules. **(C)** The distribution of spMOCA's immune-related modules' scores over a tissue space. Each spot is colored by the module score, with orange colors indicating higher scores. (**D**) Overlap between TCGA-derived tumor prognostic genes and spMOCA immune-related hub genes. Green coloring indicates that the gene is identified as a hub gene by spMOCA only; Yellow coloring indicates that the gene is identified as a prognostics gene from TCGA data only; Red coloring indicates that the gene is identified as a hub gene by spMOCA and as a TCGA-derived tumor prognostic gene.

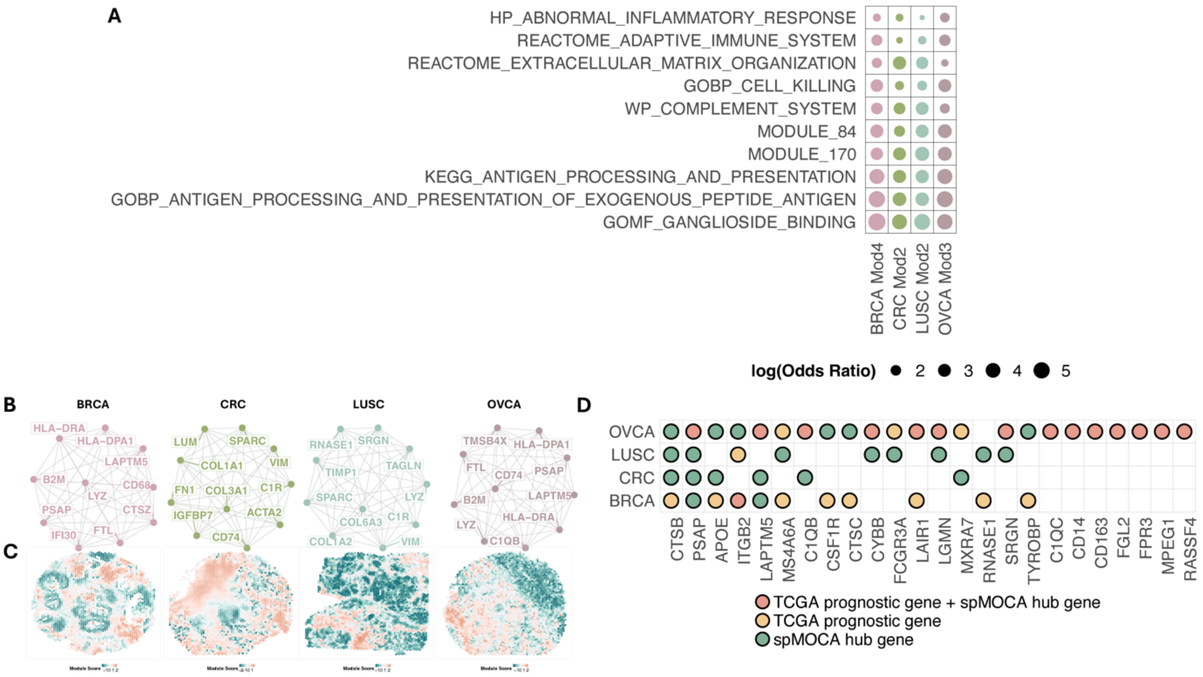

Figure S21 Tumorigenesis-related modules from GCNs of Pearson's correlation, Giotto and CSCORE across all 10x Visium tumor data. (A) Pairwise counts of shared pathways across all Pearson's correlation's, Giotto's and CSCORE's tumorigenesis-related modules. **(B)** The distribution of Pearson's correlation's, Giotto's and CSCORE's tumorigenesis-related modules' scores over tumor tissue spaces. Each spot is colored by the module score, with orange colors indicating higher scores.

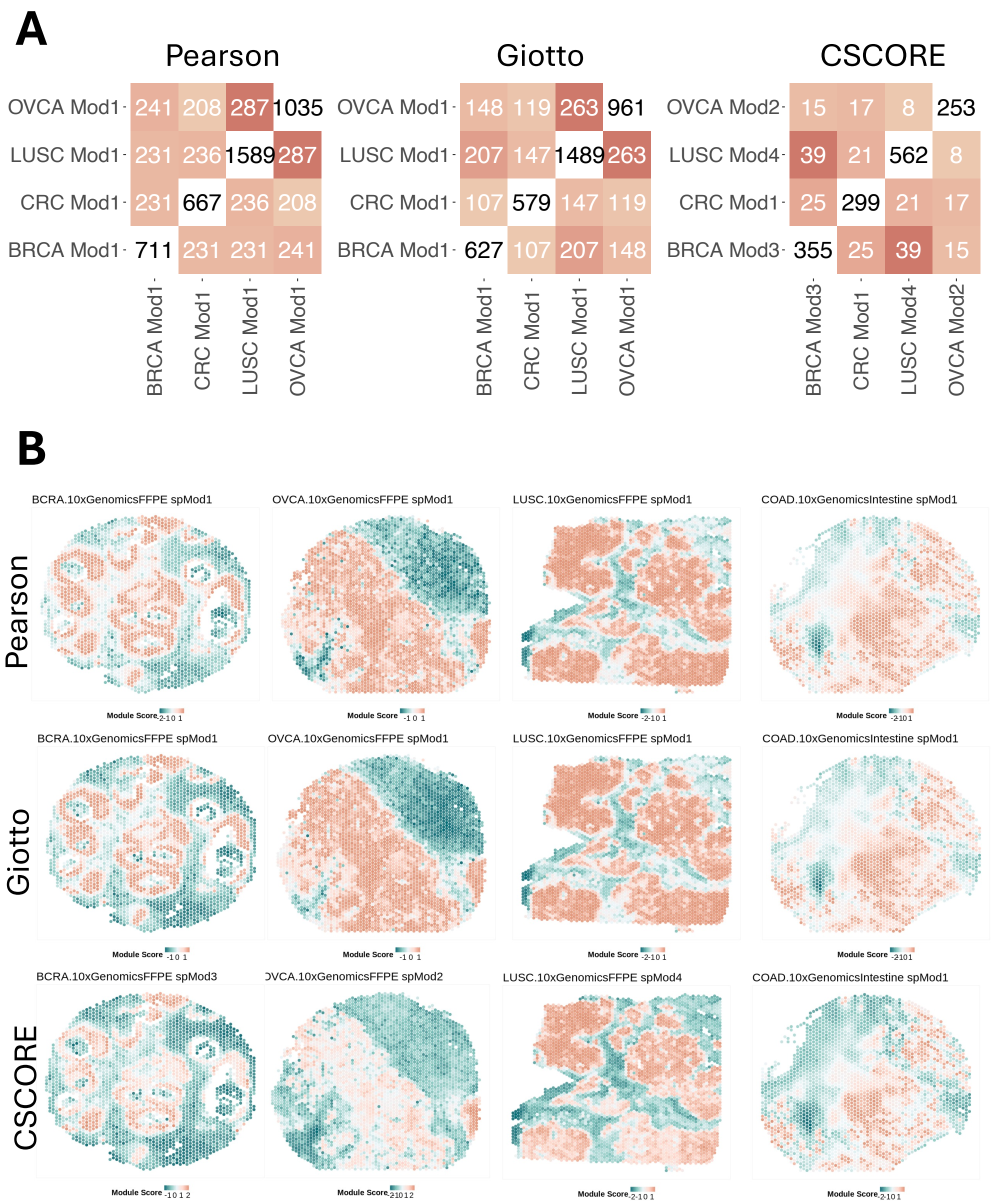

Figure S22 The counts of overlaps between the prognostic genes extracted from TCGA data and the tumorigenesis-related modules'/immune-related modules' top hub genes.

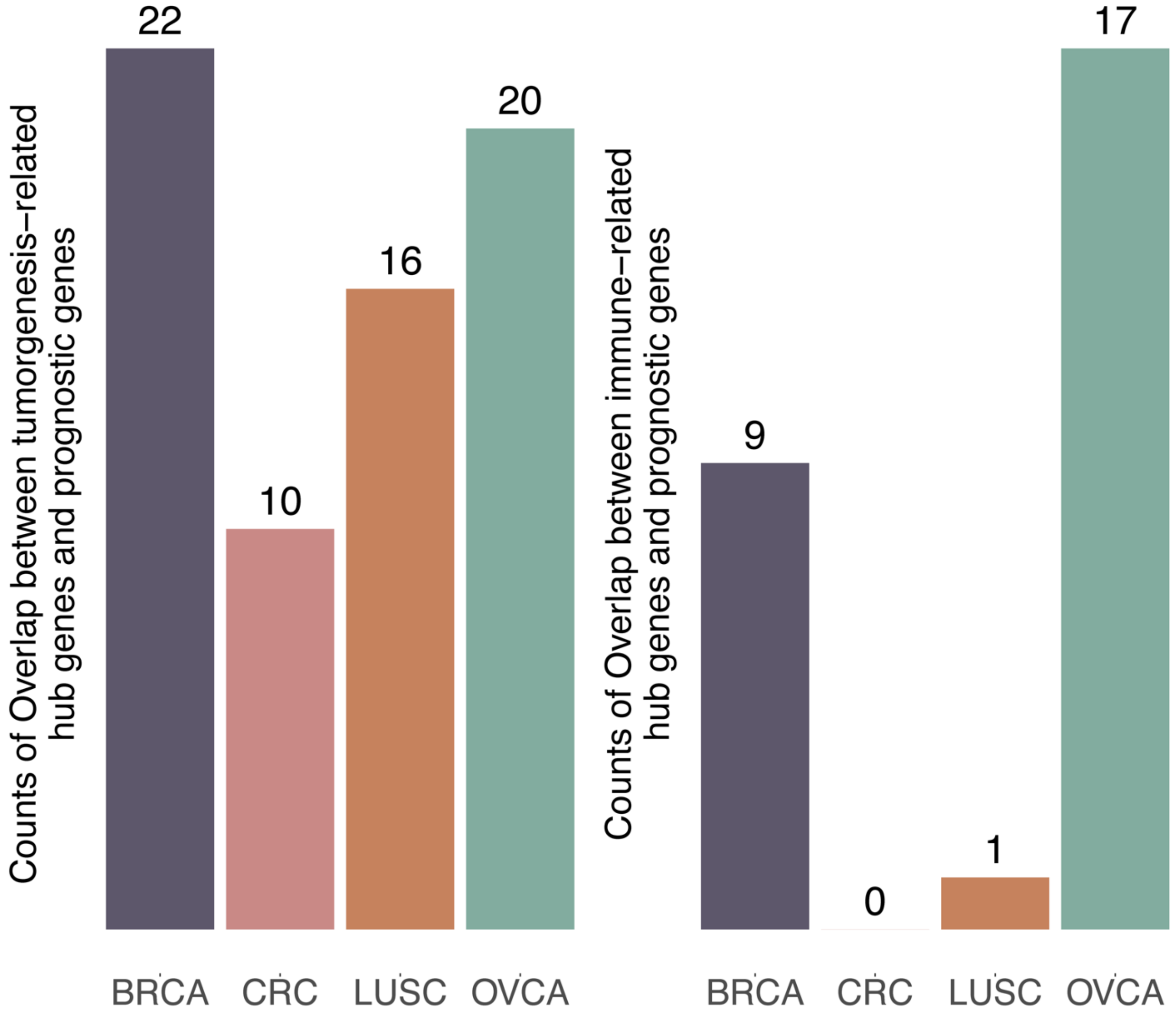

Figure S23 The between-module variance and Davies-Bouldin's index of gene modules in 10x Visium aging mouse brain data for different age stages. Here, a high between-module variation represents that gene modules have large separation in their expression patterns, and a low Davies-Bouldin's index represents that gene modules have low similarity.

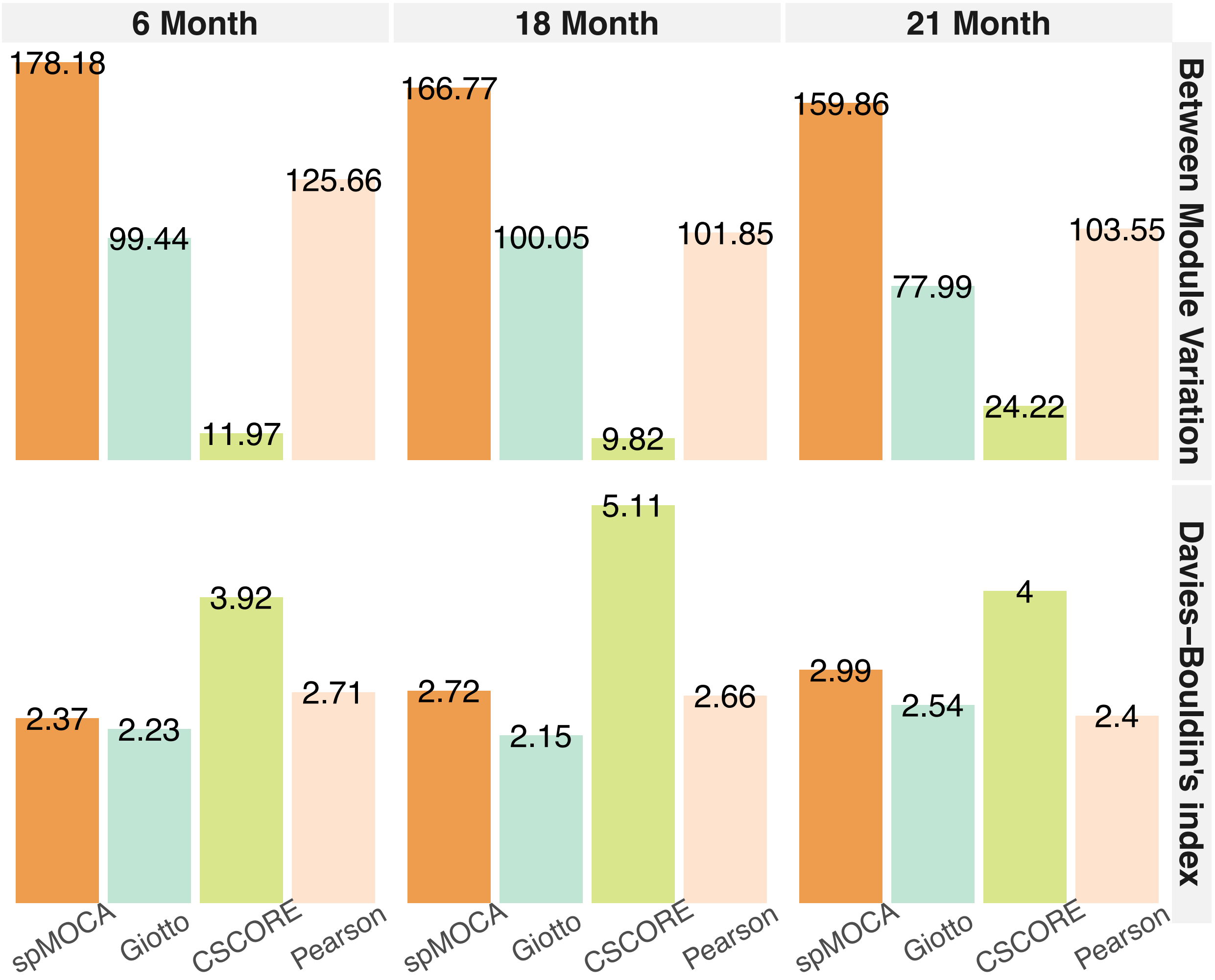

Figure S24 The dissimilarity of gene modules in aging-related pathways in 10x Visium aging mouse brain data. The boxplots show modules' hamming distance based on their associated pathways**.** The pathways are the pathways gene sets derived from aging-related pathways. P-values are calculated through two-sided Wilcoxon rank sum test. *: p-value < 0.05; **: p-value < 0.01; ***: p-value < 0.001; ****: p-value < 10^{-4}

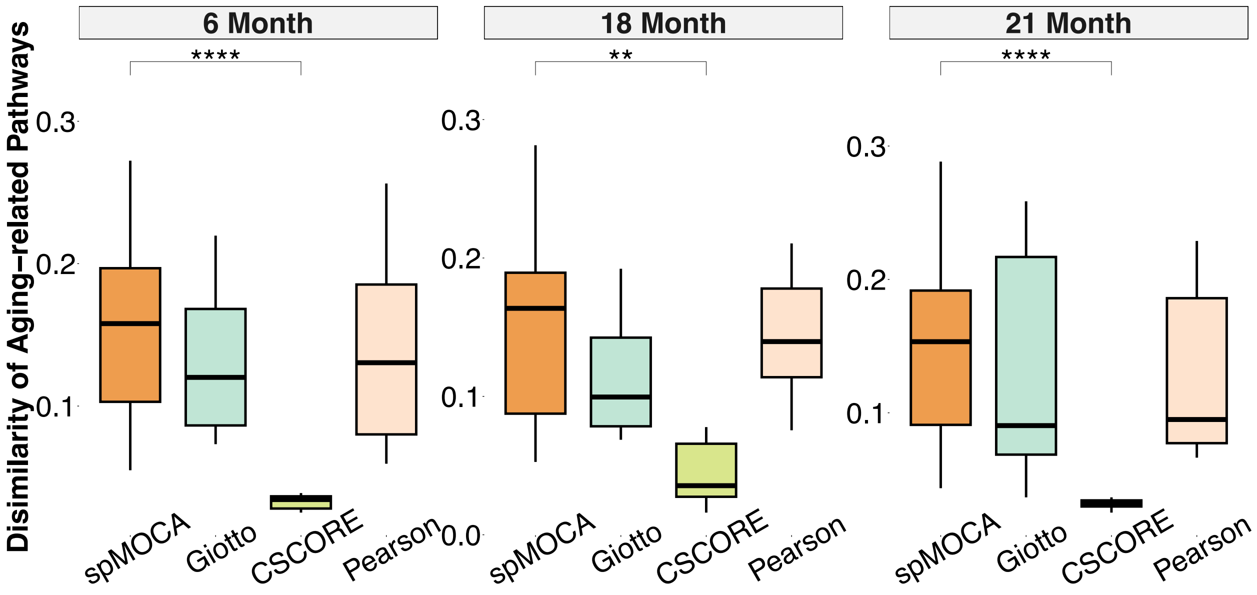

Figure S25 Gene co-expression networks in mouse and human MERFISH brain cortex data generated by CS-CORE. Figures show all shared genes between two species with a specific color indicating their module membership. For human data, dark red for Human Mod1, light red-orange for Human Mod2, dark indigo for Human Mod3, light green for Human Mod4 and light yellow-orange for Human Mod5. For mouse data, dark cyan-blue for Mouse Mod1, light muted pink for Mouse Mod2, light orange for Mouse Mod3, dark indigo for Mouse Mod4 and dark red for Mouse Mod5.

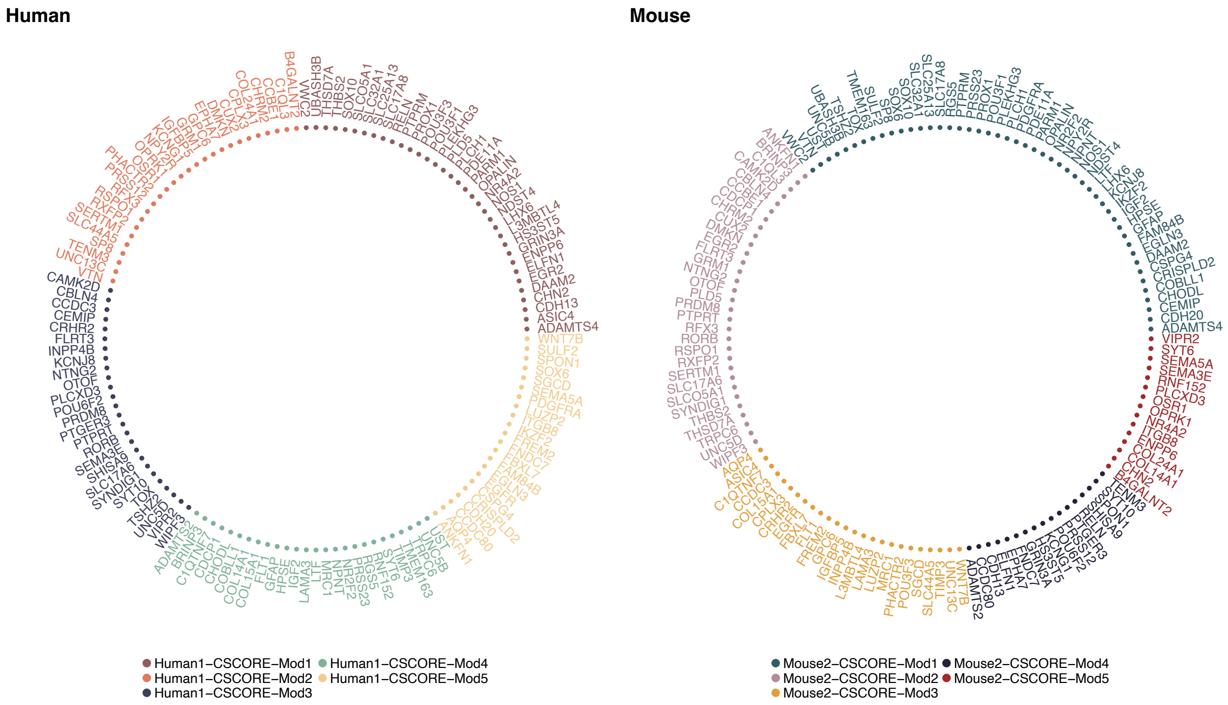

Figure S26 Gene co-expression networks in mouse and human MERFISH brain cortex data generated by Giotto. Figures show all shared genes between two species with a specific color indicating their module membership. For human data, dark red for Human Mod1, light red-orange for Human Mod2, dark indigo for Human Mod3, light green for Human Mod4 and light yellow-orange for Human Mod5. For mouse data, dark cyan-blue for Mouse Mod1, light muted pink for Mouse Mod2, light orange for Mouse Mod3, dark indigo for Mouse Mod4 and dark red for Mouse Mod5.

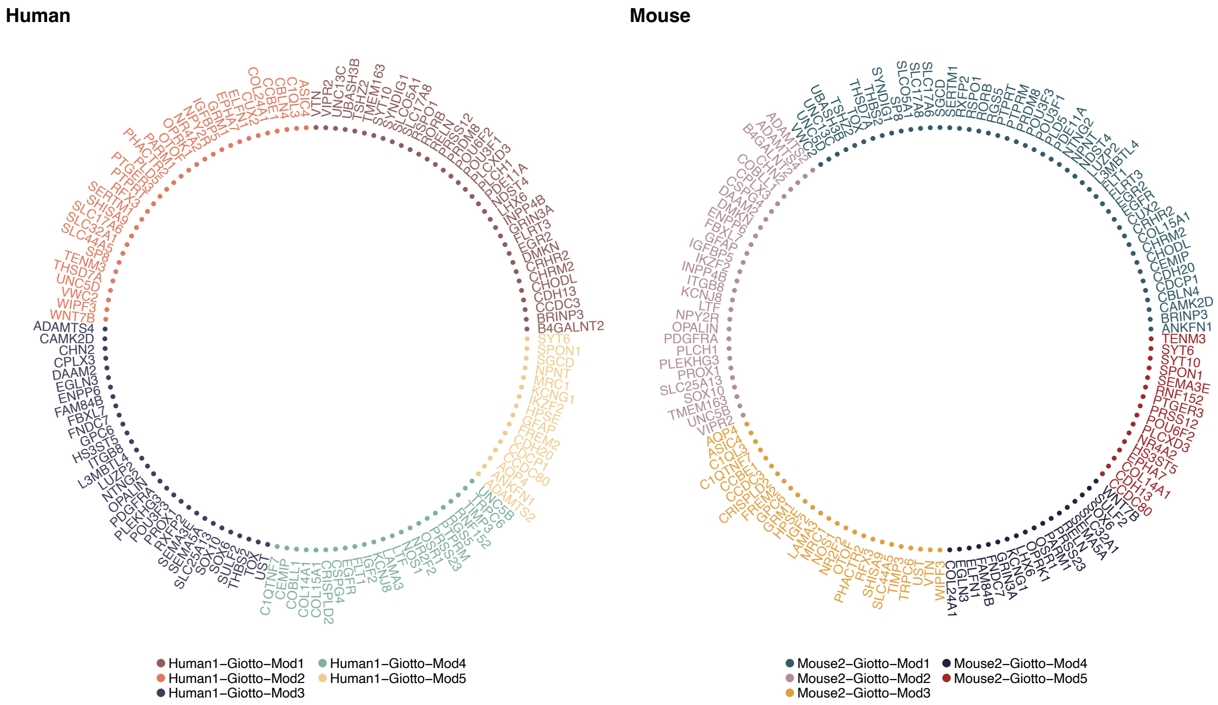

Figure S27 Gene co-expression networks in mouse and human MERFISH brain cortex data generated by Pearson's Correlation. Figures show all shared genes between two species with a specific color indicating their module membership. For human data, dark red for Human Mod1, light red-orange for Human Mod2, dark indigo for Human Mod3, light green for Human Mod4 and light yellow-orange for Human Mod5. For mouse data, dark cyan-blue for Mouse Mod1, light muted pink for Mouse Mod2, light orange for Mouse Mod3, dark indigo for Mouse Mod4 and dark red for Mouse Mod5.

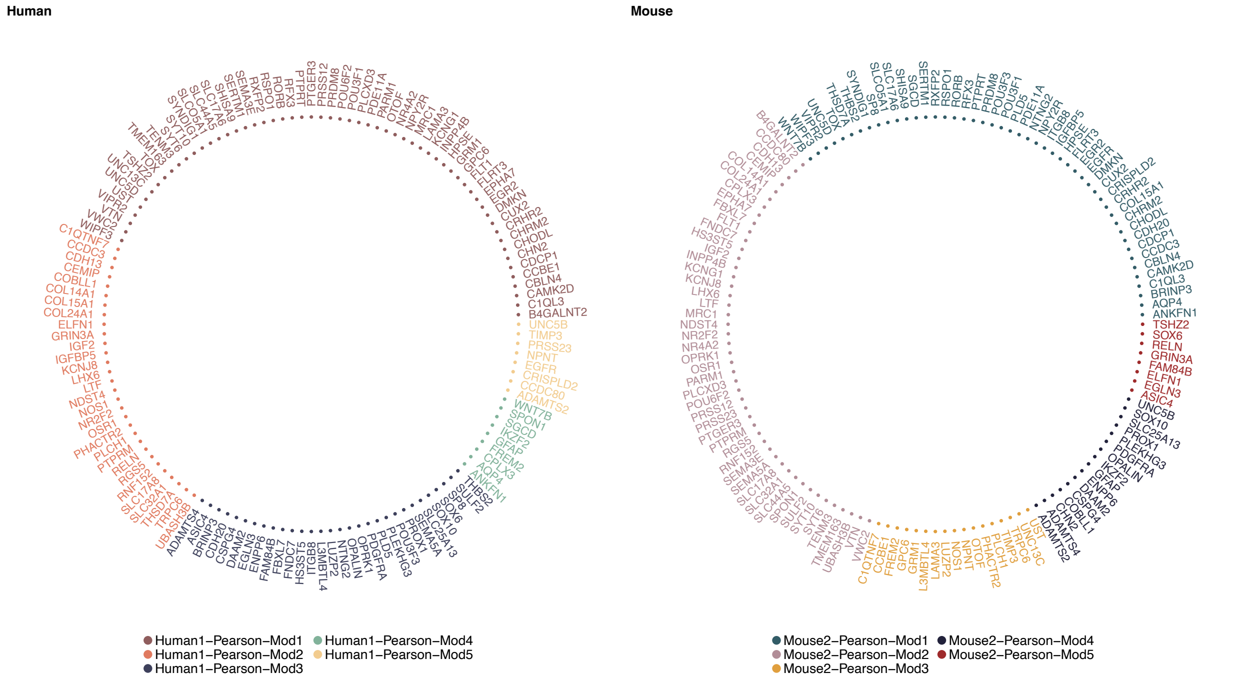

Figure S28 The between-module variance and Davies-Bouldin's index of gene modules in MERFISH mouse cortex data. Here, a high between-module variation represents that gene modules have large separation in their expression patterns, and a low Davies-Bouldin's index represents that gene modules have low similarity.

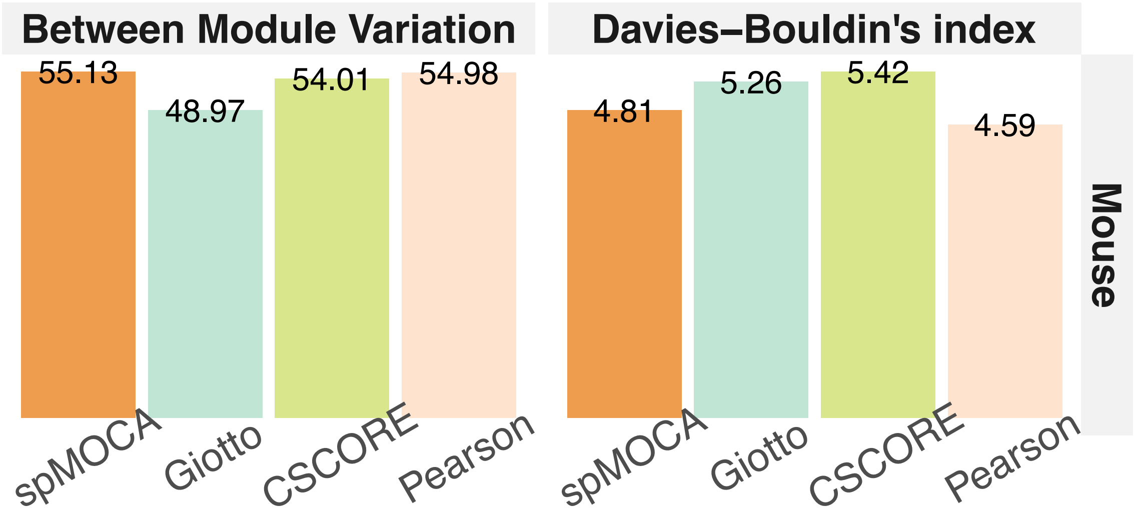

Figure S29 The separation of gene modules in brain cell-type related gene pathways in MERFISH human and mouse cortex data. The boxplots show modules' hamming distance based on their associated pathways. The pathways are the pathways gene sets derived from cell-type specific pathways. P-values are calculated through two-sided Wilcoxon rank sum test. *: p-value < 0.05; **: p-value < 0.01; ***: p-value < 0.001; ****: p-value < 10^{-4}

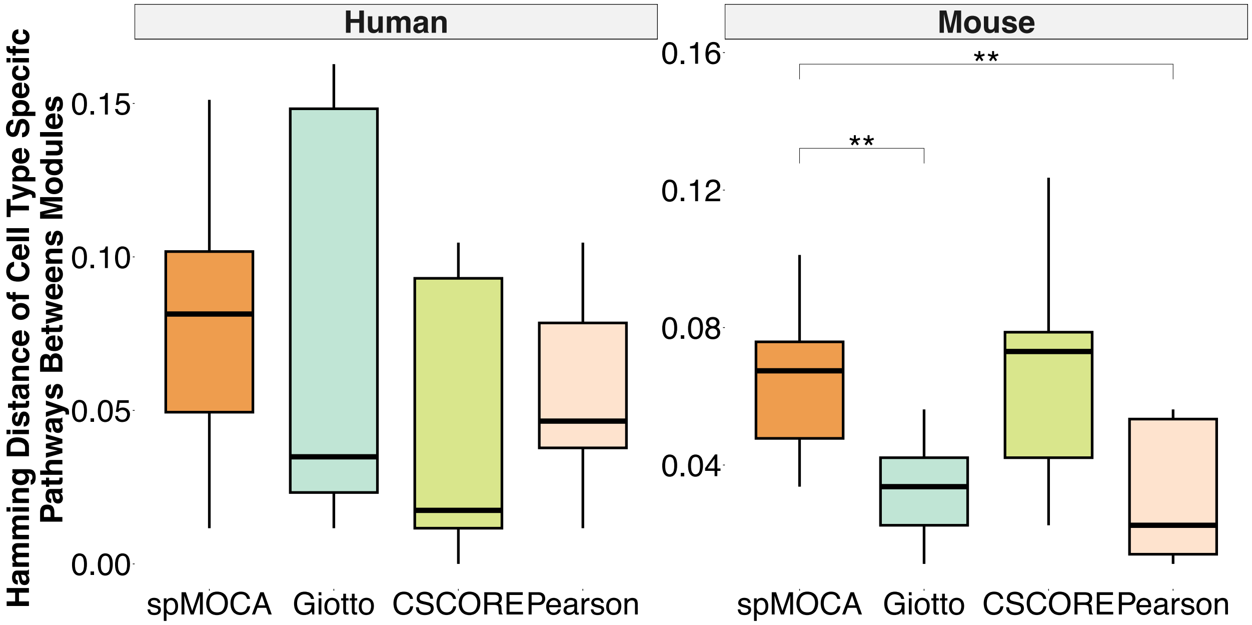

Figure S30 Spatial distribution of selected module scores and their associated cell types in MERFISH human and mouse cortex data. Left: spatial locations of cells with the top 10–20% module scores. Right: spatial locations of the corresponding highly expressing cell types shown in Figure 5C and 5D. For each module, the threshold for "high module score" is adjusted so that the number of selected cells on the left-hand side matches the number of cells assigned to the corresponding cell type on the right hand side.

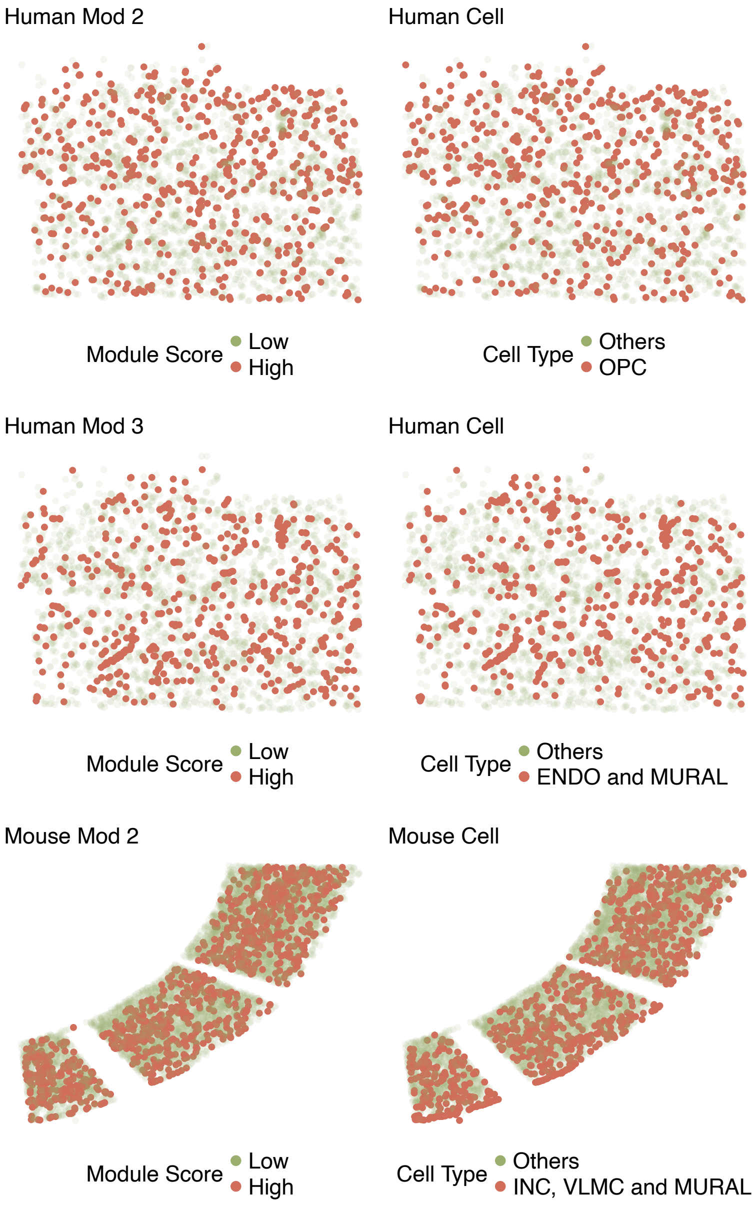

Supplementary Table

Table S1 The evaluation of gene co-expression estimation methods under the scenario of gene-spatial interactive dependency. The 1st column is the spatial dependency simulation scenario. The 2nd column is the method for gene co-expression estimations. The 3rd to 7th is the evaluation metrices: Root of mean squared error (RMSE), median absolute deviation (MAD), Pearson's correlation coefficient (PCC), RV coefficients (RV Coefficients) and WCGNA Adjusted Rand Index (ARI). The best two methods for each scenario are highlighted by red color.

Spatial	Method	RMSE	MAD	PCC	RV Coefficients	WC ARI
**Weak**	spMOCA	0.059	0.040	0.932	0.962	0.584
**Weak**	spMOCA (Est. Bandwidth)	0.059	0.040	0.932	0.963	0.591
**Weak**	Pearson	0.173	0.119	0.643	0.688	0.497
**Weak**	SpaceX	0.365	0.287	0.381	0.692	0.173
**Weak**	Giotto	0.219	0.148	0.449	0.592	0.242
**Weak**	CS-CORE	0.210	0.145	0.411	0.687	0.227
**Moderate**	spMOCA	0.063	0.042	0.927	0.956	0.620
**Moderate**	spMOCA (Est. Bandwidth)	0.116	0.078	0.801	0.857	0.679
**Moderate**	Pearson	0.224	0.159	0.611	0.632	0.345
**Moderate**	SpaceX	0.391	0.301	0.260	0.497	0.118
**Moderate**	Giotto	0.250	0.173	0.467	0.616	0.259
**Moderate**	CS-CORE	0.246	0.177	0.378	0.662	0.207
**Strong**	spMOCA	0.059	0.040	0.930	0.957	0.696

**Strong**	spMOCA (Est. Bandwidth)	0.135	0.091	0.774	0.785	0.621
**Strong**	Pearson	0.293	0.211	0.462	0.436	0.270
**Strong**	SpaceX	0.440	0.349	0.234	0.346	0.139
**Strong**	Giotto	0.301	0.213	0.356	0.430	0.136
**Strong**	CS-CORE	0.306	0.225	0.314	0.482	0.109

Table S2 The evaluation of gene co-expression estimation under the scenario of gene-spatial additive dependency. The 1st column is the spatial dependency simulation scenario. The 2nd column is the method of gene co-expression estimations. The 3rd to 7th is the evaluation metrices: Root of mean squared error (RMSE), median absolute deviation (MAD), Pearson's correlation coefficient (PCC), RV coefficients (RV Coefficients) and WCGNA Adjusted Rand Index (ARI). The best three methods for each scenario are highlighted by red color.

Spatial	Method	RMSE	MAD	PCC	RV Coefficients	WC ARI
**Weak**	spMOCA	0.151	0.103	0.718	0.771	0.550
**Weak**	spMOCA (Est. Bandwidth)	0.151	0.103	0.718	0.771	0.543
**Weak**	Pearson	0.134	0.085	0.610	0.762	0.311
**Weak**	SpaceX	0.303	0.230	0.483	0.853	0.369
**Weak**	Giotto	0.239	0.155	0.105	0.116	0.015
**Weak**	CS-CORE	0.170	0.074	0.306	0.626	0.069
**Moderate**	spMOCA	0.093	0.062	0.857	0.907	0.694
**Moderate**	spMOCA (Est. Bandwidth)	0.150	0.101	0.710	0.774	0.642
**Moderate**	Pearson	0.145	0.093	0.563	0.656	0.174
**Moderate**	SpaceX	0.303	0.226	0.516	0.853	0.322
**Moderate**	Giotto	0.258	0.171	0.097	0.085	0.007
**Moderate**	CS-CORE	0.171	0.079	0.310	0.615	0.088
**Strong**	spMOCA	0.076	0.051	0.895	0.919	0.623
**Strong**	spMOCA (Est. Bandwidth)	0.151	0.103	0.706	0.773	0.586

**Strong**	Pearson	0.164	0.106	0.510	0.485	0.091
**Strong**	SpaceX	0.291	0.212	0.535	0.844	0.303
**Strong**	Giotto	0.290	0.198	0.083	0.057	0.006
**Strong**	CS-CORE	0.174	0.088	0.320	0.554	0.078

Table S3 List of all spatial transcriptomics data and scRNA-seq data used in in our analysis. scRNA-seq data sets were used as the references for cell type decomposition. The table contains data set name (1st column), tissue (2nd column), experimental protocol/platform (3rd column), year of publication (4th column), number of genes (5th column), number of spatial locations/cells (6th column), data type (7th column) and H&E image affiliation (8th column). GSEXXX represents the GEO accession number that the corresponding dataset can be downloaded from the NCBI GEO website.

Data Set	Tissue	Protocol	Year	# Genes	# Spatial Location	Data Type	H&E Staining
**10x Visium Tumor Data** (*Link1)	Human Breast Cancer	10x Visium	2021	17943	2518	Spatial	*Link4
	Human Colorectal Cancer	10x Visium	2022	17943	2660	Spatial	NA
	Human Lung Cancer	10x Visium	2022	18085	3858	Spatial	NA
	Human Ovarian Cancer	10x Visium	2022	17943	3455	Spatial	NA
**Hahn et al. (GSE212903)**	6-month Mouse Whole Brain	10x Visium	2023	32285	2573	Spatial	NA
	12-month Mouse Whole Brain	10x Visium	2023	32285	2735	Spatial	NA
	18-month Mouse Whole Brain	10x Visium	2023	32285	2973	Spatial	NA
**Fang et al.** (*Link2)	Mouse Cortex	MERFISH	2022	234	6752	Spatial	NA
	Human Cortex	MERFISH	2022	3999	3044	Spatial	NA
**Wu et al. [1] (GSE176078)**	Human Breast Cancer	10x Chromium	2021	29733	100064	scRNA-seq	NA
**Lee et al. [2] (GSE144735)**	Human Colorectal Cancer	10x Chromium	2020	33694	27414	scRNA-seq	NA
**Kim et al. [3] (GSE131907)**	Human Lung Cancer	10x Chromium	2019	29634	208506	scRNA-seq	NA
**Zheng et al. [4]** (*Link3)	Human Ovarian Cancer	10x Chromium	2022	27127	223363	scRNA-seq	NA

**Link1*: https://www.10xgenomics.com/datasets

Link2: https://datadryad.org/stash/dataset/doi:10.5061/dryad.x3ffbg7mw

Link3: https://data.mendeley.com/datasets/rc47y6m9mp/1

Link4:https://www.10xgenomics.com/datasets/human-breast-cancer-ductal-carcinoma-in-situ-invasive-carcinoma-ffpe-1-standard-1-3-0

Table S4. Summary of curated context-specific transcription factors (TFs) from KnockTF and TRRUST databases. Each row corresponds to a dataset analyzed in the main manuscript. The first column lists the dataset names. The second column reports the number of curated TFs identified. The third column provides the reference database used to guide the identification of tissue- or condition-specific TFs. The fourth column reports the number of tissue-specific TFs under specific condition provided by the human TFMarker[5] or and Mouse TF Atlas[6] in each data set. For example, there are 11 human breast cancer specific TFs reported in TFMarker. The fifth column indicates how many of these TFs have known target genes (TGs) in the dataset.

Data	# of TFs from KnockTF + TRRUST in data	TF Reference	# of tissue-specific + condition-specific TFs	# of specific TFs with Overlap TF-TGs in Data
Visium Human BRCA	377	TFMarker	11	11
Visium Human CRC	498	TFMarker	13	13
Visium Human LUSC	551	TFMarker	15	15
Visium Human OVCA	495	TFMarker	7	7
Visium Aging Mouse	80	Mouse TF Atlas	15	5
MERFISH Human Brain	155	TFMarker	6	4
MERFISH Mouse Brain	19	Mouse TF Atlas	9	1

Supplementary Note

1. Methodological details of spMOCA

1.1 The marginal distribution of gene-specific expression profiles and location-specific expression profiles

The matrix normal distribution of the normalized gene expression matrix $\boldsymbol{Y}$ described in equation (3) in Material and Methods also infers the rows and columns following multivariate normal distribution: the ith row of the normalized expression matrix follows

$$\boldsymbol{Y}_{i+} \sim N(\boldsymbol{M}_{i+}, U_{ii}\boldsymbol{V}) \forall i \in 1,2 \dots, G \quad (1)$$

where $\boldsymbol{M}_{i+}$ is the mean vector for the ith row and U_{ii} is the ith diagonal term of $\boldsymbol{U}$; similarly, the jth column of the normalized expression matrix follows

$$\boldsymbol{Y}_{+j} \sim N\left(\boldsymbol{M}_{+j}, V_{jj}\boldsymbol{U}\right) \forall j \in 1,2 \dots, N \quad (2)$$

where $\boldsymbol{M}_{+j}$ is the mean vector for the jth column and V_{jj} is the jth diagonal term of $\boldsymbol{V}$. In the context of spatial transcriptomics, for each gene, the covariance between its spatial expressions is scaled by a gene-specific factor. Similarly, for each spot, the covariance between gene expressions in the spot are scaled by a spot-specific factor.

1.2 Formula of the Silverman's rule-of-thumb estimator

To estimate the bandwidth parameter τ that will be used to construct the spatial kernel, we followed the same procedure in the spatialPCA paper [7]. Specifically, for each gene, we estimated a kernel bandwidth using Silverman's rule-of-thumb estimator. To do so, we first scaled the log-normalized count data for each gene to align the gene expression data with the spatial coordinate scale, with both scaled to standard normal distribution. Silverman's rule-of-thumb estimator is defined as:

$$0.9min\left(\hat{\sigma}, \frac{IQR}{1.34}\right)n^{-1/5}$$

where $\hat{\sigma}$ is the standard deviation of the scaled gene expression, *IQR* is the interquartile range of the scaled gene expression, and n represents the number of spatial locations with expression measurements.

2. Description on compared gene co-expression estimation methods

We compared spMOCA with four gene co-expression estimation methods including SpaceX[8], Giotto[9], CSCORE[10] and Pearson's correlation. SpaceX is a Bayesian Poisson-based matrix factorization method to estimate gene co-expression for spatial transcriptomics data. Giotto is a widely used R toolbox for spatial expression data, which integrates with a neighborhood-smooth Pearson's Correlation method. CSCORE is known as an iterative reweighted least squared algorithm for gene co-expression estimation, originally developed for scRNA-seq data. Pearson's correlation, known for its simplicity, is a common tool in gene co-expression. These four methods represent the current strategies for estimating gene co-expression in single-cell RNA-seq and spatial transcriptomics data: methods specifically designed for spatially resolved data (e.g., SpaceX, Giotto), methods developed for scRNA-seq data (e.g., CSCORE), or simply conventional statistical correlation tools (e.g., Pearson's correlation). For the methods such as CS-CORE, Giotto and SpaceX, they require count data as input.

3 Gene co-expression estimation evaluation metric

3.1 Formula of the Adjusted Rand Index (ARI)

ARI is defined as

$$ARI(P,T) = \frac{\sum_{l,s}\binom{n_{ls}}{2} - \left[\sum_l \binom{a_l}{2} \sum_s \binom{b_s}{2}\right] / \binom{n}{2}}{\frac{1}{2}\left[\sum_l \binom{a_l}{2} + \sum_s \binom{b_s}{2}\right] - \left[\sum_l \binom{a_l}{2} \sum_s \binom{b_s}{2}\right] / \binom{n}{2}}$$

Where $P = (p_1, p_2, \dots p_G)^T$ denotes the inferred gene module labels from WGCNA while $T = (t_1, t_2, \dots t_G)^T$ denotes the original gene module labels assigned during the data generation. l and s enumerate the gene modules, with $l = 1,2,3, \dots, r$ and $l = 1,2,3, \dots, k$ where r and k are the number of inferred gene modules and the number of original gene modules. $n_{ls} = \sum_{ij} I(p_i = l) I(t_j = s)$ is the number of times where the i-th gene belongs to the module l in the inferred module labeling and j-th gene belongs to the module s in the original module labeling; note that n_{ls} is an entry of contingency table which effectively measures the number of genes that are in common between P and T, with $I()$ being an indicator function. $a_l = \sum_s n_{ls}$ is the sum of the s-th column of the contingency table; and $b_s = \sum_l n_{ls}$ is the sum of l-th row of the contingency table; $\binom{*}{*}$ denotes a binomial coefficient.

4. Evaluation recovery of transcription factor–target gene (TF–TG) interactions from gene co-expression network

we validate biological relevance of a gene co-expression network (GCN) with experimentally supported transcription factor–target gene (TF–TG) interactions from two widely cited databases - TTRUSTV2 (https://www.grnpedia.org/trrust/) and KnockTF (https://bio.liclab.net/KnockTFv2/). These datasets [11, 12] include regulatory interactions measured in the same tissue types relevant to our spatially resolved transcriptomics (SRT) datasets, such as human brain, breast, colon, ovary, and lung, as well as mouse brain. We curated high-confidence TF–TG pairs from these databases, retaining those with known regulatory direction (TRRUST) or statistically significant TF-TG interactions (KnockTF, adjusted $p < 0.05$). While we attempted to access ChIP-Atlas, we were unable to retrieve downloadable TF–TG mappings and thus focused on TRRUST and KnockTF only. To account for tissue and condition specificity, which is not directly annotated in these databases, we cross-referenced curated TFs with two additional resources: TFMarker [5] for human TFs and Mouse TF Atlas [6] for mouse TFs. These references enabled us to identify TFs that are specific to the biological context (organ and condition) of each SRT dataset we analyzed in this study. The overlap between the curated TFs and the analyzed datasets is summarized in Table S4.

Specifically, for each TF, we identified the top 1–5% of TGs as the genes most strongly correlated with it, ranked by co-expression strength in the network inferred by each method. We then counted how many of its experimentally validated TGs appeared within this top-ranked set. This count was used to define the recovery of known TF–TG regulatory relationships, as shown in Table R1. Our rationale is that a biologically meaningful co-expression network should place validated TGs among the most strongly associated genes of their corresponding TFs, thereby enriching for true regulatory targets and reducing the likelihood of false-positive associations. This validation was performed across four 10X Visium human tumor datasets (BRCA, CRC, OVCA, and LUSC), we well as the 6-month mouse brain dataset, which represents the closest available match to the developmental stages (2–8 weeks) used in the TF–TG reference databases[13, 14]. In the MERFISH datasets, we focused only on the human brain dataset, as the corresponding mouse dataset included a limited gene panel, resulting in no overlapping TF–TG pairs with the reference databases.

Across all datasets, we compared CSCORE, Giotto and Pearson correlation networks. Because transcription factors (TFs) vary widely in the number of known target genes (TGs) they regulate, we assessed each method's performance by counting how often it achieved the highest recovery for individual TFs, rather than relying on total TF–TG

counts. This per-TF ranking approach reduces bias toward TFs with large numbers of known targets and ensures that each TF contributes equally to the evaluation, regardless of its connectivity. As a result, it provides a more balanced and interpretable comparison across methods and datasets, particularly when the number of validated TGs is unevenly distributed.

Reference

1. Wu, S.Z., et al., *A single-cell and spatially resolved atlas of human breast cancers.* Nature Genetics, 2021. **53**(9): p. 1334-1347.
2. Lee, H.-O., et al., *Lineage-dependent gene expression programs influence the immune landscape of colorectal cancer.* Nature Genetics, 2020. **52**(6): p. 594-603.
3. Kim, N., et al., *Single-cell RNA sequencing demonstrates the molecular and cellular reprogramming of metastatic lung adenocarcinoma.* Nature Communications, 2020. **11**(1): p. 2285.
4. Zheng, X., et al., *Single-cell analyses implicate ascites in remodeling the ecosystems of primary and metastatic tumors in ovarian cancer.* Nature Cancer, 2023. **4**(8): p. 1138-1156.
5. Xu, M., et al., *TF-Marker: a comprehensive manually curated database for transcription factors and related markers in specific cell and tissue types in human.* Nucleic Acids Research, 2021. **50**(D1): p. D402-D412.
6. Zhou, Q., et al., *A mouse tissue transcription factor atlas.* Nature Communications, 2017. **8**(1): p. 15089.
7. Shang, L. and X. Zhou, *Spatially aware dimension reduction for spatial transcriptomics.* Nature Communications, 2022. **13**(1): p. 7203.
8. Acharyya, S., X. Zhou, and V. Baladandayuthapani, *SpaceX: gene co-expression network estimation for spatial transcriptomics.* Bioinformatics, 2022. **38**(22): p. 5033-5041.
9. Dries, R., et al., *Giotto: a toolbox for integrative analysis and visualization of spatial expression data.* Genome Biology, 2021. **22**(1): p. 78.
10. Su, C., et al., *Cell-type-specific co-expression inference from single cell RNA-sequencing data.* Nature Communications, 2023. **14**(1): p. 4846.
11. Han, H., et al., *TRRUST v2: an expanded reference database of human and mouse transcriptional regulatory interactions.* Nucleic Acids Research, 2017. **46**(D1): p. D380-D386.
12. Feng, C., et al., *KnockTF 2.0: a comprehensive gene expression profile database with knockdown/knockout of transcription (co-)factors in multiple species.* Nucleic Acids Research, 2023. **52**(D1): p. D183-D193.
13. Matsuda, T., et al., *Pioneer Factor NeuroD1 Rearranges Transcriptional and Epigenetic Profiles to Execute Microglia-Neuron Conversion.* Neuron, 2019. **101**(3): p. 472-485.e7.
14. Miura, H., et al., *Easi-CRISPR for creating knock-in and conditional knockout mouse models using long ssDNA donors.* Nature Protocols, 2018. **13**(1): p. 195-215.
